# Molecular karyotyping by array CGH in a Russian cohort of children with intellectual disability, autism, epilepsy and congenital anomalies

**DOI:** 10.1186/1755-8166-5-46

**Published:** 2012-12-31

**Authors:** Ivan Y Iourov, Svetlana G Vorsanova, Oxana S Kurinnaia, Maria A Zelenova, Alexandra P Silvanovich, Yuri B Yurov

**Affiliations:** 1 Mental Health Research Center, Russian Academy of Medical Sciences, 119152, Moscow, Russia; 2 Institute of Pediatrics and Children Surgery, Ministry of Health of the Russian Federation, 125412, Moscow, Russia; 3 Moscow City University of Psychology and Education, Moscow, Russia

**Keywords:** Array CGH, Intellectual disability, Congenital anomalies, Autism, Epilepsy, Genome variations, Chromosome imbalances, Chromosome abnormalities, Copy number viriations (CNVs)

## Abstract

**Background:**

Array comparative genomic hybridization (CGH) has been repeatedly shown to be a successful tool for the identification of genomic variations in a clinical population. During the last decade, the implementation of array CGH has resulted in the identification of new causative submicroscopic chromosome imbalances and copy number variations (CNVs) in neuropsychiatric (neurobehavioral) diseases. Currently, array-CGH-based technologies have become an integral part of molecular diagnosis and research in individuals with neuropsychiatric disorders and children with intellectual disability (mental retardation) and congenital anomalies. Here, we introduce the Russian cohort of children with intellectual disability, autism, epilepsy and congenital anomalies analyzed by BAC array CGH and a novel bioinformatic strategy.

**Results:**

Among 54 individuals highly selected according to clinical criteria and molecular and cytogenetic data (from 2426 patients evaluated cytogenetically and molecularly between November 2007 and May 2012), chromosomal imbalances were detected in 26 individuals (48%). In two patients (4%), a previously undescribed condition was observed. The latter has been designated as meiotic (constitutional) genomic instability resulted in multiple submicroscopic rearrangements (including CNVs). Using bioinformatic strategy, we were able to identify clinically relevant CNVs in 15 individuals (28%). Selected cases were confirmed by molecular cytogenetic and molecular genetic methods. Eight out of 26 chromosomal imbalances (31%) have not been previously reported. Among them, three cases were co-occurrence of subtle chromosome 9 and 21 deletions.

**Conclusions:**

We conducted an array CGH study of Russian patients suffering from intellectual disability, autism, epilepsy and congenital anomalies. In total, phenotypic manifestations of clinically relevant genomic variations were found to result from genomic rearrangements affecting 1247 disease-causing and pathway-involved genes. Obviously, a significantly lesser part of them are true candidates for intellectual disability, autism or epilepsy. The success of our preliminary array CGH and bioinformatic study allows us to expand the cohort. According to the available literature, this is the first comprehensive array CGH evaluation of a Russian cohort of children with neuropsychiatric disorders and congenital anomalies.

## Background

Chromosomal imbalances and copy number variations (CNVs) are probably the most common genetic causes of intellectual disability (mental retardation) and congenital anomalies. Genome variations involving chromosomal and subchromosomal loci are frequently detected in a wide spectrum of neuropsychiatric disorders. Indeed, these patients seem to need in an evaluation by array comparative genomic hybridization (CGH) or molecular karyotyping. The latter is repeatedly shown to be a powerful tool for the identification of genomic variations (submicroscopic chromosomal imbalances and CNVs) in a clinical population. Moreover, to ensure an adequate diagnostic yield (i.e. >10-15%), molecular diagnosis of constitutional chromosomal and subchromosomal imbalances is recommended to be performed by molecular karyotyping or related array-CGH-based technologies, which has become an important genetic test for patients suffering from intellectual disability (neuropsychiatric diseases) and congenital anomalies. Whole-genome scanning technologies are unique for detecting losses or gains of genomic material and are consistently used for studying genetic causes of postnatal morbidity (i.e. dysmorphology, malformations, developmental delay, intellectual disability, autism, epilepsy, and schizophrenia) providing for the delineation of the molecular mechanisms and causative genes [1-10].

Here, we present the first Russian experience of an array CGH application to a clinical population. The cohort included 54 children with intellectual disability, autism, epilepsy and congenital anomalies highly selected from 2426 patients according to clinical criteria and molecular and cytogenetic data.

## Results

**Figure 1:**
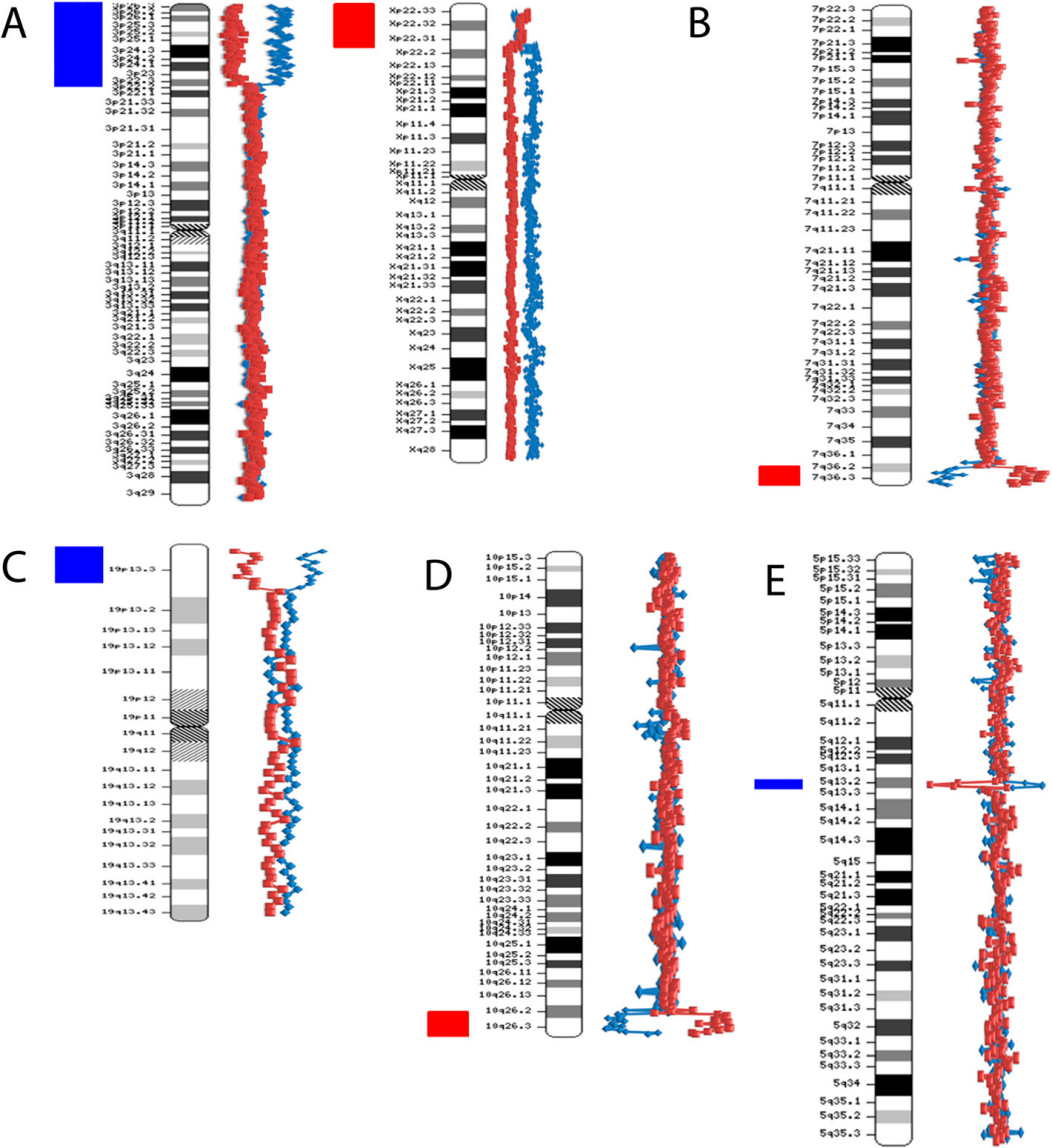
**Examples of array CGH graphical overviews. A.** Case #8: a deletion of chromosome X short arm and a duplication of chromosome 3 short arm — arr Xp22.33p22.2(2,333,897-9,726,574)x1,3p26.3p22.3(200,000-36,550,871)x3 — due to an unbalanced maternal translocation t(X;3) (conventional karyotyping was performed after array CGH analysis). **B.** Case #27: a deletion of chromosome 7 long arm (subtelomeric 7q deletion) — arr 7q36.2q36.3(152,768,630-158,261,821)x1. **C**. Case #18: a duplication of chromosome 19 short arm — arr 19p13.3(260,000-4,953,188)x3. **D**. Case #20: a deletion of chromosome 10 long arm (subtelomeric 10q deletion) — arr 10q26.2q26.3(128,192,760-134,070,099)x1. **E**. Case #40: a duplication of chromosome 5 long arm — arr 5q13.2(68,931,140-72,690,180)x3.

**Figure 2:**
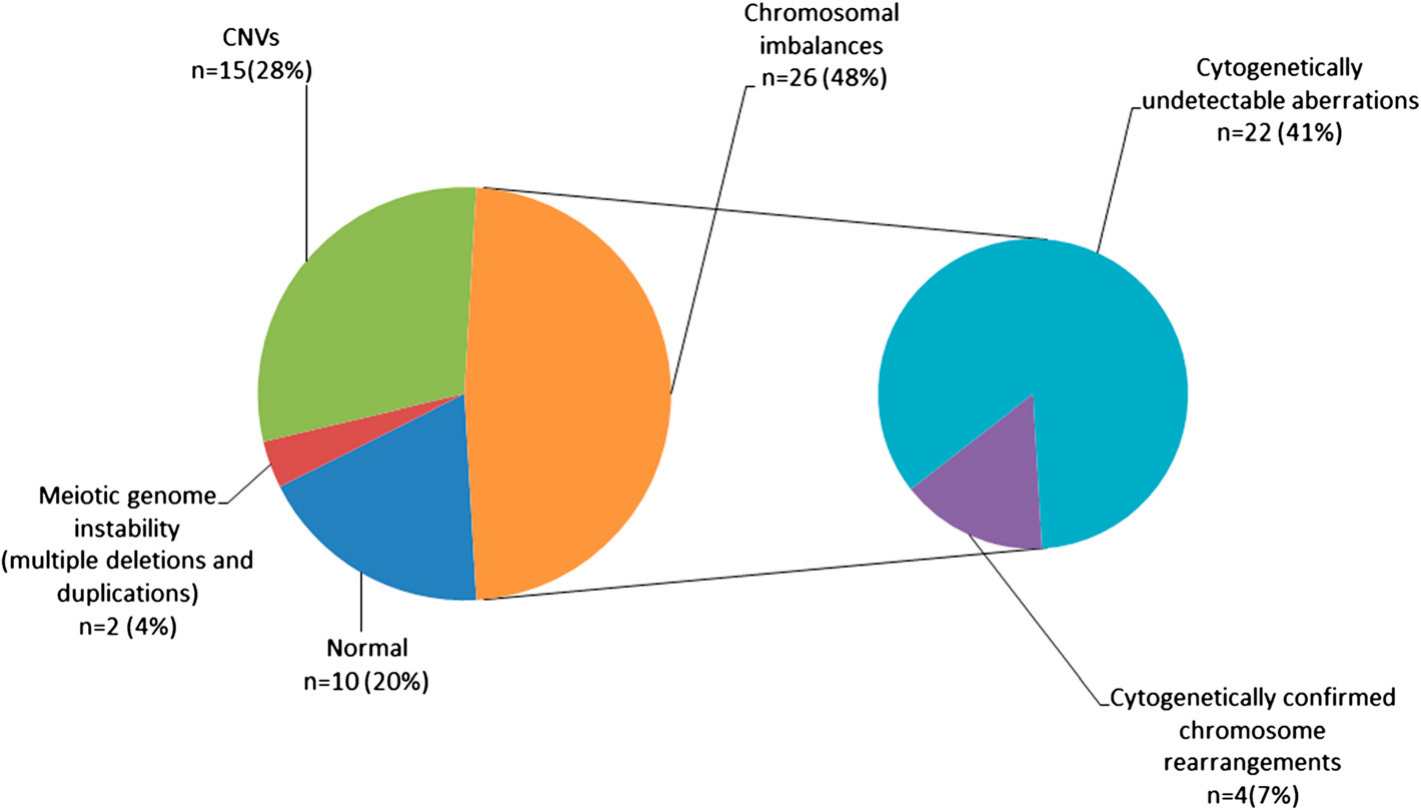
**The incidence of chromosome imbalances (subdivided to cytogenetically detectable and undetectable abnormalities), CNVs and meiotic genome instability in the Russian cohort of children with intellectual disability with or without autism and congenital anomalies.** Cytogenetically detectable cases were patients, who were cytogenetically re-evaluated. This has yielded the correct diagnosis (see also Table 1).

Fifty four members of the Russian cohort of children with intellectual disability, autism, epilepsy and congenital anomalies were studied by BAC array CGH with the resolution of 0.3-1 Mb. Some examples of array CGH graphical overviews are shown in Figure 1. Details of the cohort (array CGH results and patients) are summarized in Table 1. Figure 2 overviews the incidence of genomic imbalances in the cohort according to their type and to the diagnosing feasibility or, in other words, the difference between cytogenetically visible (patients re-evaluated by cytogenetic analysis after array CGH) and cytogenetically invisible chromosome aberrations. CNVs were evaluated by a bioinformatic approach towards the CNV/gene prioritization followed by genotype-phenotype correlations. Selected cases were confirmed by molecular cytogenetic and molecular genetic methods (Table 1).

**Table 1:** The Russian cohort of children with intellectual disability with or without autism and congenital anomalies: clinical information and array CGH results

**Case #**	**Age**	**Clinical features**	**Array CGH results (according to ISCN (2013) [**[11]**])**
1^*^	10 m (months)	Partially similar to the phenotype of 1p36 deletion and Pallister–Killian syndromes (“reverse phenotyping”, as defined by Slavotinek, 2008 [12])	47,XXY.arr(X)*x*2,1p36.32p36.22(4,932,799-9,373,344)x1,12p13.33p11.1(153,050-33,636,183)*x*2~4,
2^*^	1 y (year)	Developmental delay, pachygyria, multicystic, encephalomalacia seizures, obesity, short neck, and ear dysmorphism	arr 19q13.12p13.2(41,730,447-43,880,848)x1
3^*^	7 y	Mild intellectual disability and facial dysmorphisms	arr 20q11.21(29,392,835-32,017,043)x1
4^*^	8 y	Developmental delay, intellectual disability-microcephaly, single transverse palmar crease, acrosyndactyly, microphthalmia, optic nerve atrophy, downslanting palpebral fissures	46,XX,r(11)(p15.5q24.1).arr 9q21.33(90,067,781-90,185,591)x3,11q24.1q25(121,411,392-134,916,587)x1,18p11.31(3,919,884-4,092,260)x1
5	4 y 7 m	Developmental delay, intellectual disability, microcephaly, single transverse palmar crease, clinodactyly, hypotelorism, short neck, short stature, broad breast	arr(1–22,X)*x*2
6^*^	2 y	Severe developmental delay, partially similar to the Wolf-Hirschhorn syndrome phenotype (“reverse phenotyping”, as defined by Slavotinek, 2008 [12])	arr 4p16.3p16.1(131,000-8,695,702)×1,8p23.3p23.1(304,177-6,396,170)×3,14q32.33(106,140,000-106,230,000)×1,17p12(14,080,569-15,022,225)×1
7	4 y 3 m	Intellectual disability, high-arched palate, low-set ears, facial dysmorphisms	arr(1–22)х2,(XY)x1
8^§^	10 y	Intellectual disability, developmental delay, facial dysmorphisms, single transverse palmar crease, pectus excavatum, seizures, short neck	arr Xp22.33p22.2(2,333,897-9,726,574)x1,3p26.3p22.3(200,000-36,550,871)x3,12q13.13(50,803,959-50,962,257)x3,17p11.2(18,863,800-19,021,902)x3
9	1y 8 m	Severe developmental delay, seizures, microcephaly, broad flat face, myopia	arr(1–22,X)*x*2
10	4y 4 m	Mild cognitive delay, optic nerve hypoplasia, strabismus, upslanting palpebral fissures, epicanthal fold, hypertrichosis, hypertelorism, broad nasal bridge	arr(1–22)х2,(XY)x1
11	4 y 3 m	Mild intellectual disability and speech delay, autism, syndactyly	46,XY.arr 9q34.2q34.3(134,270,751-135,710,693)x1,16p13.3(755,100-1,094,049)x1,21q22.3(43,556,393-45,308,985)x1
12^*^	9 y	Autistic disorder (Asperger syndrome), cervical spine abnormalities, pancreatitis	46,XY,arr 3p22.1p21.32(42,284,371-44,741,252)x1
13	4 y 7 m	Speech and cognitive delay, seizures, macrocephaly, congenital heart defect, optic nerve atrophy, facial dysmorphisms	46,XY,9phqh.arr Xp11.23(47,651,854-47,861,472)*x*2,16p13.3(596,000-776,000)x3
14	4 y	Developmental and speech delay, ataxia	arr(1–22)х2,(XY)x1
15^§^	3 m	Developmental and cognitive delay, corpus callosum agenesis, congenital heart defect, microcephaly, pulmonary hypoplasia, facial dysmorphisms	arr 10q25.2qter(112,060,103-135,168,517)x3,13q33.3qter(108,359,829-114,080,000)x1
16	6 y 2 m	Mild developmental and cognitive delay, autistic features, microcephaly, hypertelorism, clinodactyly, syndactyly, small ears	arr(1–22)х2,(XY)x1
17	4 y11 m	Intellectual disability, speech delay, microcephaly, hypertelorism, syndactyly	46,XX,1phqh,1qh+.arr 13q32.2(98,510,084-98,669,155)x1,16p13.3(2,141,102-2,327,412)x3
18	7 m	Developmental delay, intellectual disability-intrauterine growth retardation, cryptorchidism, facial dysmorphisms, camptodactyly, apnea	46,XY.arr 19p13.3(260,000-4,953,188)x3
19	2 y 7 m	Intellectual disability, speech delay, autistic features, kidneys malformation	arr 5q35.3(176,800,642-176,968,043)х3,12q24.11(111,011,590-111,227,098)х3,16q12.1(47,323,368-47,469,918)х1
20	2 y 4 m	Mild developmental and cognitive delay, syndactyly, facial dysmorphisms	46,XY.arr 10q26.2q26.3(128,192,760-134,070,099)x1,17q12(34,568,087-34,634,375)x1
21	5 y	Intellectual disability, speech delay, cognitive delay, autism	arr Yq11.223(26,903,388-27,059,018)х2,5q13.2(70,231,675-70,389,712)х3
22	1 y 11m	Severe developmental delay and congenital heart disease (long QT syndrome)	46,XY,9phqh,22ps+.arr 1p36.33(1,893,455-2,280,666)x3,7p22.3(1,112,781-1,975,820)x3,16p13.3(510,000-1,154,048)x3,17p13.3(863,994-1,061,378)x3,20q13.33(60,810,732-62,629,910)x3,22q13.33(49,932,121-50,526,990)х3
23^*,ǂ^	4 y	Intellectual disability, developmental delay, facial dysmorphisms, high-arched palate, congenital dislocation of the hip	46,XY,1phqh.arr Xq28(153,130,000-153,647,227)х2,14q32.2(101,142,170-101,318,256)x3,16p13.3(814,190-962,809)х3,22q13.1(38,003,877-38,022,161)х3
24	1 y 9 m	Developmental delay, optic nerve hypoplasia	46,XY.arr 16p13.3(3,592,260-3,783,073)х3
25	6 y	Intellectual disability, developmental and speech delay, microcephaly, seizure, partial optic nerve atrophy, ataxia, muscular hypotonia	arr(1–22,X)*x*2
26	4 y	Intellectual disability, speech delay, developmental delay, microcephaly, epicanthic fold, broad nasal bridge, hypertelorism, syndactyly, clinodactyly, protruding ears	46,XX.arr 9q34.2q34.3(135,531,562-138,074,496)x1,14q13.1(33,923,538-34,022,136)x1, 17p11.2(18,200,082-18,378,155)x1,21q22.3(45,124,515-45,308,212)x1
27^*^	4 y 5 m	Lumbosacral dysgenesis, microcephaly, hypospadias, congenital ventricular septal defect, pectus excavatum, myelocele, small lower jaw, upslanting palpebral fissures, teeth anomalies, autistic features, mild cognitive delay	46,XY,1qh-.arr 7q36.2q36.3(152,768,630-158,261,821)x1
28	2 y 2 m	Intellectual disability, severe developmental and cognitive delay, microcephaly, seizures, hypertelorism, single transverse palmar crease, syndactyly, congenital heart defect, Hirschsprung disease	46,XY,9phqh,9qh-.arr 7p11.2(55,431,539-55,596,905)x3,9q32(115,760,113-115,953,658)x3,11p15.5(2,261,568-2,430,797)x3, 17q21.31(41,559,197-41,733,821)x3
29	1 y 5 m	Developmental delay, intellectual disability, downslanting palpebral fissures, hypertelorism, facial dysmorphisms	arr(1–22,X)*x*2
30^ǂ^	2 y 11 m	Mild intellectual disability and speech delay, obesity, hypogenitalism	arr Yq11.223(24,820,670-27,059,018)x0,3q22.3(136,509,072-136,662,243)x1,4p16.3(762,790-916,783)x1
31	2 y 5m	Intellectual disability and speech delay, short stature, flat nasal bridge, muscle hypotonia, MRI abnormalities	46,XX,15phqh.arr 1p36.32(2,368,110-2,522,845)x1,4p16.3(752,795-906,771)x1,20q13.33(60,355,527-60,494,531)x1
32^ǂ^	9 y10 m	Intellectual disability, autism, multiple hematomas, teeth anomalies	46,XX,1phqh,9phqh.arr Xq13.1(70,474,715-70,608,113)x1,Xq22.1(100,082,182-100,164,698)x1,Xq28(153,145,800-153,301,421)x1,14q12(23,575,187-23,732,133)x1
33	4 y	Speech and cognitive delay, somatomegaly, congenital heart defect	arr (1–22)х2,(XY)x1
34	7 y	Speech delay, syndactyly, deafness, sandal gap, high-arched palate, congenital heart defect, epiphyseal dysplasia	46,XY,1phqh.arr 9q34.3(137,679,970-137,867,305)x3
35^ǂ^	11 y	Speech and cognitive delay, seizures, autism, facial dysmorphisms	46,XX.arr Xq25(129,171,486-129,265,190)x1,Xq28(153,145,800-153,301,421)x1
36	1y 10 m	Developmental and speech delay, trigonocephaly, seizures, craniostenosis, facial dysmorphisms	arr (1–22)х2,(XY)x1
37	2y 6m	Intellectual disability, autistic features, facial dysmorphisms, single transverse palmar crease	arr (1–22)х2,(XY)x1
38^ǂ^	4 y 6 m	Speech delay, cognitive delay, clinodactyly, hypertelorism	46,XY.arr Xq12(66,858,503-67,027,800)x0
39	3 y	Developmental delay, intellectual disability, hydrocephaly, seizures, facial dysmorphisms	46,XX,16qh-,16qh-.arr Xp22.2(10,353,886-10,523,886)x1,13q14.3(48,903,923-49,068,912)x1
40	9 m	Developmental delay, intellectual disability, congenital heart defect, myopia, facial dysmorphisms	46,XX,1qh-,13pstk+.arr 5q13.2(68,931,140-72,690,180)x3
41	3 y	Severe speech and cognitive delay, microcephaly, facial dysmorphisms, single transverse palmar crease	46,XX,9qh-.arr Xq13.3(74,566,312-74,732,745)x1,Xq21.1(33,000,232-33,118,926)x1, 12q24.31(124,056,061-124,234,471)x1, 20q13.13(48,091,851-48,205,439)x1
42	7 y	Speech and cognitive delay, high-arched palate, single transverse palmar crease, small teeth	46,XX,15phqh+.arr 1p36.32(2,368,110-3,076,708)x1,5p15.33(403,337-1,562,887)x1
43	4 y	Intellectual disability, speech and cognitive delay, facial dysmorphisms	46,XY,16qh-.arr 9q34.2(135,531,566-136,387,456)x1,21q22.3(44,958,870-45,311,763)x1
44	4 y	Intellectual disability, speech delay, neurobehavioral disorder	arr 11q23.3(120,091,054-120,251,056)x1,16q21(59,719,829-59,928,048)x3
45	6 y	Severe speech and cognitive delay, autism, neuromuscular disorder	46,ХY,14cenh+ps+.arr Xq13.1(68,969,384-69,105,568)*x*2,17q21.31(41,559,185-41,734,030)x3
46	2 y	Intellectual disability, speech and cognitive delay, microcephaly, autistic features, syndactyly	46,XX,1qh-.arr Xp22.31(5,981,359-6,146,376)x1,2q36.1(222,366,094-222,493,489)x3,10q26.3(135,070,014-135,240,498)x3
47	1 y	Developmental and speech delay, congenital heart defect, congenital lung malformation, syndactyly, esophageal atresia, cleft palate	46,XY.arr 7q11.23(76,142,331-76,323,858)x3,17q21.31(41,559,185-41,734,024)x3
48^ǂ^	4 y	Intellectual disability, autism	46,XY, 9phqh.arr Xq28(154,487,912-154,657,923)x0
49^ǂ^	4 y10 m	Intellectual disability, developmental delay, facial dysmorphisms, clinodactyly, dentinogenesis imperfecta, enteroparesis	46,ХX,9phqh,17ps.arr Xp11.22(53,447,485-53,580,290)x1,Xq28(153,108,683-153,301,517)x1,6q27(167,301,688-167,434,477)x3,13q12.13(24,807,453-24,811,446)x3,
50	4 y	Intellectual disability, developmental delay, autism, Rett syndrome-like phenotype	46,ХX.arr Xp11.22(53,447,485-53,580,289)х1,Xq21.1(76,803,206-77,014,852)x1,Xq24(118,784,619-119,190,484)x1,Xq25(128,518,217-128,669,369)x1,Xq28(153,435,103-153,876,315)x1,11p15.5(2,624,666-2,805,697)x1,17p11.2(18,863,800-19,021,902)x1.
51^ǂ^	1 y 10 m	Developmental and speech delay, facial dysmorphisms	46,ХX.arr Xq28(153,435,103-153,609,374)х1,Xq28(152,731,931-152,937,571)x1,14q32.33(105,149,438-105,332,624)x1
52^ǂ^	4 y 6 m	Intellectual disability, developmental delay, autism	46,XY,9phqh.arr Yq11.223(23,230,058-25,468,406)x0,1p36.33(2,120,746-2,270,566)x1,5q13.2(68,931,140-70,516,922)x1
53*^§^	6 y	Developmental and speech delay, microcephaly, cleft palate, facial dysmorphisms	arr Xp22.31(7,093,720-7,192,728)x1,Xq12(67,322,691-67,466,465)х1,Xq22.3(109,649,422-109,735,628)x1,1q44(246,424,879-249,250,621)x1,16p13.3p13.12(86,162-14,529,445)x3,6q26(163,806,110-163,923,922)x1
54	5 y 10 m	Intellectual disability, developmental delay, autism, Rett syndrome-like phenotype	46,ХX,1phqh.arr Xp22.12(19,630,934-19,799,405)х1,6p12.3(133,786,272-133,963,451)x3

* — confirmed by molecular cytogenetic methods (i.e. FISH); ^ǂ^ — confirmed by molecular genetic methods (PCR or QPCR);

^§^ — conventional karyotyping was performed after array CGH analysis;

### Chromosomal imbalances

Constitutive chromosomal imbalances were detected in 26 individuals (including case #1 exhibiting three presumably unrelated chromosome abnormalities). Chromosomal imbalances (including multiple chromosome abnormalities) observed in cases #1, #8, #11, #11, #12, #18, #26, #40, and #43 were found to be unique to this cohort (Table 1). Among them, we found a recurrent chromosome abnormality specific to the Russian cohort of children with intellectual disability, autism, epilepsy and congenital anomalies. This was referred to co-occurrence of chromosome 9 long arm deletion in 9q34.2q34.3 and chromosome 21 long arm deletion in 21q22.3 (cases #11, #26, #43; Table 1). One can suggest that a ~179 kbp interval on 9q34.2 and a ~187 kbp interval on 21q22.3 (deduced on the basis of breakpoint locations) are both specifically organized at the sequence level to produce a complex genomic rearrangement causing intellectual disability with autistic features, speech delay and facial dismorphisms.

### CNVs

Cases #22 and #28 exhibited multiple CNVs (more than three submicroscopic duplications). All the duplications have been defined to contribute to the severe phenotypic outcome by bioinformatic analysis. Since similar cases were not found in the available literature, we have taken opportunity to designate the condition as meiotic (constitutional) genomic instability.

By bioinformatics, we have identified clinically relevant CNVs in 15 individuals. These were found to encompass genes, mutations in which cause developmental delays, congenital anomalies, intellectual disability, autism, epilepsy, or other neuropsychiatric disorders. Alternatively, CNV genes, which have not as yet been associated with a particular disease phenotype, were not necessarily those associated with benign genome variations. The bioinformatic analysis has demonstrated a significant proportion of such genes to be involved in functional pathways, which, if altered, can be disease-causing (see Additional file 1).

### Comparative cohort characterization

To make a comparative characterization of the cohort, we have addressed it in a case-by-case manner. Previously unreported CNVs, exclusive chromosomal imbalances and meiotic genome instability were excluded. Case # 1 is the unique combination of known chromosomal abnormalities [13,14]. Multiple chromosome abnormalities are extremely rare and are usually associated with severe phenotypes (i.e. prenatal mortality) [15,16]. Severe congenital anomalies were eventually observed in this case.

Case #2 is a microdeletion of the chromosome 19 long arm that is clinically similar to 19q13.11 deletion syndrome [17]. Case #3 is a chromosome 20 long arm microdeletion associated with mild intellectual disability and facial dysmorphisms. The chromosomal region was involved in larger chromosome 20 long arm deletions, which demonstrated more severe phenotypes [18].

Ring chromosome 11 (case #4) characterized in the present study is phenotypically similar to previous cases [19], exhibiting, however, additional clinical features (autism), which are attributed to the presence of CNVs. Case #6 has turned to be a recurrent translocation between chromosomes 4 and 8 causing the Wolf-Hirschhorn syndrome, being, however, more complex as those described previously [20].

Case #8 was a chromosome imbalance identified in a distant patient (immediate cytogenetic analysis was not available). After array CGH, additional further molecular cytogenetic studies and cytogenetic re-evaluation, it was defined as an unbalanced translocation between short arms of chromosomes X and 3. Several similar cases have been described [21,22].

Case #12 is a deletion of chromosome 3 short arm in a child with Asperger syndrome. Chromosomal abnormalities are occasional in this neurobehavioral disorder. However, a study has shown positive Asperger syndrome linkage to 3p21-24 [23].

In case #13, a CNV (duplication) in Xp11.23 was found. Recently, a duplication of the same chromosomal region was shown to cause similar phenotype [24]. Case #15 was another chromosome imbalance identified in a distant patient that has been defined as an unbalanced translocation between long arms of chromosomes 10 and 13 after cytogenetic re-evaluation.

Chromosomal rearrangements in the chromosome 19 short arm are extremely rare [25]. Case #18 is the largest ever reported 19p subtelomeric duplication. Case #20 is a 10q subtelomeric deletion; similar cases were previously described, but the clinical manifestations are usually variable and a specific phenotype is not associated [2]. Case #23 exhibits a number of clinical features of the Xq28 duplication syndrome [26]; the phenotypic discrepancies have been attributed to CNVs.

A 7q subtelomeric deletion was identified in case #27. Despite of characteristic clinical features (lumbosacral dysgenesis) [27], it was almost impossible to suggest this chromosomal imbalance prior to array CGH. Cases #30 and #52 have demonstrated chromosome Y long arm deletions. Similar cases were detected during a case–control array CGH study applied to prenatal diagnosis, but the outcome has remained uncertain [28].

Cases #32, #32, #49 and #50 were found to exhibit Xq28 deletions. A retrospective clinical analysis has shown that Rett-syndrome-like phenotypic manifestations do present in these girls. It is not surprising inasmuch as two cases of these deletions involved *MECP2*. Interestingly, there have been several attempts to characterize chromosomal rearrangements by molecular cytognetic techniques in Rett syndrome cohorts without apparent evidence for the presence of genomic variations involving *MECP2*[29-31].

In case #39, a CNV within *RB1* gene was found and the phenotype was similar to interstitial 13q microdeletions [32]. An Xq28 CNV (involving *RAB39B* gene) was found in a child with intellectual disability and autism (case #48). It is to note, that *RAB39B* mutations cause X-linked mental retardation associated with autism, epilepsy, and macrocephaly [33].

Finally, case #53 was the third chromosome imbalance identified in a distant patient. Subsequently, it turned out to be an unbalanced translocation between the long arm of chromosome 1and the short arm of chromosome 16 after fluorescence in situ hybridization (FISH) and cytogenetic evaluation. Comparing the phenotype with previous reports [34,35], we concluded that the clinical features in this case has similarities as with trisomy 16p as with 1q subtelomeric deletions.

## Discussion

The present study has again provided evidence that array CGH is a powerful technique for uncovering chromosomal imbalances and genomic rearrangements. Previous case–control studies (reviewed in [2,5,8-10]) have demonstrated the high diagnostic yield of microarray-based whole-genome analysis and the validity of array-CGH-based technologies for detecting genomic imbalances in clinical populations. However, the detection rate (over 50%) seems to be significantly higher in our study comparing to the previous ones (5-20%) [1-10]. Since the incidence of genomic rearrangements detectable by array CGH is strongly influenced by selection bias of patients, the reported detection rate is likely to be higher inasmuch as our criteria were much more discriminating than previously applied (i.e. evaluating the diagnostic yield in “idiopathic” cohorts). Thus, we do not insist that the reported detection rate is characteristic for an array CGH control cohort study. Still, it can be expected that an application of array CGH to highly selected patients is able to demonstrate an impressive detection rate of structural genome variations.

It is generally recognized that numerical and structural chromosome abnormalities are the major genetic causes of postnatal morbidity including a wide spectrum of diseases associated with brain dysfunction [13,14]. However, before the implementation of molecular cytogenetic techniques (array-CGH- and FISH-based technologies), the contribution of genome/chromosome imbalances to the etiology was usually considered as less than 5% [4,7,9,10,14]. Array CGH data are not only relevant to molecular diagnosis, but also to the discovery of genetic (genomic) mechanisms of brain diseases [1-10,14]. Here, we were also able to demonstrate similar etiologic yield. In this instance, the present gene list (Additional file 1) possesses implications for the delineation of the cellular and molecular basis of disease and requires further bioinformatic analyses.

Although array CGH is a highly efficient technique for definition of chromosome/genome rearrangements in clinical populations, it has limitations. For example, it does not properly reflect somatic mosaicism [36-38], which is commonly associated with postnatal morbidity, including brain diseases [37-41]. The pathogenic value of submicroscopic genomic variations can be a matter of conjecture [2-10]. Therefore, array-CGH-based methods have to be combined with molecular cytogenetic, post-genomic and bioinformatic technologies for detailed studies of disease mechanisms. Nonetheless, array CGH assays are mandatory for such studies.

Apart from numerical constitutional and mosaic chromosome abnormalities (case #1) the genomic rearrangements were found to affect 1247 genes in total. Details about these genes are provided in Additional file 1. Accordingly, we speculate that the majority of these genes can be mutated in intellectual disability, autism or epilepsy. The latter can be easily seen if one addresses a molecular/clinical genetic database (i.e. OMIM — http://www.omim.org/; referenced in Additional file 1). Moreover, combinations of deletions/duplications affecting these genomic loci produce a “CNV burden”, which can be considered a possible genetic cause of postnatal morbidity. On the other hand, it is to recognize that a significantly lesser part of them are true candidates for the aforementioned neuropsychiatric diseases.

Array CGH does improve the etiologic yield across the spectrum of patients with neuropsychiatric disorders and congenital anomalies. This improvement has been achieved by systematic cohort studies. The available literature indicates that we report on the first comprehensive array CGH analysis of a Russian cohort of children with intellectual disability, autism, epilepsy and congenital anomalies. The efficiency of the approach (array CGH + bioinformatic strategy) allows us to expand the cohort. To this end, it is necessary again to point out that patient pre-selection provided for the discovery of up to 31% of previously unreported genomic rearrangements among detectable imbalances. In other words, this report describes novel disease genes uncovered in a relatively small cohort. Hence, our next study of the expanded cohort would certainly lead to discovering new candidate genes and shared molecular pathways of intellectual disability, autism, epilepsy and related neuropsychiatric disorders.

## Methods

### Patients

From November 2007 to May 2012, 2426 patients referred to our molecular cytogenetic facilities. All the patients were studied by conventional karyotyping (G- and C-banding analyses) at a G-banding resolution of about 550 bands according to ISCN (2013) [11]. The ages varied between 1 month and 18 years. Fifty four patients were highly selected to be the first participants of the Russian cohort of children with intellectual disability, autism, epilepsy and congenital anomalies based on clinical and cytogenetic/molecular cytogenetic data. Molecular cytogenetic techniques (FISH-based methods) were used to exclude cases of cytogenetically visible chromosome abnormalities (confirmation by molecular cytogenetic techniques) and microdeletion/microduplication syndromes. FISH was also used in cases of chromosomal mosaicism (especially, those children who were the participants of the Russian autism cohort and were shown to exhibit chromosomal mosaicism; for more details see [42-44]). Such cases have been excluded. Subtelomeric chromosomal rearrangements (commonly detected in children with intellectual disability [2]), addressed by a previously reported FISH analysis with original DNA probes [45], were excluded from the cohort, as well. Cases positive for mutations causing fragile X and Rett syndrome (common causes of intellectual disability and autistic features in children) addressed by molecular genetic techniques (PCR/QPCR and direct sequencing of the *MECP2* gene) were ruled out. For three distant patients, immediate cytogenetic analysis was not available due to natural (distance) limitations. As a result, they were included in the cohort. Two cytogenetically positive cases (supernumerary chromosome X in a male child and ring chromosome 11) exhibiting extremely atypical phenotypic manifestations were also included. Written informed consent was obtained from the patients’ parents.

### Array CGH

Array CGH was performed with the customized human genomic microarrays (slightly modified Constitutional Chip®4.0) containing about 5200 human BAC clones (Human BAC Array-System, Perkin Elmer, USA). The resolution of the whole genome scan was estimated to be 0.3-1 Mb. The microarrays contain more than 1200 BAC probes for the majority of "new" and "old" microdeletion/microduplication syndromes (for the description see [2,3,5,7]), about 900 BAC probes — subtelomeric regions, about 100 BAC probes — percintromeric chromosomal regions, 621 BAC probes — chromosome X, and about 2000 BAC probes — remaining euchromatic chromosomal regions, allowing the whole genome to be scanned with a resolution of at least 1 Mb. DNA labelling, hybridization, detection and data analysis was made according to previously described protocols [46] and to manufacturers’ instructions.

### Confirmation methods

Molecular cytogenetic (FISH) and molecular genetic (PCR/QPCR) methods were used for the confirmation of genomic rearrangements in a number of cases (n=18). FISH with DNA probes from our original collection (probes for heterochromatic and euchromatic (repetitive and unique) chromosomal regions) was performed as described previously [45,47,48]. The confirmation was performed in nine cases (Table 1).

PCR (or QPCR) was essentially used to confirm the rearrangements affecting chromosomes X and Y (i.e. deletions of *AR* in Xq12, *MECP2* in Xq28, and DAZ loci in Yq11.223). The confirmation was performed in nine cases (Table 1).

### Bioinformatics

*In silico* (bioinformatic) analyses were performed in part according to Iourov et al., 2009, 2010 [49,50]. To determine the pathogenic value of CNVs, genotype-phenotype correlations and CNV or gene prioritization were done by a series of evaluations using clinical/cytogenetic and genomic variation databases as well as bioinformatic tools for genome, epigenome and pathway analysis.

Genotype-phenotype correlations and pathogenic value of CNVs were estimated using DECIPHER (database of unbalanced chromosome aberrations) — http://decipher.sanger.ac.uk/, OMIM (online Mendelian inheritance in Man) — http://www.omim.org/, The Phenotype-Genotype Integrator (PheGenI) — http://www.ncbi.nlm.nih.gov/gap/PheGenI, SFARI Gene/AutDB (web-based searchable database for autism research) — http://www.mindspec.org/autdb.html, and a Catalog of Published Genome-Wide Association Studies (NHGRI) — http://www.genome.gov/gwastudies/.

CNVs were also compared to the Database of Genomic Variants (http://dgvbeta.tcag.ca/dgv/app/home?ref=GRCh37/hg19). The prioritization of CNVs and genes was done using BioGPS (a gene annotation and expression) — http://biogps.org, Ensembl Genome Browser — http://www.ensembl.org/index.html, KEGG (Kyoto Encyclopedia of Genes and Genomes) — http://www.genome.jp/kegg/, NCBI BioSystems Database — http://www.ncbi.nlm.nih.gov/biosystems, NCBI Gene — http://www.ncbi.nlm.nih.gov/gene/, NCBI Build 37.1/NCBI Map Viewer — http://www.ncbi.nlm.nih.gov/projects/mapview/map_search.cgi?taxid=9606, REACTOME — http://www.reactome.org/*,* Pathway Commons — http://www.pathwaycommons.org/pc/, and UCSC Genome Browser — http://genome.ucsc.edu/.

## Endnote

^a^The data reported in the present article have been presented in part at the European Human Genetics Conference 2011, Amsterdam, The Netherlands, May 28–31, 2011.

## Abbreviations

BAC: bacterial artificial chromosome; CNVs: copy number variations; CGH: comparative genomic hybridization; FISH: fluorescence in situ hybridization; PCR: polymerase chain reaction; QPCR: quantitative polymerase chain reaction.

## Competing interests

The authors declare that they have no competing interests.

## Authors’ contributions

IYI, SGV and YBY conceived the research, designed the study, and wrote the manuscript. SGV and YBY referred the patients for the study. IYI, OSK and APS performed the array CGH analysis. IYI and MAZ performed the bioinformatic analysis and prepared the supplemental material (Additional file 1). IYI and OSK performed molecular cytogenetic and molecular genetic analyses. All authors have read and approved the final manuscript.

## Supplementary Material

Additional file 1 Genes affected by chromosome imbalances and CNVs. Click here for file
