# Supplementary material for: Molecular karyotyping by array CGH in a Russian cohort of children with intellectual disability, autism, epilepsy and congenital anomalies
Source: Mol Cytogenet. 2012 Dec 31;5:46. doi: 10.1186/1755-8166-5-46 (PMC3547809; doi:10.1186/1755-8166-5-46)
Supplement: Additional file 1 — Genes affected by chromosome imbalances and CNVs. [file 1755-8166-5-46-S1.pdf]

Genes affected by chromosome imbalances and CNVs

(numerical constitutional and mosaic chromosome abnormalities excluded)

(RED — deleted genes; BLUE — duplicated genes)

Patient #	Loci	Aberration type	Gene	OMIM, #	Function	Disease
**1**	**1p36.32p36.22**	**deletion**	NPHP4	607 215	This gene encodes a protein involved in renal tubular development and function.	Nephronophthisis type 4, a renal disease, Senior-Loken syndrome type 4
			KCNAB2	601 142	Voltage-gated potassium (Kv) channels represent the most complex class of voltage-gated ion channels from both functional and structural standpoints. Their diverse functions include regulating neurotransmitter release, heart rate, insulin secretion, neuronal excitability, epithelial electrolyte transport, smooth muscle contraction, and cell volume.	
			CHD5	610 771	This gene encodes a neuron-specific	

					protein that may function in chromatin remodeling and gene transcription. This gene is a potential tumor suppressor gene that may play a role in the development of neuroblastoma.	
			RPL22	180 474	This gene encodes a cytoplasmic ribosomal protein that is a component of the 60S subunit. The protein belongs to the L22E family of ribosomal proteins. Its initiating methionine residue is post-translationally removed. The protein can bind specifically to Epstein-Barr virus-encoded RNAs (EBERs) 1 and 2.	
			ICMT	605 851	This gene encodes the third of three enzymes that posttranslationally modify isoprenylated C-terminal cysteine residues in certain proteins and target those proteins to the cell membrane. This enzyme localizes to	

					the endoplasmic reticulum.	
			HES3	609 971	This deduced 186-amino acid protein contains an N-terminal basic helix-loop-helix domain, followed by an orange domain, a proline-rich domain, and a C-terminal WRPW motif.	
			ACOT7	602 587	This gene encodes a member of the acyl coenzyme family. The encoded protein hydrolyzes the CoA thioester of palmitoyl-CoA and other long-chain fatty acids. Decreased expression of this gene may be associated with mesial temporal lobe epilepsy.	
			HES2	609 970	This deduced 173-amino acid protein contains an N-terminal basic helix-loop-helix domain, followed by an orange domain, a proline-rich domain, and a C-terminal WRPW motif. HES2 ESTs were found in a placenta cDNA library and in several	

					cancer cDNA libraries.	
			ESPN	606 351	This gene encodes a multifunctional actin-bundling protein.<br>It plays a major role in regulating the organization, dimensions, dynamics, and signaling capacities of the actin filament-rich, microvillus-type specializations that mediate sensory transduction in various mechanosensory and chemosensory cells.	Deafness, autosomal recessive 36; deafness, neurosensory, without vestibular involvement, autosomal dominant
			TNFRSF25	603 366	This receptor has been shown to stimulate NF-kappa B activity and regulate cell apoptosis. The signal transduction of this receptor is mediated by various death domain containing adaptor proteins.	
			PLEKHG5	611 101	This gene encodes a protein that activates the nuclear factor kappa B (NFKB1) signaling pathway.	Spinal muscular atrophy, distal, autosomal recessive, 4
			TAS1R1	606 225	Multiple transcript variants encoding	

					different isoforms have been found for this gene.	
			ZBTB48	165 270	zinc finger and BTB domain containing 48	
			THAP3	612 532	THAP domain containing, apoptosis associated protein 3	
			CAMTA1	611 501	Calmodulin binding transcription activator 1	
			VAMP3	603 657	The protein encoded by this gene was shown to be the human equivalent of the rodent cellubrevin. In platelets the protein resides on a compartment that is not mobilized to the plasma membrane on calcium or thrombin stimulation.	
			PER3	603 427	This gene is a member of the Period family of genes and is expressed in a circadian pattern in the suprachiasmatic nucleus, the primary circadian pacemaker in the mammalian brain.	

					Genes in this family encode components of the circadian rhythms of locomotor activity, metabolism, and behavior. Circadian expression in the suprachiasmatic nucleus continues in constant darkness, and a shift in the light/dark cycle evokes a proportional shift of gene expression in the suprachiasmatic nucleus. The specific function of this gene is not yet known.	
			UTS2	604 097	The protein encoded by this gene is a member of the TNF-receptor superfamily. This receptor contributes to the clonal expansion, survival, and development of T cells. It can also induce proliferation in peripheral monocytes, enhance T cell apoptosis induced by TCR/CD3 triggered activation, and regulate CD28 co-stimulation to	

					promote Th1 cell responses.	
			TNFRSF9	602 250	The product of this gene belongs to the peptidase C56 family of proteins. It acts as a positive regulator of androgen receptor-dependent transcription. It may also function as a redox-sensitive chaperone, as a sensor for oxidative stress, and it apparently protects neurons against oxidative stress and cell death.	
			PARK7	602 533	The product of this gene belongs to the peptidase C56 family of proteins. It acts as a positive regulator of androgen receptor-dependent transcription. It may also function as a redox-sensitive chaperone, as a sensor for oxidative stress, and it apparently protects neurons against oxidative stress and cell death.	Amyotrophic lateral sclerosis-Parkinsonism/dementia complex 2 Parkinson disease 7, autosomal recessive early-onset
			ERRFI1	608 069	ERRFI1 is a cytoplasmic protein whose expression is upregulated with	

					cell growth. It shares significant homology with the protein product of rat gene-33,<br>which is induced during cell stress and mediates cell signaling	
			SLC45A1	605 763	unknown	
			RERE	605 226	This gene encodes a member of the atrophin family of arginine-glutamic acid (RE) dipeptide repeat-containing proteins. The encoded protein co-localizes with a transcription factor in the nucleus, and its overexpression triggers apoptosis.	
			ENO1	172 430	This gene encodes alpha-enolase, one of three enolase isoenzymes found in mammals.	Enolase deficiency
			CA6	114 780	The protein encoded by this gene is one of several isozymes of carbonic anhydrase. This protein is found only in salivary glands and saliva and protein may play a role in the	

					reversible hydratation of carbon dioxide though its function in saliva is unknown.	
			SLC2A7	610 371	SLC2A7 belongs to a family of transporters that catalyze the uptake of sugars through facilitated diffusion. This family of transporters shows conservation of 12 transmembrane helices as well as functionally significant amino acid residues	
			SLC2A5	138 230	Solute carrier family 2 (facilitated glucose/fructose transporter), member 5	
			MIR34A	611 172	microRNA 34a	
			H6PD	138 090	hexose-6-phosphate dehydrogenase (glucose 1-dehydrogenase)	Cortisone reductase deficiency
			SPSB1	611 657	splA/ryanodine receptor domain and SOCS box containing 1	

**2**	**19p13.12p13.2**	**deletion**				
			AXL	109 135	The protein encoded by this gene is a member of the receptor tyrosine kinase subfamily.<br>Although it is similar to other receptor tyrosine kinases, this protein represents a unique structure of the extracellular region that juxtaposes IgL and FNIII repeats. It transduces signals from the extracellular matrix into the cytoplasm by binding growth factors like vitamin K-dependent protein growth-arrest-specific gene 6. It is involved in the stimulation of cell proliferation and can also mediate cell aggregation by homophilic binding.	
			HNRNPUL1	605 800	This gene encodes a nuclear RNA-binding protein of the heterogeneous nuclear ribonucleoprotein (hnRNP) family.<br>This protein binds specifically to	

					adenovirus E1B-55kDa oncoprotein. It may play an important role in nucleocytoplasmic RNA transport, and its function is modulated by E1B-55kDa in adenovirus-infected cells.	
			TGFB1	190 180	This gene encodes a member of the transforming growth factor beta (TGFB) family of cytokines, which are multifunctional peptides that regulate proliferation, differentiation, adhesion, migration, and other functions in many cell types. Many cells have TGFB receptors, and the protein positively and negatively regulates many other growth factors.	Camurati-Engelmann disease<br>{Cystic fibrosis lung disease, modifier of}
			B9D2	611 951	The gene is upregulated during mucociliary differentiation, and the encoded protein localizes to basal bodies and cilia.	Meckel syndrome 10
			EXOSC5	606 492	exosome component 5	
			BCKDHA	608 348	The branched-chain alpha-keto acid	Maple syrup urine disease,

					(BCAA) dehydrogenase (BCKD) complex is an innter mitochondrial enzyme complex that catalyzes the second major step in the catabolism of the branched-chain amino acids leucine, isoleucine, and valine.	type Ia
			CEACAM5	114 890	Carcinoembryonic antigen-related cell adhesion molecule 5	
			CEACAM6	163 980	Carcinoembryonic antigen is one of the most widely used tumor markers in serum immunoassay determinations of carcinoma.	
			CEACAM3	609 142	This gene encodes a member of the family of carcinoembryonic antigen-related cell adhesion molecules (CEACAMs), which are used by several bacterial pathogens to bind and invade host cells.	
			RPS19	603 474	This gene encodes a ribosomal protein that is a component of the 40S subunit	Diamond-Blackfan anemia 1

			CD79A	112 205	This gene encodes the Ig-alpha protein of the B-cell antigen component.	Agammaglobulinemia 3
			ARHGEF1	601 855	Rho guanine nucleotide exchange factor (GEF) 1	
			RABAC1	604 925	Rab acceptor 1 (prenylated)	
			ATP1A3	182 350	The protein encoded by this gene belongs to the family of P-type cation transport ATPases, and to the subfamily of Na+/K+ -ATPases. Na+/K+ -ATPase is an integral membrane protein responsible for establishing and maintaining the electrochemical gradients of Na and K ions across the plasma membrane.	Dystonia-12
			GRIK5	600 283	The protein encoded by this gene forms functional heteromeric kainate-preferring ionic channels with the subunits encoded by related gene family members. [	

			POU2F2	164 176	The encoded protein binds the octamer sequence 5'-ATTTGCAT-3', a common transcription factor binding site in immunoglobulin gene promoters.	
			GSK3A	606 784	This gene encodes a multifunctional Ser/Thr protein kinase that is implicated in the control of several regulatory proteins including glycogen synthase, and transcription factors, such as JUN. It also plays a role in the WNT and PI3K signaling pathways, as well as regulates the production of beta-amyloid peptides associated with Alzheimer's disease.	
			ERF	611 888	Members of the ETS family of transcription factors, such as ERF, regulate cell proliferation and differentiation.	
			CIC	612 082	capicua homolog (Drosophila)	

			PAFAH1B3	603 074	This gene encodes an acetylhydrolase that catalyzes the removal of an acetyl group from the glycerol backbone of platelet-activating factor.	
			MEGF8	604 267	multiple EGF-like-domains 8	
			CNFN	611 764	cornifelin	
			LIPE	151 750	The long form is expressed in steroidogenic tissues such as testis, where it converts cholesteryl esters to free cholesterol for steroid hormone production. The short form is expressed in adipose tissue, among others, where it hydrolyzes stored triglycerides to free fatty acids.	
			CXCL17	611 387	chemokine (C-X-C motif) ligand 17	
			CEACAM1	109 770	The encoded protein mediates cell adhesion via homophilic as well as heterophilic binding to other proteins of the subgroup.	
			PSG3	176 392	The human pregnancy-specific	

					glycoproteins (PSGs) are a family of proteins that are synthesized in large amounts by placental trophoblasts and released into the maternal circulation during pregnancy.	
			PSG8	176 397	The human pregnancy-specific glycoproteins (PSGs) are a group of molecules that are mainly produced by the placental syncytiotrophoblasts during pregnancy.	
			PSG10P	176 399	The human pregnancy-specific glycoproteins (PSGs) are a group of molecules that are mainly produced by the placental syncytiotrophoblasts during pregnancy.	
			PSG1	176 390	The human placenta is a multihormonal endocrine organ that produces hormones, enzymes, and other molecules that support fetal survival and development. Pregnancy-specific	

					beta-1-glycoprotein (PSBG, PSG) is a major product of the syncytiotrophoblast, reaching concentrations of 100 to 290 mg/l at term in the serum of pregnant women	
			PSG6	176 395	The human pregnancy-specific glycoproteins (PSGs) are a group of molecules that are mainly produced by the placental syncytiotrophoblasts during pregnancy.	
			PSG7	176 396	The PSG genes are expressed by placental trophoblasts and released into the maternal circulation during pregnancy, and are thought to be essential for maintenance of normal pregnancy.	
			PSG11	176 401	The human pregnancy-specific glycoproteins (PSGs) are a group of molecules that are mainly produced by the placental syncytiotrophoblasts during pregnancy.	

			PSG5	176 394	he human pregnancy-specific glycoproteins (PSGs) are a group of molecules that are mainly produced by the placental syncytiotrophoblasts during pregnancy.	
			PSG4	176 393	The human pregnancy-specific glycoproteins (PSGs) are a family of proteins that are synthesized in large amounts by placental trophoblasts and released into the maternal circulation during pregnancy.	
			PSG9	176 398	The human pregnancy-specific glycoproteins (PSGs) are a group of molecules that are mainly produced by the placental syncytiotrophoblasts during pregnancy.	
			PRG1	605 157	p53-responsive gene 1	
**3**	**20q12.21q11.22**	**deletion**				
			DEFB118	607 650	This gene encodes a member of the beta subfamily of defensins. Beta-defensins are antimicrobial peptides	

					that protect tissues and organs from infection by a variety of microorganisms.	
			REM1	610 388	The encoded protein is expressed in endothelial cells, where it promotes reorganization of the actin cytoskeleton and morphological changes in the cells.	
			HM13	607 106	The protein encoded by this gene, which localizes to the endoplasmic reticulum, catalyzes intramembrane proteolysis of some signal peptides after they have been cleaved from a preprotein. This activity is required to generate signal sequence-derived human lymphocyte antigen-E epitopes that are recognized by the immune system, and to process hepatitis C virus core protein. The encoded protein is an integral membrane	

					protein with sequence motifs characteristic of the presenilin-type aspartic proteases.	
			ID1	600 349	The encoded protein has no DNA binding activity and therefore can inhibit the DNA binding and transcriptional activation ability of basic HLH proteins with which it interacts. This protein may play a role in cell growth, senescence, and differentiation.	
			COX4I2	607 976	Cytochrome c oxidase (COX), the terminal enzyme of the mitochondrial respiratory chain, catalyzes the electron transfer from reduced cytochrome c to oxygen.	Exocrine pancreatic insufficiency, dyserythropoietic anemia, and calvarial hyperostosis
			BCL2L1	600 039	The proteins encoded by this gene are located at the outer mitochondrial membrane, and have been shown to regulate outer mitochondrial membrane channel (VDAC) opening.	

			TPX2	605 917	TPX2, microtubule-associated, homolog (Xenopus laevis)	
			MYLK2	606 566	This gene encodes a myosin light chain kinase, a calcium/calmodulin dependent enzyme, that is exclusively expressed in adult skeletal muscle. [	Cardiomyopathy, hypertrophic, midventricular, digenic
			FOXS1	602 939	The protein encoded by this intronless gene contains a forkhead domain and is found predominantly in aorta and kidney. The function of the encoded protein is unknown.	
			PDRG1	610 789	p53 and DNA-damage regulated 1	
			HCK	142 370	This protein is primarily hemopoietic, particularly in cells of the myeloid and B-lymphoid lineages. It may help couple the Fc receptor to the activation of the respiratory burst. In addition, it may play a role in neutrophil migration and in the degranulation of neutrophils.	

			PLAGL2	604 866	pleiomorphic adenoma gene-like 2	
			POFUT1	607 491	This gene encodes a member of the glycosyltransferase O-Fuc family. This enzyme adds O-fucose through an O-glycosidic linkage to conserved serine or threonine residues in the epidermal growth factor-like repeats of a number of cell surface and secreted proteins. O-fucose glycans are involved in ligand-induced receptor signaling.	
			KIF3B	603 754	The protein encoded by this gene acts as a heterodimer with kinesin family member 3A to aid in chromosome movement during mitosis and meiosis. The encoded protein is a plus end-directed microtubule motor and can interact with the SMC3 subunit of the cohesin complex. In addition, the encoded protein may be involved in the	

					intracellular movement of membranous organelles	
			ASXL1	612 990	The protein is a member of the Polycomb group of proteins, which are necessary for the maintenance of stable repression of homeotic and other loci. The protein is thought to disrupt chromatin in localized areas, enhancing transcription of certain genes while repressing the transcription of other genes. The protein encoded by this gene functions as a ligand-dependent co-activator for retinoic acid receptor in cooperation with nuclear receptor coactivator 1.	Bohring-Opitz syndrome Myelodysplastic syndrome, somatic
			DNMT3B	602 900	CpG methylation is an epigenetic modification that is important for embryonic development, imprinting, and X-chromosome inactivation.	Immunodeficiency-centromeric instability-facial anomalies syndrome 1
			MAPRE1	603 108	The protein also associates with	

					components of the dynactin complex and the intermediate chain of cytoplasmic dynein. Because of these associations, it is thought that this protein is involved in the regulation of microtubule structures and chromosome stability.	
			SUN5	613 942	Sad1 and UNC84 domain containing 5	
			BPIFB2	614 108	This gene encodes a member of the lipid transfer/lipopolysaccharide binding protein (LT/LBP) gene family. It is highly expressed in hypertrophic tonsils.	
			BPIFB6	614 110	BPI fold containing family B, member 6	
			BPIFA4P	607 627	BPI fold containing family A, member 4, pseudogene	
			BPIFA1	607 412	he exact biological function of this gene is not known, however, it has been suggested to be involved in	

					inflammatory responses to irritants in the upper airways. It may also serve as a potential molecular marker for detection of micrometastasis in non-small-cell lung cancer.	
			CDK5RAP1	608 200	Neuronal CDC2-like kinase, which is involved in the regulation of neuronal differentiation, is composed of a catalytic subunit, CDK5, and an activating subunit, p25NCK5A. The protein encoded by this gene binds to p25NCK5A and therefore may be involved in neuronal differentiation.	
			SNTA1	601 017	The protein encoded by this gene is a peripheral membrane protein found associated with dystrophin and dystrophin-related proteins.	Long QT syndrome 12
**4**	**9q21.33**	**duplication**				
			DAPK1	600 831	Death-associated protein kinase 1 is a positive mediator of gamma-interferon induced programmed cell	

					death.<br>DAPK1 encodes a structurally unique 160-kD calmodulin dependent serine-threonine kinase that carries 8 ankyrin repeats and 2 putative P-loop consensus sites. It is a tumor suppressor candidate.	
	11q24.1q25	deletion				
			SORL1	602 005	This gene encodes a mosaic protein that belongs to at least two families: the vacuolar protein sorting 10 (VPS10) domain-containing receptor family, and the low density lipoprotein receptor (LDLR) family.<br>The encoded protein also contains fibronectin type III repeats and an epidermal growth factor repeat. The encoded protein is translated as a preproprotein and likely plays roles in	{Alzheimer disease, pathogenesis, association with}

					endocytosis and sorting. There may be an association between expression of this locus and Alzheimer's Disease	
			MIR125B1	610 104	microRNAs (miRNAs) are short (20-24 nt) non-coding RNAs that are involved in post-transcriptional regulation of gene expression in multicellular organisms by affecting both the stability and translation of mRNAs.	
			BLID	608 853	This gene encodes a BH3-like motif containing protein involved in cell death. The encoded protein may induce apoptosis in a caspase-dependent manner.	
			MIRLET7A2	612 142	microRNAs (miRNAs) are short (20-24 nt) non-coding RNAs that are involved in post-transcriptional regulation of gene expression in multicellular organisms by affecting	

					both the stability and translation of mRNAs.	
			MIR100	613 186	microRNAs (miRNAs) are short (20-24 nt) non-coding RNAs that are involved in post-transcriptional regulation of gene expression in multicellular organisms by affecting both the stability and translation of mRNAs.	
			UBASH3B	609 201	This gene encodes a protein that contains a ubiquitin associated domain at the N-terminus, an SH3 domain, and a C-terminal domain with similarities to the catalytic motif of phosphoglycerate mutase. The encoded protein was found to inhibit endocytosis of epidermal growth factor receptor (EGFR) and platelet-derived growth factor receptor.	
			CRTAM	612 597	The CRTAM gene is upregulated in	

					CD4 (see MIM 186940)-positive and CD8 (see CD8A; MIM 186910)-positive T cells and encodes a type I transmembrane protein with V and C1-like Ig domains	
			BSX	611 074	brain-specific homeobox	
			HSPA8	600 816	This gene encodes a member of the heat shock protein 70 family, which contains both heat-inducible and constitutively expressed members. This protein belongs to the latter group, which are also referred to as heat-shock cognate proteins.	
			ASAM	611 693	The CTX (see VSIG2, MIM 606011) family of proteins, including ASAM, are type I transmembrane proteins within the Ig superfamily that localize to junctional complexes between endothelial and epithelial cells and may play a role in cell-cell adhesion	
			SCN3B	608 214	This gene encodes one member of	

					the sodium channel beta subunit gene family, and influences the inactivation kinetics of the sodium channel.	
			ZNF202	603 430	zinc finger protein 202	Brugada syndrome 7
			VWA5A	602 929	von Willebrand factor A domain containing 5A	
			PANX3	608 422	The protein encoded by this gene belongs to the innexin family. Innexin family members are known to be the structural components of gap junctions.	
			TBRG1	610 614	transforming growth factor beta regulator 1	
			SIAE	610 079	This gene encodes an enzyme which removes 9-O-acetylation modifications from sialic acids.	{Autoimmune disease, susceptibility to, 6}
			SPA17	608 621	This gene encodes a protein present at the cell surface.	
			NRGN	602 350	Neurogranin (NRGN) is the human homolog of the neuron-specific rat	

					RC3/neurogranin gene. This gene encodes a postsynaptic protein kinase substrate that binds calmodulin in the absence of calcium.	
			VSIG2	606 011	V-set and immunoglobulin domain containing 2	
			ROBO3	608 630	The ROBO3 gene regulates axonal navigation at the ventral midline of the neural tube. In mouse, loss of Robo3 results in a complete failure of commissural axons to cross the midline throughout the spinal cord and the hindbrain.	Gaze palsy, horizontal, with progressive scoliosis
			ROBO4	607 528	roundabout, axon guidance receptor, homolog 4 (Drosophila)	
			HEPN1	611 641	Transient transfection studies showed that expression of this gene significantly inhibited cell growth, and it may have a role in apoptosis. Expression of this gene is downregulated or lost in	

					hepatocellular carcinomas (HCC), suggesting that loss of this gene is involved in carcinogenesis of hepatocytes (PMID:12971969).	
			HEPACAM	611 642	The protein encoded by this gene is a single-pass type I membrane protein that localizes to the cytoplasmic side of the cell membrane. The encoded protein acts as a homodimer and is involved in cell motility and cell-matrix interactions.	Megalencephalic leukoencephalopathy with subcortical cysts 2A Megalencephalic leukoencephalopathy with subcortical cysts 2B, remitting, with or without mental retardation
			PKNOX2	613 066	Homeodomain proteins are sequence-specific transcription factors that share a highly conserved DNA-binding domain and play fundamental roles in cell proliferation, differentiation, and death.	
			FEZ1	604 825	This gene is an ortholog of the C. elegans unc-76 gene, which is necessary for normal axonal bundling	

					and elongation within axon bundles.	
			EI24	605 170	This gene has higher expression in p53-expressing cells than in control cells and is an immediate-early induction target of p53-mediated apoptosis. The protein encoded by this gene contains six putative transmembrane domains and may suppress cell growth by inducing apoptotic cell death through the caspase 9 and mitochondrial pathways.	
			STT3A	601 134	STT3, subunit of the oligosaccharyltransferase complex, homolog A (S. cerevisiae)	
			CHEK1	603 078	This protein acts to integrate signals from ATM and ATR, two cell cycle proteins involved in DNA damage responses,<br>that also associate with chromatin in meiotic prophase I. Phosphorylation	

					of CDC25A protein phosphatase by this protein is required for cells to delay cell cycle progression in response to double-strand DNA breaks.	
			ACRV1	102 525	This gene encodes a testis-specific, differentiation antigen, acrosomal vesicle protein 1, that arises within the acrosomal vesicle during spermatogenesis, and is associated with the acrosomal membranes and matrix of mature sperm.	
			PATE1	606 861	prostate and testis expressed 1	
			HYLS1	610 693	This gene encodes a protein localized to the cytoplasm.	Hydrolethalus syndrome
			DDX25	607 663	The encoded protein is a gonadotropin-regulated and developmentally expressed testicular RNA helicase. It may serve to maintain testicular functions related to steroidogenesis and	

					spermatogenesis.	
			CDON	608 707	This gene encodes a cell surface receptor that is a member of the immunoglobulin superfamily.	Holoprosencephaly 11
			SRPR	182 180	The gene encodes a subunit of the endoplasmic reticulum signal recognition particle receptor that, in conjunction with the signal recognition particle, is involved in the targeting and translocation of signal sequence tagged secretory and membrane proteins across the endoplasmic reticulum.	
			FOXRED1	613 622	This gene encodes a protein that contains a FAD-dependent oxidoreductase domain. The encoded protein is localized to the mitochondria and may function as a chaperone protein required for the function of mitochondrial complex I.	Leigh syndrome due to mitochondrial complex I deficiency Mitochondrial complex I deficiency

			TIRAP	606 252	The protein encoded by this gene is a TIR adaptor protein involved in the TLR4 signaling pathway of the immune system. It activates NF-kappa-B, MAPK1, MAPK3 and JNK, which then results in cytokine secretion and the inflammatory response.	{Bacteremia, protection against}<br>{Malaria, protection against}<br>{Pneumococcal disease, invasive, protection against}<br>{Tuberculosis, protection against}
			DCPS	610 534	decapping enzyme, scavenger	
			ST3GAL4	104 240	This gene encodes a member of the glycosyltransferase 29 family, a group of enzymes involved in protein glycosylation.	
			KIRREL3	607 761	The protein encoded by this gene is a member of the nephrin-like protein family.<br>These proteins are expressed in fetal and adult brain, and also in podocytes of kidney glomeruli. The cytoplasmic domains of these proteins interact with the C-terminus	Mental retardation, autosomal dominant 4

					of podocin, also expressed in the podocytes, cells involved in ensuring size- and charge-selective ultrafiltration.	
			ETS1	164 720	These proteins function either as transcriptional activators or repressors of numerous genes, and are involved in stem cell development, cell senescence and death, and tumorigenesis.	
			FLI1	193 067	Friend leukemia virus integration 1	
			KCNJ1	600 359	The protein encoded by this gene is an integral membrane protein and inward-rectifier type potassium channel.<br>It is activated by internal ATP and probably plays an important role in potassium homeostasis. The encoded protein has a greater tendency to allow potassium to flow into a cell rather than out of a cell.	Bartter syndrome, type 2

			KCNJ5	600 734	The protein encoded by this gene is an integral membrane protein and inward-rectifier type potassium channel. The encoded protein, which has a greater tendency to allow potassium to flow into a cell rather than out of a cell, is controlled by G-proteins.	Hyperaldosteronism, familial, type III<br>Long QT syndrome 13
			TP53AIP1	605 426	This gene is specifically expressed in the thymus, and encodes a protein that is localized to the mitochondrion. The expression of this gene is inducible by p53, and it is thought to play an important role in mediating p53-dependent apoptosis.	
			ARHGAP32	608 541	RICS is a neuron-associated GTPase-activating protein that may regulate dendritic spine morphology and strength by modulating Rho GTPase	
			LOC100188	194 630	zinc finger protein 123, pseudogene	

			891			
			BARX2	604 823	This gene encodes a member of the homeobox transcription factor family. A highly related protein in mouse has been shown to influence cellular processes that control cell adhesion and remodeling of the actin cytoskeleton in myoblast fusion and chondrogenesis. The encoded protein may also play a role in cancer progression.	
			NFRKB	164 013	nuclear factor related to kappaB binding protein	
			APLP2	104 776	This protein interacts with major histocompatibility complex (MHC) class I molecules. The synergy of this protein and the APP is required to mediate neuromuscular transmission, spatial learning and synaptic plasticity. This protein has been implicated in the pathogenesis of	

					Alzheimer's disease.	
			ST14	606 797	The protein encoded by this gene is an epithelial-derived, integral membrane serine protease. This protease forms a complex with the Kunitz-type serine protease inhibitor, HAI-1, and is found to be activated by sphingosine 1-phosphate.	Ichthyosis with hypotrichosis
			ADAMTS8	605 175	The enzyme encoded by this gene contains two C-terminal TS motifs, and disrupts angiogenesis in vivo.	
			ADAMTS15	607 509	This gene encodes a member of the ADAMTS (a disintegrin and metalloproteinase with thrombospondin motifs) protein family. ADAMTS family members share several distinct protein modules, including a propeptide region, a metalloproteinase domain, a disintegrin-like domain, and a	

					thrombospondin type 1 (TS) motif.	
			NTM	607 938	The encoded protein may promote neurite outgrowth and adhesion via a homophilic mechanism.	
			OPCML	600 632	This gene encodes a member of the IgLON subfamily in the immunoglobulin protein superfamily. The encoded protein is localized in the plasma membrane and may have an accessory role in opioid receptor function. This gene has an ortholog in rat and bovine.	{Ovarian cancer, somatic}
			SPATA19	609 805	spermatogenesis associated 19	
			IGSF9B	613 773	immunoglobulin superfamily, member 9B	
			JAM3	606 871	The protein encoded by this immunoglobulin superfamily gene member is localized in the tight junctions between high endothelial cells.	Hemorrhagic destruction of the brain, subependymal calcification, and cataracts
			NCAPD3	609 276	Condensin complexes I and II play	

					essential roles in mitotic chromosome assembly and segregation.	
			VPS26B	610 027	vacuolar protein sorting 26 homolog B (S. pombe)	
			THYN1	613 739	This gene encodes a protein that is highly conserved among vertebrates and plant species and may be involved in the induction of apoptosis.	
			ACAD8	604 773	This gene encodes a member of the acyl-CoA dehydrogenase family of enzymes that catalyze the dehydrogenation of acyl-CoA derivatives in the metabolism of fatty acids or branch chained amino acids. The encoded protein is a mitochondrial enzyme that functions in catabolism of the branched-chain amino acid valine.	Isobutyryl-CoA dehydrogenase deficiency
			B3GAT1	151 290	The protein encoded by this gene is a member of the glucuronyltransferase gene family. These enzymes exhibit	

					strict acceptor specificity, recognizing nonreducing terminal sugars and their anomeric linkages. This gene product functions as the key enzyme in a glucuronyl transfer reaction during the biosynthesis of the carbohydrate epitope HNK-1 (human natural killer-1, also known as CD57 and LEU7).	
	18p11.31	deletion				
			DLGAP1	605 445	discs, large (Drosophila) homolog-associated protein 1	
**6**	**4p16.3p16.1**	**deletion**				
			ZNF141	194 648	zinc finger protein 141	
			PDE6B	180 072	Photon absorption triggers a signaling cascade in rod photoreceptors that activates cGMP phosphodiesterase (PDE), resulting in the rapid hydrolysis of cGMP, closure of cGMP-gated cation channels, and hyperpolarization of the cell. PDE is a peripheral membrane heterotrimeric	Night blindness, congenital stationary, autosomal dominant 2<br>Retinitis pigmentosa-40

					enzyme made up of alpha, beta, and gamma subunits.<br>This gene encodes the beta subunit.	
			ATP5I	601 519	Mitochondrial ATP synthase catalyzes ATP synthesis, utilizing an electrochemical gradient of protons across the inner membrane during oxidative phosphorylation. It is composed of two linked multi-subunit complexes: the soluble catalytic core, F1,<br>and the membrane-spanning component, Fo, which comprises the proton channel. The F1 complex consists of 5 different subunits (alpha, beta, gamma, delta, and epsilon) assembled in a ratio of 3 alpha, 3 beta, and a single representative of the other 3.<br>The Fo seems to have nine subunits (a, b, c, d, e, f, g, F6 and 8). This	

					gene encodes the e subunit of the Fo complex.	
			MYL5	160 782	This gene encodes one of the myosin light chains, a component of the hexameric ATPase cellular motor protein myosin.	
			CPLX1	605 032	Proteins encoded by the complexin/synaphin gene family are cytosolic proteins that function in synaptic vesicle exocytosis. These proteins bind syntaxin, part of the SNAP receptor.<br>The protein product of this gene binds to the SNAP receptor complex and disrupts it, allowing transmitter release.	
			GAK	602 052	Cyclin G is a direct transcriptional target of the p53 tumor suppressor gene product and thus functions downstream of p53.	

			DGKQ	601 207	The protein encoded by this gene contains three cysteine-rich domains, a proline-rich region, and a pleckstrin homology domain with an overlapping Ras-associating domain. It is localized in the speckle domains of the nucleus, and mediates the regeneration of phosphatidylinositol (PI) from diacylglycerol in the PI-cycle during cell signal transduction.	
			SLC26A1	610 130	This gene is a member of a family of sulfate/anion transporter genes. Family members are well conserved in their genomic (number and size of exons) and protein (aa length among species) structures, but have markedly different tissue expression patterns.	
			IDUA	252 800	This gene encodes an enzyme that hydrolyzes the terminal alpha-L-	Mucopolysaccharidosis Ih Mucopolysaccharidosis Ih/s

					iduronic acid residues of two glycosaminoglycans, dermatan sulfate and heparan sulfate.	Mucopolysaccharidosis Is
			FGFRL1	605 830	The protein encoded by this gene is a member of the fibroblast growth factor receptor (FGFR) family, where amino acid sequence is highly conserved between members and throughout evolution.	
			RNF212	612 041	This gene encodes a RING finger protein that may function as a ubiquitin ligase. The encoded protein may be involved in meiotic recombination.	Recombination rate QTL 1
			SPON2	605 918	spondin 2, extracellular matrix protein	
			CTBP1	602 618	This gene encodes a protein that binds to the C-terminus of adenovirus E1A proteins. This phosphoprotein is a transcriptional repressor and may play a role during cellular proliferation.	

			MAEA	606 801	This gene product mediates the attachment of erythroblasts to macrophages. This attachment promotes terminal maturation and enucleation of erythroblasts, presumably by suppressing apoptosis.	
			CRIPAK	610 203	cysteine-rich PAK1 inhibitor	
			SLBP	602 422	This gene encodes a protein that binds to the stem-loop structure in replication-dependent histone mRNAs.	
			TACC3	605 303	This gene encodes a member of the transforming acidic colied-coil protein family. The encoded protein is a motor spindle protein that may play a role in stabilization of the mitotic spindle.	{?Bladder cancer susceptibility}

			FGFR3	134 934	This gene encodes a member of the fibroblast growth factor receptor (FGFR) family, with its amino acid sequence being highly conserved between members and among divergent species. FGFR family members differ from one another in their ligand affinities and tissue distribution.	Achondroplasia<br>Bladder cancer, somatic<br>CATSHL syndrome<br>Cervical cancer, somatic<br>Colorectal cancer, somatic<br>Crouzon syndrome with acanthosis nigricans<br>Hypochondroplasia<br>LADD syndrome<br>Muenke syndrome<br>Nevus, keratinocytic, nonepidermolytic<br>Spermatocytic seminoma, somatic<br>Thanatophoric dysplasia, type I<br>Thanatophoric dysplasia, type II
			LETM1	604 407	This gene encodes a protein that is localized to the inner mitochondrial membrane. The protein functions to	

					maintain the mitochondrial tubular shapes and is required for normal mitochondrial morphology and cellular viability.	
			WHSC1	602 952	This gene encodes a protein that contains four domains present in other developmental proteins:<br>a PWWP domain, an HMG box, a SET domain, and a PHD-type zinc finger. It is expressed ubiquitously in early development.Wolf-Hirschhorn syndrome (WHS) is a malformation syndrome associated with a hemizygous deletion of the distal short arm of chromosome 4.	
			WHSC2	606 026	This gene is expressed ubiquitously with higher levels in fetal than in adult tissues.<br>It encodes a protein sharing 93% sequence identity with the mouse protein. Wolf-Hirschhorn syndrome	

					(WHS) is a malformation syndrome associated with a hemizygous deletion of the distal short arm of chromosome 4. This gene is mapped to the 165 kb WHS critical region, and may play a role in the phenotype of the WHS or Pitt-Rogers-Danks syndrome.	
			NAT8L	610 647	This gene encodes a single-pass membrane protein, which contains a conserved sequence of the GCN5 or NAT superfamily of N-acetyltransferases and is a member of the N-acyltransferase (NAT) superfamily. This protein is a neuron-specific protein and is the N-acetylaspartate (NAA) biosynthetic enzyme, catalyzing the NAA synthesis from L-aspartate and acetyl-CoA.	N-acetylaspartate deficiency
			POLN	610 887	polymerase (DNA directed) nu	

			HAUS3	613 430	HAUS3 is 1 of 8 subunits of the 390-kD human augmin complex, or HAUS complex.	
			ZFYVE28	614 176	zinc finger, FYVE domain containing 28	
			RNF4	602 850	The protein encoded by this gene contains a RING finger motif and acts as a transcription regulator. This protein has been shown to interact with, and inhibit the activity of, TRPS1, a transcription suppressor of GATA-mediated transcription.	
			TNIP2	610 669	TNIP2 binds to the C-terminal zinc finger domain of A20 (TNFAIP3; MIM 191163) and is involved in activation of the ERK (see MAPK3; MIM 601795) MAP kinase pathway in various cell types	
			SH3BP2	602 104	The protein encoded by this gene has an N-terminal pleckstrin homology (PH) domain,	Cherubism

					an SH3-binding proline-rich region, and a C-terminal SH2 domain. The protein binds to the SH3 domains of several proteins including the ABL1 and SYK protein tyrosine kinases , and functions as a cytoplasmic adaptor protein to positively regulate transcriptional activity in T, natural killer (NK), and basophilic cells.	
			ADD1	102 680	Adducins are a family of cytoskeleton proteins encoded by three genes (alpha, beta, gamma). Adducin is a heterodimeric protein that consists of related subunits, which are produced from distinct genes but share a similar structure.	{Hypertension, essential, salt-sensitive}
			MFSD10	610 977	This gene encodes a member of the major facilitator superfamily of transporter proteins. The encoded protein likely functions in efflux of organic anions, including the non-	

					steroidal anti-inflammatory drugs indomethacin and diclofenac.	
			NOP14	611 526	NOP14 plays a role in the processing of the pre-18S rRNA and small ribosomal subunit assembly	
			GRK4	137 026	This gene encodes a member of the guanine nucleotide-binding protein (G protein)-coupled receptor kinase subfamily of the Ser/Thr protein kinase family.	
			HTT	613 004	Huntingtin is a disease gene linked to Huntington's disease, a neurodegenerative disorder characterized by loss of striatal neurons. This is thought to be caused by an expanded, unstable trinucleotide repeat in the huntingtin gene, which translates as a polyglutamine repeat in the protein product.	Huntington disease
			RGS12	602 512	This gene encodes a member of the	

					'regulator of G protein signaling' (RGS) gene family. The encoded protein may function as a guanosine triphosphatase (GTPase)-activating protein as well as a transcriptional repressor. This protein may play a role in tumorigenesis.	
			HGFAC	604 552	{the encoded protein]It acts as serine protease that converts hepatocyte growth factor to the active form.	
			DOK7	610 285	The protein encoded by this gene is essential for neuromuscular synaptogenesis.	Fetal akinesia deformation sequence<br>Myasthenia, limb-girdle, familial
			LRPAP1	104 225	low density lipoprotein receptor-related protein associated protein 1	
			ADRA2C	104 250	Alpha-2-adrenergic receptors are members of the G protein-coupled receptor superfamily.<br>They include 3 highly homologous subtypes: alpha2A, alpha2B, and	{Congestive heart failure and beta-blocker response, modifier of}

					alpha2C. These receptors have a critical role in regulating neurotransmitter release from sympathetic nerves and from adrenergic neurons in the central nervous system.	
			OTOP1	607 806	otopetrin 1	
			D4S234E	607 645	DNA segment on chromosome 4 (unique) 234 expressed sequence	
			MSX1	142 983	This gene encodes a member of the muscle segment homeobox gene family. The encoded protein functions as a transcriptional repressor during embryogenesis through interactions with components of the core transcription complex and other homeoproteins.	Orofacial cleft<br>Tooth agenesis, selective, 1, with or without orofacial cleft<br>Witkop syndrome
			CYTL1	607 930	cytokine-like 1	
			EVC2	607 261	This gene encodes a protein that functions in bone formation and skeletal development.	Ellis-van Creveld syndrome

			EVC	604 831	This gene encodes a protein containing a leucine zipper and a transmembrane domain.	4p16.2 Ellis-van Creveld syndrome<br>Weyers acrodental dysostosis
			CRMP1	602 462	This gene encodes a member of a family of cytosolic phosphoproteins expressed exclusively in the nervous system.<br>The encoded protein is thought to be a part of the semaphorin signal transduction pathway implicated in semaphorin-induced growth cone collapse during neural development.	
			JAKMIP1	611 195	janus kinase and microtubule interacting protein 1	
			WFS1	606 201	This gene encodes a transmembrane protein, which is located primarily in the endoplasmic reticulum and ubiquitously expressed with highest levels in brain, pancreas, heart, and	Deafness, autosomal dominant<br>Wolfram syndrome<br>Wolfram-like syndrome, autosomal dominant

					insulinoma beta-cell lines.	{Diabetes mellitus, noninsulin-dependent, association with}
			PPP2R2C	605 997	This gene encodes a gamma isoform of the regulatory subunit B55 subfamily.	
			S100P	600 614	The protein encoded by this gene is a member of the S100 family of proteins containing 2 EF-hand calcium-binding motifs. S100 proteins are localized in the cytoplasm and/or nucleus of a wide range of cells, and involved in the regulation of a number of cellular processes such as cell cycle progression and differentiation.	
			CNO	605 695	This intronless gene encodes a protein that may play a role in organelle biogenesis associated with melanosomes, platelet dense granules, and lysosomes.	

			TADA2B	608 790	TADA2B functions as a transcriptional adaptor protein that potentiates transcription through coordination of histone acetyltransferase (HAT) activity and by linking activation factors to basal transcriptional machinery	
			GRPEL1	606 173	GrpE-like 1, mitochondrial (E. coli)	
			SORCS2	606 284	This gene encodes one family member of vacuolar protein sorting 10 (VPS10) domain-containing receptor proteins.	
			AFAP1	608 252	The protein encoded by this gene is a Src binding partner. It may represent a potential modulator of actin filament integrity in response to cellular signals, and may function as an adaptor protein by linking Src family members and/or other signaling proteins to actin filaments.	
			MIR95	613 185	microRNA 95	

			ABLIM2	612 544	actin binding LIM protein family, member 2	
			HTRA3	608 785	HtrA serine peptidase 3	
			ACOX3	603 402	Acyl-Coenzyme A oxidase 3 also know as pristanoyl -CoA oxidase (ACOX3)is involved in the desaturation of 2-methyl branched fatty acids in peroxisomes.	
			GPR78	606 921	The protein encoded by this gene belongs to the G protein-coupled receptor family, which contain 7 transmembrane domains and transduce extracellular signals through heterotrimeric G proteins. This is an orphan receptor, which displays significant level of constitutive activity.	
			CPZ	603 105	This gene encodes a member of the metallocarboxypeptidase family. This enzyme displays carboxypeptidase activity towards substrates with basic	

					C-terminal residues. It is most active at neutral pH and is inhibited by active site-directed inhibitors of metallocarboxypeptidases.	
	8p23.3p23.1	duplication				
			FBXO25	609 098	This gene encodes a member of the F-box protein family which is characterized by an approximately 40 amino acid motif, the F-box. The F-box proteins constitute one of the four subunits of ubiquitin protein ligase complex called SCFs (SKP1-cullin-F-box), which function in phosphorylation-dependent ubiquitination.	
			DLGAP2	605 438	The product of this gene is one of the membrane-associated guanylate kinases localized at postsynaptic density in neuronal cells.	
			CLN8	607 837	This gene encodes a transmembrane	Ceroid lipofuscinosis,

	[redacted]	[redacted]			protein belonging to a family of proteins containing TLC domains, which are postulated to function in lipid synthesis, transport, or sensing.	neuronal, 8<br>Ceroid lipofuscinosis, neuronal, 8, Northern epilepsy variant
	[redacted]	[redacted]	ARHGEF10	608 136	Rho GTPases play a fundamental role in numerous cellular processes that are initiated by extracellular stimuli that work<br>through G protein coupled receptors. The encoded protein may form complex with G proteins and stimulate Rho-dependent signals.	Slowed nerve conduction velocity, AD
	[redacted]	[redacted]	MYOM2	603 509	The giant protein titin, together with its associated proteins, interconnects the major structure of sarcomeres, the M bands and Z discs.	
	[redacted]	[redacted]	CSMD1	608 397	CUB and Sushi multiple domains 1	
	[redacted]	[redacted]	MCPH1	607 117	This gene encodes a DNA damage response protein.	Microcephaly, primary autosomal recessive, 1
	[redacted]	[redacted]	ANGPT2	601 922	The protein encoded by this gene is an antagonist of angiopoietin 1	

					(ANGPT1) and endothelial TEK tyrosine kinase (TIE-2, TEK). The encoded protein disrupts the vascular remodeling ability of ANGPT1 and may induce endothelial cell apoptosis.	
	14q32.33	deletion				
			IGHA1	146 900	immunoglobulin heavy constant alpha 1	
			IGHG1	147 100	immunoglobulin heavy constant gamma 1 (G1m marker)	
	17p12	deletion				
			COX10	602 125	Cytochrome c oxidase (COX), the terminal component of the mitochondrial respiratory chain, catalyzes the electron transfer from reduced cytochrome c to oxygen.	Encephalopathy, progressive mitochondrial, with proximal renal tubulopathy due to cytochrome c oxidase deficiency
			HS3ST3B1	604 058	Heparan sulfate biosynthetic enzymes are key components in generating a myriad of distinct	

					heparan sulfate fine structures that carry out multiple biologic activities. The enzyme encoded by this gene is a member of the heparan sulfate biosynthetic enzyme family.	
**7**	**5p13.3**	**duplication**				
			NPR3	108 962	This gene encodes one of three natriuretic peptide receptors.	Hypertension, salt-resistant
**8**	**3p26.3p22.3**	**duplication**				
			CHL1	607 416	The protein encoded by this gene is a member of the L1 gene family of neural cell adhesion molecules. It is a neural recognition molecule that may be involved in signal transduction pathways. The deletion of one copy of this gene may be responsible for mental defects in patients with 3p- syndrome.	
			CNTN6	607 220	The protein encoded by this gene is a member of the immunoglobulin superfamily. It is a	

					glycosylphosphatidylinositol (GPI)-anchored neuronal membrane protein that functions as a cell adhesion molecule. It may play a role in the formation of axon connections in the developing nervous system.	
			CNTN4	607 280	This gene encodes a member of the contactin family of immunoglobulins.	
			IL5RA	147 851	The protein encoded by this gene is an interleukin 5 specific subunit of a heterodimeric cytokine receptor. The receptor is comprised of a ligand specific alpha subunit and a signal transducing beta subunit shared by the receptors for interleukin 3 (IL3), colony stimulating factor 2 (CSF2/GM-CSF), and interleukin 5 (IL5).	
			TRNT1	612 907	The CCA-adding enzyme TRNT1 (EC 2.7.7.25) is an essential enzyme that	

					catalyzes the addition of the CCA terminus to the 3-prime end of tRNA precursors.	
			CRBN	609 262	This gene encodes a protein related to the Lon protease protein family. In rodents and other mammals this gene product is found in the cytoplasm localized with a calcium channel membrane protein, and is thought to play a role in brain development. Mutations in this gene are associated with autosomal recessive nonsyndromic mental retardation.	Mental retardation, autosomal recessive 2
			SETMAR	609 834	SET domain and mariner transposase fusion gene	
			SUMF1	607 939	This gene encodes an enzyme that catalyzes the hydrolysis of sulfate esters by oxidizing a cysteine residue in the substrate sulfatase to an active site 3-oxoalanine residue, which is	Multiple sulfatase deficiency

	[redacted]	[redacted]			also known as C-alpha-formylglycine.	
	[redacted]	[redacted]	ITPR1	147 265	This gene encodes an intracellular receptor for inositol 1,4,5-trisphosphate. Upon stimulation by inositol 1,4,5-trisphosphate, this receptor mediates calcium release from the endoplasmic reticulum.	Spinocerebellar ataxia 15
	[redacted]	[redacted]	EGOT	611 662	eosinophil granule ontogeny transcript (non-protein coding)	
	[redacted]	[redacted]	BHLHE40	604 256	This gene encodes a basic helix-loop-helix protein expressed in various tissues.	
	[redacted]	[redacted]	EDEM1	607 673	ER degradation enhancer, mannosidase alpha-like 1	
	[redacted]	[redacted]	GRM7	604 101	L-glutamate is the major excitatory neurotransmitter in the central nervous system, and it activates both ionotropic and metabotropic glutamate receptors. Glutamatergic neurotransmission is involved in most aspects of normal	

					brain function and can be perturbed in many neuropathologic conditions.	
			LMCD1	604 859	The protein encoded by this gene contains a cysteine-rich domain in the N-terminal region and 2 LIM domains in the C-terminal region. It also has several potential phosphorylation and N-myristoylation sites and a single potential N-glycosylation site.	
			LINC00312	610 485	long intergenic non-protein coding RNA 312	
			CAV3	601 253	This gene encodes a caveolin family member, which functions as a component of the caveolae plasma membranes found in most cell types.	Cardiomyopathy, familial hypertrophic<br>Creatine phosphokinase, elevated serum<br>Long QT syndrome-9<br>Muscular dystrophy, limb-girdle, type IC<br>Myopathy, distal, Tateyama type<br>Rippling muscle disease

			OXTR	167 055	The protein encoded by this gene belongs to the G-protein coupled receptor family and acts as a receptor for oxytocin. Its activity is mediated by G proteins which activate a phosphatidylinositol-calcium second messenger system.	
			RAD18	605 256	The protein encoded by this gene is highly similar to S. cerevisiae DNA damage repair protein Rad18. Yeast Rad18 functions through its interaction with Rad6, which is an ubiquitin-conjugating enzyme required for post-replication repair of damaged DNA.	
			SRGAP3	606 525	SLIT-ROBO Rho GTPase activating protein 3	
			LHFPL4	610 240	This gene is a member of the lipoma HMGIC fusion partner (LHFP) gene family, which is a subset of the superfamily of tetraspan	

					transmembrane protein encoding genes.	
			MTMR14	611 089	This gene encodes a myotubularin-related protein.	{Centronuclear myopathy, autosomal, modifier of}
			BRPF1	602 410	The protein encoded by this gene is expressed ubiquitously and at the highest level in testes and spermatogonia.	
			OGG1	601 982	This gene encodes the enzyme responsible for the excision of 8-oxoguanine, a mutagenic base byproduct which occurs as a result of exposure to reactive oxygen.	Renal cell carcinoma, clear cell, somatic
			CAMK1	604 998	Calcium/calmodulin-dependent protein kinase I is expressed in many tissues and is a component of a calmodulin-dependent protein kinase cascade.	
			TADA3	602 945	The protein encoded by this gene is a transcriptional activator adaptor and has been found to be part of the	

					PCAF histone acetylase complex.	
			ARPC4	604 226	This gene encodes one of seven subunits of the human Arp2/3 protein complex. This complex controls actin polymerization in cells and has been conserved throughout eukaryotic evolution. This gene encodes the p20 subunit, which is necessary for actin nucleation and high-affinity binding to F-actin.	
			CIDEC	612 120	This gene encodes a member of the cell death-inducing DNA fragmentation factor-like effector family. Members of this family play important roles in apoptosis.	
			IL17RC	610 925	This gene encodes a single-pass type I membrane protein that shares similarity with the interleukin-17 receptor (IL-17RA). Unlike IL-17RA, which is predominantly expressed in hemopoietic cells, and binds with high	

					affinity to only IL-17A, this protein is expressed in nonhemopoietic tissues, and binds both IL-17A and IL-17F with similar affinities. The proinflammatory cytokines, IL-17A and IL-17F, have been implicated in the progression of inflammatory and autoimmune diseases.	
			CRELD1	607 170	This gene encodes a member of a subfamily of epidermal growth factor-related proteins. The encoded protein is characterized by a cysteine-rich with epidermal growth factor-like domain. This protein may function as a cell adhesion molecule.	Atrioventricular septal defect, partial, with heterotaxy syndrome {Atrioventricular septal defect, susceptibility to, 2}
			FANCD2	613 984	This gene encodes the protein for complementation group D2.	Fanconi anemia, complementation group D2
			BRK1	611 183	BRICK1, SCAR/WAVE actin-nucleating complex subunit	

			VHL	608 537	The protein encoded by this gene is a component of the protein complex that includes elongin B, elongin C, and cullin-2, and possesses ubiquitin ligase E3 activity.<br>This protein is involved in the ubiquitination and degradation of hypoxia-inducible-factor (HIF), which is a transcription factor that plays a central role in the regulation of gene expression by oxygen.	Erythrocytosis, familial, 2<br>Hemangioblastoma, cerebellar, somatic<br>Pheochromocytoma<br>Renal cell carcinoma, somatic<br>von Hippel-Lindau syndrome
			IRAK2	603 304	interleukin-1 receptor-associated kinase 2	
			GHRL	605 353	This gene encodes ghrelin-obestatin preproprotein, which generates ghrelin and obestatin.	{Obesity, susceptibility to}
			SEC13	600 152	The protein encoded by this gene belongs to the SEC13 family of WD-repeat proteins. It is a constituent of the endoplasmic reticulum and the nuclear pore complex.	

	[redacted]	[redacted]	ATP2B2	108 733	The protein encoded by this gene belongs to the family of P-type primary ion transport ATPases characterized by the formation of an aspartyl phosphate intermediate during the reaction cycle.	{Deafness, autosomal recessive 12, modifier of}
	[redacted]	[redacted]	SLC6A11	607 952	Gamma-aminobutyric acid (GABA) is a major inhibitory neurotransmitter.	
	[redacted]	[redacted]	SLC6A1	137 165	The SLC6A1 gene encodes a gamma-aminobutyric acid (GABA) transporter, which removes GABA from the synaptic cleft	
	[redacted]	[redacted]	HRH1	600 167	The protein encoded by this gene is an integral membrane protein and belongs to the G protein-coupled receptor superfamily.<br>It mediates the contraction of smooth muscles, the increase in capillary permeability due to contraction of terminal venules, the release of catecholamine from adrenal medulla,	

					and neurotransmission in the central nervous system.	
			ATG7	608 760	ATG7 autophagy related 7 homolog (S. cerevisiae)	
			SYN2	600 755	This gene is a member of the synapsin gene family. Synapsins encode neuronal phosphoproteins which associate with the cytoplasmic surface of synaptic vesicles.	{Schizophrenia, susceptibility to}
			TIMP4	601 915	This gene belongs to the TIMP gene family. The proteins encoded by this gene family are inhibitors of the matrix metalloproteinases, a group of peptidases involved in degradation of the extracellular matrix.	
			PPARG	601 487	This gene encodes a member of the peroxisome proliferator-activated receptor (PPAR) subfamily of nuclear receptors.	Carotid intimal medial thickness 1<br>Insulin resistance, severe, digenic<br>Lipodystrophy, familial partial, type 3

	[redacted]	[redacted]				Obesity, severe [Obesity, resistance to] {Diabetes, type 2} {Glioblastoma, susceptibility to}
	[redacted]	[redacted]	TSEN2	608 753	This gene encodes one of the subunits of the tRNA splicing endonuclease.	Pontocerebellar hypoplasia type 2B
	[redacted]	[redacted]	MKRN2	608 426	makorin ring finger protein 2	
	[redacted]	[redacted]	RAF1	164 760	This gene is the cellular homolog of viral raf gene (v-raf). The encoded protein is a MAP kinase kinase kinase (MAP3K), which functions downstream of the Ras family of membrane associated GTPases to which it binds directly.	
	[redacted]	[redacted]	CAND2	610 403	cullin-associated and neddylation-dissociated 2 (putative)	
	[redacted]	[redacted]	IQSEC1	610 166	IQ motif and Sec7 domain 1	
	[redacted]	[redacted]	NUP210	607 703	he protein encoded by this gene is a membrane-spanning glycoprotein that	

	[redacted]	[redacted]			is a major component of the nuclear pore complex.	
	[redacted]	[redacted]	HDAC11	607 226	This gene encodes a class IV histone deacetylase. The encoded protein is localized to the nucleus and may be involved in regulating the expression of interleukin 10.	
	[redacted]	[redacted]	FBLN2	135 821	This gene encodes an extracellular matrix protein, which belongs to the fibulin family. This protein binds various extracellular ligands and calcium.	
	[redacted]	[redacted]	WNT7A	601 570	This gene is a member of the WNT gene family, which consists of structurally related genes that encode secreted signaling proteins.	Fuhrmann syndrome<br>Ulna and fibula, absence of, with sever limb deficiency
	[redacted]	[redacted]	TPRXL	611 167	Homeobox genes encode DNA-binding proteins, many of which are thought to be involved in early embryonic development.	
	[redacted]	[redacted]	CHCHD4	611 077	coiled-coil-helix-coiled-coil-helix	

	[redacted]	[redacted]			domain containing 4	
	[redacted]	[redacted]	TMEM43	612 048	This gene contains a response element for PPAR gamma (an adipogenic transcription factor), which may explain the fibrofatty replacement of the myocardium, a characteristic pathological finding in ARVC.	Arrhythmogenic right ventricular dysplasia 5
	[redacted]	[redacted]	XPC	613 208	This gene encodes a component of the nucleotide excision repair (NER) pathway.	Xeroderma pigmentosum, group C
	[redacted]	[redacted]	LSM3	607 283	The Sm-like proteins are thought to form a stable heteromer present in tri-snRNP particles, which are important for pre-mRNA splicing.[	
	[redacted]	[redacted]	SLC6A6	186 854	Taurine (2-aminoethanesulfonic acid) is a major intracellular amino acid in mammals. It is involved in a number of important physiologic processes, including bile acid conjugation in hepatocytes, modulation of calcium	

					flux and neural excitability, osmoregulation, detoxification, and membrane stabilization.	
			NR2C2	601 426	Members of the nuclear hormone receptor family, such as NR2C2, act as ligand-activated transcription factors. The proteins have an N-terminal transactivation domain, a central DNA-binding domain with 2 zinc fingers, and a ligand-binding domain at the C terminus. The activated receptor/ligand complex is translocated to the nucleus where it binds to hormone response elements of target genes	
			MRPS25	611 987	This gene encodes a 28S subunit protein.	
			ZFYVE20	609 511	zinc finger, FYVE domain containing 20	
			DVWA	612 397	collagen, type VI, alpha 4	

					pseudogene 1	
			CAPN7	606 400	The function of the protein encoded by this gene is not known.	
			SH3BP5	605 612	SH3-domain binding protein 5 (BTK-associated)	
			EAF1	608 315	ELL associated factor 1	
			COLQ	603 033	This gene encodes the subunit of a collagen-like molecule associated with acetylcholinesterase in skeletal muscle.	Endplate acetylcholinesterase deficiency
			HACL1	604 300	2-hydroxyacyl-CoA lyase 1	
			BTD	609 019	Biotinidase functions to recycle biotin in the body by cleaving biocytin (biotin-epsilon-lysine),<br>a normal product of carboxylase degradation, resulting in regeneration of free biotin. Biotinidase has also been shown to have biotinyl-transferase activity.	Biotinidase deficiency
			ANKRD28	611 122	ankyrin repeat domain 28	
			DPH3	608 959	This gene encodes a CSL zinc finger-	

					containing protein that is required for dipthamide biosynthesis. The encoded protein is necessary for the initial step in the modification of a histidine residue in elongation factor-2 to diphthamide.	
			DAZL	601 486	The protein encoded by this gene is localized to the nucleus and cytoplasm of fetal germ cells and to the cytoplasm of developing oocytes. In the testis, this protein is localized to the nucleus of spermatogonia but relocates to the cytoplasm during meiosis where it persists in spermatids and spermatozoa. T	{Spermatogenic failure, susceptibility to}
			SATB1	602 075	This gene encodes a matrix protein which binds nuclear matrix and scaffold-associating DNAs through a unique nuclear architecture.	
			KCNH8	608 260	This gene encodes a member of the potassium channel, voltage-gated,	

	[redacted]	[redacted]			subfamily H.	
	[redacted]	[redacted]	RAB5A	179 512	RAB5A, member RAS oncogene family	
	[redacted]	[redacted]	KAT2B	602 303	The protein encoded by this gene associates with p300/CBP. It has in vitro and in vivo binding activity with CBP and p300,<br>and competes with E1A for binding sites in p300/CBP. It has histone acetyl transferase activity with core histones and nucleosome core particles, indicating that this protein plays a direct role in transcriptional regulation.	
	[redacted]	[redacted]	SGOL1	609 168	shugoshin-like 1 (S. pombe)	
	[redacted]	[redacted]	UBE2E2	602 163	ubiquitin-conjugating enzyme E2E 2	
	[redacted]	[redacted]	UBE2E1	602 916	This gene encodes a member of the E2 ubiquitin-conjugating enzyme family.	
	[redacted]	[redacted]	NKIRAS1	604 496	NFKB inhibitor interacting Ras-like 1	
	[redacted]	[redacted]	RPL15	604 174	This gene encodes a ribosomal	

					protein that is a component of the 60S subunit.	
			NR1D2	602 304	This gene encodes a member of the nuclear hormone receptor family, specifically the NR1 subfamily of receptors.<br>The encoded protein functions as a transcriptional repressor and may play a role in circadian rhythms and carbohydrate and lipid metabolism.	
			THRB	190 160	The protein encoded by this gene is a nuclear hormone receptor for triiodothyronine. I	Thyroid hormone resistance<br>Thyroid hormone resistance, autosomal recessive<br>Thyroid hormone resistance, selective pituitary
			RARB	180 220	This gene encodes retinoic acid receptor beta, a member of the thyroid-steroid hormone receptor superfamily of nuclear transcriptional regulators.	
			TOP2B	126431	This gene encodes a DNA	

					topoisomerase, an enzyme that controls and alters the topologic states of DNA during transcription.	
			NGLY1	610661	This gene encodes an enzyme that catalyzes hydrolysis of an N(4)-(acetyl-beta-D-glucosaminyl) asparagine residue to N-acetyl-beta-D-glucosaminylamine and a peptide containing an aspartate residue.	
			OXSM	610324	This gene encodes a beta-ketoacyl synthetase.	
			SLC4A7	603353	solute carrier family 4, sodium bicarbonate cotransporter, member 7	
			EOMES	604615	This gene encodes a member of a conserved protein family that shares a common DNA-binding domain, the T-box.	
			AZI2	609916	5-azacytidine induced 2	
			RBMS3	605786	This gene encodes an RNA-binding protein that belongs to the c-myc gene single-strand binding protein	

	[redacted]	[redacted]			family.	
	[redacted]	[redacted]	TGFBR2	190182	This gene encodes a member of the Ser/Thr protein kinase family and the TGFB receptor subfamily.	Colorectal cancer, hereditary nonpolyposis, type 6<br>Esophageal cancer, somatic<br>Loeys-Dietz syndrome, type 1B<br>Loeys-Dietz syndrome, type 2B
	[redacted]	[redacted]	STT3B	608605	STT3, subunit of the oligosaccharyltransferase complex, homolog B (S. cerevisiae)	
	[redacted]	[redacted]	OSBPL10	606738	This gene encodes a member of the oxysterol-binding protein (OSBP) family, a group of intracellular lipid receptors.	
	[redacted]	[redacted]	GPD1L	611778	The protein encoded by this gene catalyzes the conversion of sn-glycerol 3-phosphate to glycerone phosphate.	Brugada syndrome 2
	[redacted]	[redacted]	CMTM8	607891	This gene belongs to the chemokine-	

					like factor gene superfamily, a novel family that is similar to the chemokine and the transmembrane 4 superfamilies. This gene is one of several chemokine-like factor genes located in a cluster on chromosome 3.	
			CMTM7	607890	The protein encoded by this gene is highly expressed in leukocytes, but its exact function is unknown.	
			CMTM6	607889	This gene is widely expressed in many tissues, but the exact function of the encoded protein is unknown.	
			CCR4	604836	The protein encoded by this gene belongs to the G-protein-coupled receptor family . It is a receptor for the CC chemokine - MIP-1, RANTES, TARC and MCP-1.	
			GLB1	611458	This gene encodes beta-galactosidase-1, a lysosomal enzyme that hydrolyzes the terminal beta-	GM1-gangliosidosis, type I<br>GM1-gangliosidosis, type II<br>GM1-gangliosidosis, type III

	[redacted]	[redacted]			galactose from ganglioside substrates and other glycoconjugates.	Mucopolysaccharidosis type IVB (Morquio)
	[redacted]	[redacted]	CRTAP	605497	The protein encoded by this gene is similar to the chicken and mouse CRTAP genes. The encoded protein is a scaffolding protein that may influence the activity of at least one member of the cytohesin/ARNO family in response to specific cellular stimuli.	Osteogenesis imperfecta, type VII
	[redacted]	[redacted]	FBXL2	605652	This gene encodes a member of the F-box protein family which is characterized by an approximately 40 amino acid motif, the F-box.	
	[redacted]	[redacted]	UBP1	609784	upstream binding protein 1 (LBP-1a)	
	[redacted]	[redacted]	CLASP2	605853	cytoplasmic linker associated protein 2	
	[redacted]	[redacted]	PDCD6IP	608074	This gene encodes a protein that functions within the ESCRT pathway in the abscission stage of cytokinesis,	

					in intralumenal endosomal vesicle formation, and in enveloped virus budding.	
			ARPP-21	605488	The 21-kD cAMP-regulated phosphoprotein (ARPP21), also known as regulator of calmodulin (CaM; MIM 114180) signaling (RCS), plays a central role in integration of key neurotransmitter inputs into medium spiny neurons through its regulation of CaM-dependent kinase I (CaMKI) and protein phosphatase-2B (PP2B)	
			MIR128-2	611769	microRNAs (miRNAs) are short (20-24 nt) non-coding RNAs that are involved in post-transcriptional regulation of gene expression in multicellular organisms by affecting both the stability and translation of mRNAs.	
			STAC	602317	SH3 and cysteine rich domain	

	12q13.13	duplication				
			DIP2B	611379	This gene encodes a member of the disco-interacting protein homolog 2 protein family. The encoded protein contains a binding site for the transcriptional regulator DNA methyltransferase 1 associated protein 1 as well as AMP-binding sites.	Mental retardation, FRA12A type
	17p11.2	duplication				
			GRAP	604330	This gene encodes a member of the GRB2/Sem5/Drk family.	
	Xp22.33p22.2	deletion				
			ZBED1	300178	zinc finger, BED-type containing 1	
			CD99	313470	The protein encoded by this gene is a cell surface glycoprotein involved in leukocyte migration, T-cell adhesion, ganglioside GM1 and transmembrane protein transport, and T-cell death by a caspase-independent pathway.	
			XG	314700	This gene encodes the XG blood	[Blood group, XG system]

					group antigen, and is located at the pseudoautosomal boundary on the short (p) arm of chromosome X.	
			GYG2	300198	This gene encodes a member of the the glycogenin family.	
			ARSD	300002	The protein encoded by this gene is a member of the sulfatase family. Sulfatases are essential for the correct composition of bone and cartilage matrix.	
			ARSE	300180	Arylsulfatase E is a member of the sulfatase family. It is glycosylated postranslationally and localized to the golgi apparatus. Sulfatases are essential for the correct composition of bone and cartilage matrix.	Chondrodysplasia punctata, X-linked recessive
			ARSH	300586	Sulfatases, such as ARSH, hydrolyze sulfate esters from sulfated steroids, carbohydrates, proteoglycans, and glycolipids.	

			ARSF	300003	This gene is a member of the sulfatase family, and more specifically, the arylsulfatase subfamily. Members of the subfamily share similarity in sequence and splice sites, and are clustered together on chromosome X, suggesting that they are derived from recent gene duplication events. Sulfatases are essential for the correct composition of bone and cartilage matrix.	
			PRKX	300083	This gene encodes a serine threonine protein kinase that has similarity to the catalytic subunit of cyclic AMP dependent protein kinases.	
			NLGN4X	300427	This gene encodes a member of a family of neuronal cell surface proteins.	Mental retardation, X-linked {Asperger syndrome susceptibility, X-linked 2} {Autism susceptibility, X-

						linked 2}
			VCX3A	300533	VCX/Y genes encode small and highly charged proteins of unknown function.	
			HDHD1A	306480	This gene encodes a member of the haloacid dehalogenase-like (HAD) hydrolase superfamily.	
			STS	300747	The protein encoded by this gene catalyzes the conversion of sulfated steroid precursors to estrogens during pregnancy. The encoded protein is found in the endoplasmic reticulum, where it acts as a homodimer.	Ichthyosis, X-linked
			VCX	300229	VCX/Y genes encode small and highly charged proteins of unknown function.	
			PNPLA4	300102	This gene encodes a member of the patatin-like family of phospholipases.	
			VCX2	300532	VCX/Y genes encode small and	

					highly charged proteins of unknown function.	
			KAL1	300836	The encoded protein is similar in sequence to proteins known to function in neural cell adhesion and axonal migration.	Kallmann syndrome
			FAM9A	300477	This gene is a member of a gene family which arose through duplication on the X chromosome. The encoded protein may be a nuclear protein that is localized to the nucleolus, and has some similarity to a synaptonemal complex protein.	
			FAM9B	300478	his gene is a member of a gene family which arose through duplication on the X chromosome.	
			TBL1X	300196	The protein encoded by this gene has sequence similarity with members of the WD40 repeat-containing protein family.	
			GPR143	300808	This gene encodes a protein that	Nystagmus 6, congenital, X-

					binds to heterotrimeric G proteins and is targeted to melanosomes in pigment cells.	linked<br>Ocular albinism, type I, Nettleship-Falls type
**11**	**9q34.2q34.3**	**deletion**				
			POMT1	607423	The protein encoded by this gene is an O-mannosyltransferase that requires interaction with the product of the POMT2 gene for enzymatic function.	Muscular dystrophy-dystroglycanopathy (congenital with brain and eye anomalies), type A, 1<br>Muscular dystrophy-dystroglycanopathy (congenital with mental retardation), type B, 1<br>Muscular dystrophy-dystroglycanopathy (limb-girdle), type C, 1
			UCK1	609328	Uridine/cytidine kinase-1 (EC 2.7.1.48) is a pyrimidine ribonucleoside kinase that catalyzes the phosphorylation of uridine and cytidine to form uridine monophosphate (UMP) and cytidine	

					monophosphate (CMP)	
			RAPGEF1	600303	This gene encodes a human guanine nucleotide exchange factor.	
			MED27	605044	The protein encoded by this gene is a subunit of the CRSP (cofactor required for SP1 activation) complex, which, along with TFIID, is required for efficient activation by SP1.	
			SETX	608465	This gene encodes a protein named for its homology to the Sen1p protein of fungi which has RNA helicase activity encoded by a domain at the C-terminal end of the protein.	Amyotrophic lateral sclerosis 4, juvenile Ataxia-ocular apraxia-2
			TTF1	600 777	This gene encodes a transcription termination factor that is localized to the nucleolus and plays a critical role in ribosomal gene transcription.	
			BARHL1	605211	BarH-like homeobox 1	
			GTF3C4	604892	general transcription factor IIIC, polypeptide 4, 90kDa	

	16p13.3	deletion				
			FBXL16	609082	F-box and leucine-rich repeat protein 16	
			METRN	610998	Meteorin regulates glial cell differentiation and promotes the formation of axonal networks during neurogenesis	
			NARFL	611118	nuclear prelamin A recognition factor-like	
			MSLN	601051	This gene encodes a precursor protein that is cleaved into two products, megakaryocyte potentiating factor and mesothelin.	
			CHTF18	613201	CHTF18, CHTF8 (MIM 613202), and DCC1 (DSCC1; MIM 613203) are components of an alternative replication factor C (RFC) (see MIM 600404) complex that loads PCNA (MIM 176740) onto DNA during S phase of the cell cycle	

			GNG13	607298	Heterotrimeric G proteins, which consist of alpha (see MIM 139320), beta (see MIM 139380), and gamma subunits, function as signal transducers for the 7-transmembrane-helix G protein-coupled receptors.	
			LMF1	611761	The protein encoded by this gene resides in the endoplasmic reticulum, and is involved in the maturation and transport of lipoprotein lipase through the secretory pathway.	Lipase deficiency, combined
			SOX8	605923	This gene encodes a member of the SOX (SRY-related HMG-box) family of transcription factors involved in the regulation of embryonic development and in the determination of the cell fate.	
	21q22.3q22.3	deletion				
			UMODL1	613859	uromodulin-like 1	
			ABCG1	603076	The protein encoded by this gene is a	

					member of the superfamily of ATP-binding cassette (ABC) transporters.	
			TFF3	600633	Members of the trefoil family are characterized by having at least one copy of the trefoil motif, a 40-amino acid domain that contains three conserved disulfides. They are stable secretory proteins expressed in gastrointestinal mucosa.	
			TFF2	182590	Members of the trefoil family are characterized by having at least one copy of the trefoil motif, a 40-amino acid domain that contains three conserved disulfides. They are stable secretory proteins expressed in gastrointestinal mucosa.	
			TFF1	113710	Members of the trefoil family are characterized by having at least one copy of the trefoil motif, a 40-amino acid domain that contains three conserved disulfides. They are stable	

					secretory proteins expressed in gastrointestinal mucosa.	
			TMPRSS3	605511	This gene encodes a protein that belongs to the serine protease family.	Deafness, autosomal recessive 10, congenital Deafness, autosomal recessive 8, childhood onset
			UBASH3A	605736	This gene encodes one of two family members belonging to the T-cell ubiquitin ligand (TULA) family. Both family members can negatively regulate T-cell signaling.	
			RSPH1	609314	radial spoke head 1 homolog (Chlamydomonas)	
			SLC37A1	608094	solute carrier family 37 (glycerol-3-phosphate transporter), member 1	
			PDE9A	602973	The encoded protein plays a role in signal transduction by regulating the intracellular concentration of these cyclic nucleotides.	
			WDR4	605924	This gene encodes a member of the WD repeat protein family. WD	

					repeats are minimally conserved regions of approximately 40 amino acids typically bracketed by gly-his and trp-asp (GH-WD), which may facilitate formation of heterotrimeric or multiprotein complexes. Members of this family are involved in a variety of cellular processes, including cell cycle progression, signal transduction, apoptosis, and gene regulation.	
			NDUFV3	602184	The encoded protein is one of three proteins found in the flavoprotein fraction of the complex. The specific function of the encoded protein is unknown.	
			PKNOX1	602100	PBX/knotted 1 homeobox 1	
			CBS	613381	The protein encoded by this gene acts as a homotetramer to catalyze the conversion of homocysteine to cystathionine, the first step in the	Homocystinuria, B6-responsive and nonresponsive types Thrombosis,

					transsulfuration pathway.	hyperhomocysteinemic
			U2AF1	191317	This gene encodes the small subunit which plays a critical role in both constitutive and enhancer-dependent RNA splicing by directly mediating interactions between the large subunit and proteins bound to the enhancers	
			CRYAA	123580	Alpha crystallins are composed of two gene products: alpha-A and alpha-B, for acidic and basic, respectively. Alpha crystallins can be induced by heat shock and are members of the small heat shock protein (sHSP also known as the HSP20) family. They act as molecular chaperones although they do not renature proteins and release them in the fashion of a true chaperone; instead they hold them in large soluble aggregates.	Cataract, autosomal dominant nuclear Cataract, congenital, autosomal recessive Cataract, zonular central nuclear, autosomal dominant

			SIK1	605705	salt-inducible kinase 1	
			HSF2BP	604554	heat shock transcription factor 2 binding protein	
			RRP1B	610654	ribosomal RNA processing 1 homolog B (S. cerevisiae)	
			PDXK	179020	The protein encoded by this gene phosphorylates vitamin B6, a step required for the conversion of vitamin B6 to pyridoxal-5-phosphate, an important cofactor in intermediary metabolism. The encoded protein is cytoplasmic and probably acts as a homodimer.	
			CSTB	601145	This gene encodes a stefin that functions as an intracellular thiol protease inhibitor.	Epilepsy, progressive myoclonic 1A (Unverricht and Lundborg)
			RRP1	610653	The protein encoded by this gene is the putative homolog of the yeast ribosomal RNA processing protein RRP1. The encoded protein is involved in the late stages of	

					nucleologenesis at the end of mitosis, and may be required for the generation of 28S rRNA.	
**12**	**3p22.1p21.32**	**deletion**				
			CCK	118440	Cholecystokinin is a brain/gut peptide. In the gut, it induces the release of pancreatic enzymes and the contraction of the gallbladder. In the brain, its physiologic role is unclear.	
			LYZL4	612750	Lysozymes (see LYZ; MIM 153450), especially C-type lysozymes, are well-recognized bacteriolytic factors widely distributed in the animal kingdom and play a mainly protective role in host defense.	
			VIPR1	192321	his gene encodes a receptor for vasoactive intestinal peptide, a small neuropeptide.	
			SEC22C	604028	This gene encodes a member of the SEC22 family of vesicle trafficking	

					proteins. The encoded protein is localized to the endoplasmic reticulum and may play a role in the early stages of ER-Golgi protein trafficking.	
			SS18L2	606473	Synovial sarcomas occur most frequently in the extremities around large joints.	
			NKTR	161565	This gene encodes a membrane-anchored protein with a hydrophobic amino terminal domain and a cyclophilin-like PPIase domain.	
			HHATL	608116	hedgehog acyltransferase-like	
			CCBP2	602648	This gene encodes a beta chemokine receptor, which is predicted to be a seven transmembrane protein similar to G protein-coupled receptors.	
			CYP8B1	602172	This gene encodes a member of the cytochrome P450 superfamily of enzymes.	
			SNRK	612760	SNF related kinase	

			ANO10	613726	The transmembrane protein encoded by this gene is a member of a family of calcium-activated chloride channels.	Spinocerebellar ataxia, autosomal recessive 10
			ABHD5	604780	The protein encoded by this gene belongs to a large family of proteins defined by an alpha/beta hydrolase fold, and contains three sequence motifs that correspond to a catalytic triad found in the esterase/lipase/thioesterase subfamily.	Chanarin-Dorfman syndrome
			MIR138-1	613394	microRNAs (miRNAs) are short (20-24 nt) non-coding RNAs that are involved in post-transcriptional regulation of gene expression in multicellular organisms by affecting both the stability and translation of mRNAs.	
			ZNF35	194533	zinc finger protein 3	

**13**	**16p13.3**	**duplication**				
			SOLH	603267	This gene encodes a protein containing zinc-finger-like repeats and a calpain-like protease domain.	
			PIGQ	605754	This gene is involved in the first step in glycosylphosphatidylinositol (GPI)-anchor biosynthesis.	
			WFIKKN1	608021	This gene encodes a secreted multidomain protein consisting of a signal peptide, a WAP domain, a follistatin domain, an immunoglobulin domain, two tandem Kunitz domains, and an NTR domain.	
			RHOT2	613889	This gene encodes a member of the Rho family of GTPases.	
			RHBDL1	603264	rhomboid, veinlet-like 1 (Drosophila)	
			STUB1	607207	STUB1, or CHIP, is a ubiquitin ligase/cochaperone that participates in protein quality control by targeting a broad range of chaperone protein substrates for degradation	

			FBXL16	609082	F-box and leucine-rich repeat protein 16	
			METRN	610998	Meteorin regulates glial cell differentiation and promotes the formation of axonal networks during neurogenesis	
	Xp11.23	duplication				
			ZNF81	314998	This gene encodes a protein that likely functions as a transcription factor.	Mental retardation, X-linked 45
			ZNF182	314993	Zinc-finger proteins bind nucleic acids and play important roles in various cellular functions, including cell proliferation, differentiation, and apoptosis.	
**15**	**10q25.2qter**	**duplication**				
			DUSP5	603069	This gene product inactivates ERK1, is expressed in a variety of tissues with the highest levels in pancreas and brain, and is localized in the	

	[redacted]	[redacted]			nucleus.	
	[redacted]	[redacted]	SMC3	606062	The encoded protein occurs in certain cell types as either an intracellular, nuclear protein or a secreted protein.	Cornelia de Lange syndrome 3
	[redacted]	[redacted]	RBM20	613171	This gene encodes a protein that likely binds RNA	Cardiomyopathy, dilated, 1DD
	[redacted]	[redacted]	PDCD4	608610	This gene is a tumor suppressor and encodes a protein that binds to the eukaryotic translation initiation factor 4A1 and inhibits its function by preventing RNA binding.	
	[redacted]	[redacted]	BBIP1	613605	BBSome interacting protein 1	
	[redacted]	[redacted]	SHOC2	602775	soc-2 suppressor of clear homolog (C. elegans)	Noonan-like syndrome with loose anagen hair
	[redacted]	[redacted]	ADRA2A	104210	This gene encodes alpha2A subtype and it contains no introns in either its coding or untranslated sequences.	
	[redacted]	[redacted]	GPAM	602395	This gene encodes a mitochondrial enzyme which prefers saturated fatty acids as its substrate for the synthesis of glycerolipids.	

	[redacted]	[redacted]	TECTB	602653	The genes for alpha-tectorin (MIM 602574) and beta-tectorin encode the major noncollagenous proteins of the tectorial membrane of the cochlea.	
	[redacted]	[redacted]	ACSL5	605677	The protein encoded by this gene is an isozyme of the long-chain fatty-acid-coenzyme A ligase family.	
	[redacted]	[redacted]	TCF7L2	602228	transcription factor 7-like 2 (T-cell specific, HMG-box)	{Diabetes mellitus, type 2, susceptibility to}
	[redacted]	[redacted]	HABP2	603924	The protein encoded by this gene is an extracellular serine protease that binds hyaluronic acid and is involved in cell adhesion.	{Carotid stenosis, susceptibility to} {Venous thromboembolism, susceptibility to}
	[redacted]	[redacted]	NRAP	602873	nebulin-related anchoring protein	
	[redacted]	[redacted]	CASP7	601761	This gene encodes a protein which is a member of the cysteine-aspartic acid protease (caspase) family.	
	[redacted]	[redacted]	DCLRE1A	609682	DNA cross-link repair 1A	
	[redacted]	[redacted]	ADRB1	109630	Specific polymorphisms in this gene have been shown to affect the resting heart rate and can be involved in	[Resting heart rate] 607276 {Congestive heart failure and beta-blocker response,

	[redacted]	[redacted]			heart failure.	modifier of}
	[redacted]	[redacted]	TDRD1	605796	This gene is similar to a mouse gene that encodes a tudor domain protein.	
	[redacted]	[redacted]	AFAP1L2	612420	actin filament associated protein 1-like 2	
	[redacted]	[redacted]	ABLIM1	602330	This gene encodes a cytoskeletal LIM protein that binds to actin filaments via a domain that is homologous to erythrocyte dematin.	
	[redacted]	[redacted]	TRUB1	610726	TruB pseudouridine (psi) synthase homolog 1 (E. coli)	
	[redacted]	[redacted]	ATRNL1	612869	attractin-like 1	
	[redacted]	[redacted]	GFRA1	601496	The protein encoded by this gene is a member of the GDNF receptor family. It is a glycosylphosphatidylinositol(GPI)-linked cell surface receptor for both GDNF and NTN, and mediates activation of the RET tyrosine kinase receptor.	

			PNLIP	246600	This gene is a member of the lipase gene family. It encodes a carboxyl esterase that hydrolyzes insoluble, emulsified triglycerides, and is essential for the efficient digestion of dietary fats.	Pancreatic lipase deficiency
			PNLIPRP1	604422	pancreatic lipase-related protein 1	
			PNLIPRP2	604423	pancreatic lipase-related protein 2	
			HSPA12A	610701	heat shock 70kDa protein 12A	
			KIAA1598	611171	KIAA1598	
			VAX1	604294	This gene encodes a homeo-domain containing protein from a class of homeobox transcription factors which are conserved in vertebrates.	
			KCNK18	613655	This gene encodes a member of the superfamily of potassium channel proteins containing two pore-forming P domains and the encoded protein functions as an outward rectifying potassium channel.	{Migraine, with or without aura, susceptibility to, 13}

			SLC18A2	193001	solute carrier family 18 (vesicular monoamine), member 2	
			PDZD8	614235	PDZ domain containing 8	
			EMX2OS	607637	EMX2 opposite strand/antisense RNA (non-protein coding)	
			EMX2	600035	This gene encodes a homeobox-containing transcription factor that is the homolog to the 'empty spiracles' gene in Drosophila.	Schizencephaly
			RAB11FIP2	608599	RAB11 family interacting protein 2 (class I)	
			CASC2	608598	cancer susceptibility candidate 2 (non-protein coding)	
			PRLHR	600895	prolactin releasing hormone receptor	
			NANOS1	608226	nanos homolog 1 (Drosophila)	
			EIF3A	602039	eukaryotic translation initiation factor 3, subunit A	
			PRDX3	604769	This gene encodes a protein with antioxidant function and is localized in the mitochondrion.	

			GRK5	600870	This gene encodes a member of the guanine nucleotide-binding protein (G protein)-coupled receptor kinase subfamily of the Ser/Thr protein kinase family.	
			RGS10	602856	This protein associates specifically with the activated forms of the two related G-protein subunits, G-alphai3 and G-alphaz but fails to interact with the structurally and functionally distinct G-alpha subunits.	
			TIAL1	603413	The protein encoded by this gene is a member of a family of RNA-binding proteins, has three RNA recognition motifs (RRMs), and binds adenine and uridine-rich elements in mRNA and pre-mRNAs of a wide range of genes.	
			BAG3	603883	The protein encoded by this gene contains a WW domain in the N-terminal region and a BAG domain in	Cardiomyopathy, dilated, 1HH<br>Myopathy, myofibrillar,

					the C-terminal region.<br>The BAG domains of BAG1, BAG2, and BAG3 interact specifically with the Hsc70 ATPase domain in vitro and in mammalian cells.	BAG3-related
			INPP5F	609389	The protein encoded by this gene is an inositol 1,4,5-trisphosphate (InsP3) 5-phosphatase and contains a Sac domain.	
			C10orf119	610909	This gene encodes a protein which is a component of the hexameric minichromosome maintenance (MCM) complex which regulates initiation and elongation of DNA.	
			WDR11	606417	This gene encodes a member of the WD repeat protein family. WD repeats are minimally conserved regions of approximately 40 amino acids typically bracketed by gly-his and trp-asp (GH-WD), which may facilitate formation of heterotrimeric or	

					multiprotein complexes.	
			FGFR2	176943	The protein encoded by this gene is a member of the fibroblast growth factor receptor family, where amino acid sequence is highly conserved between members and throughout evolution.	Antley-Bixler syndrome without genital anomalies or disordered steroidogenesis<br>Apert syndrome<br>Beare-Stevenson cutis gyrata syndrome<br>Craniofacial-skeletal-dermatologic dysplasia<br>Craniosynostosis, nonspecific<br>Crouzon syndrome<br>Gastric cancer, somatic<br>Jackson-Weiss syndrome<br>LADD syndrome<br>Pfeiffer syndrome<br>Saethre-Chotzen syndrome<br>Scaphocephaly and Axenfeld-Rieger anomaly<br>Scaphocephaly, maxillary retrusion, and mental

						retardation
			ATE1	607103	This gene encodes an arginyltransferase, an enzyme that is involved in posttranslational conjugation of arginine to N-terminal aspartate or glutamate residues.	
			NSMCE4A	612987	non-SMC element 4 homolog A (S. cerevisiae)	
			TACC2	605302	This gene encodes a protein that concentrates at centrosomes throughout the cell cycle.	
			PLEKHA1	607772	This gene encodes a pleckstrin homology domain-containing adapter protein.	{Age-related maculopathy, susceptibility to}

			ARMS2	611313	his gene encodes a protein that is thought to play a role in diseases in the elderly.	{Macular degeneration, age-related, 8}
			HTRA1	602194	This gene encodes a member of the trypsin family of serine proteases.	{Macular degeneration, age-related, 7}<br>{Macular degeneration, age-related, neovascular type}
			DMBT1	601969	Loss of sequences from human chromosome 10q has been associated with the progression of human cancers.	
			PSTK	611310	phosphoseryl-tRNA kinase	
			IKZF5	606238	IKAROS family zinc finger 5 (Pegasus)	
			ACADSB	600301	Short/branched chain acyl-CoA dehydrogenase(ACADSB) is a member of the acyl-CoA dehydrogenase family of enzymes that catalyze the dehydrogenation of acyl-CoA derivatives in the metabolism of fatty acids or branch	2-methylbutyrylglycinuria

					chained amino acids. S	
			HMX3	613380	H6 family homeobox 3	
			HMX2	600647	H6 family homeobox 2	
			BUB3	603719	his gene encodes a protein involved in spindle checkpoint function.	
			GPR26	604847	G protein-coupled receptor 26	
			CHST15	608277	carbohydrate (N-acetylgalactosamine 4-sulfate 6-O) sulfotransferase 15	
			OAT	613349	This gene encodes the mitochondrial enzyme ornithine aminotransferase, which is a key enzyme in the pathway that converts arginine and ornithine into the major excitatory and inhibitory neurotransmitters glutamate and GABA.	Gyrate atrophy of choroid and retina with or without ornithinemia
			FAM175B	611144	family with sequence similarity 175, member B	
			ZRANB1	611749	zinc finger, RAN-binding domain containing 1	
			CTBP2	602619	This gene produces alternative transcripts encoding two distinct	

					proteins. One protein is a transcriptional repressor, while the other isoform is a major component of specialized synapses known as synaptic ribbons.	
			MMP21	608416	This gene encodes a member of the matrix metalloproteinase family.	
			UROS	606938	The protein encoded by this gene catalyzes the fourth step of porphyrin biosynthesis in the heme biosynthetic pathway.	Porphyria, congenital erythropoietic
			BCCIP	611883	This gene product was isolated on the basis of its interaction with BRCA2 and p21 proteins.	
			DHX32	607960	DEAD box proteins, characterized by the conserved motif Asp-Glu-Ala-Asp (DEAD), are putative RNA helicases. They are implicated in a number of cellular processes involving alteration of RNA secondary structure such as translation initiation, nuclear and	

					mitochondrial splicing, and ribosome and spliceosome assembly.	
			FANK1	611640	fibronectin type III and ankyrin repeat domains 1	
			ADAM12	602714	This gene encodes a member of the ADAM (a disintegrin and metalloprotease) protein family.	
			DOCK1	601403	dedicator of cytokinesis 1	
			NPS	609513	neuropeptide S	
			PTPRE	600926	The protein encoded by this gene is a member of the protein tyrosine phosphatase (PTP) family.	
			MKI67	176741	This gene encodes a nuclear protein that is associated with and may be necessary for cellular proliferation.	
			MGMT	156569	O-6-methylguanine-DNA methyltransferase	
			EBF3	607407	This gene encodes a member of the early B-cell factor (EBF) family of DNA binding transcription factors.	
			GLRX3	612754	This gene encodes a member of the	

					glutaredoxin family.	
			PPP2R2D	613992	protein phosphatase 2, regulatory subunit B, delta	
			BNIP3	603293	his gene is a member of the BCL2/adenovirus E1B 19 kd-interacting protein (BNIP) family.	
			JAKMIP3	611198	Janus kinase and microtubule interacting protein 3	
			DPYSL4	608407	dihydropyrimidinase-like 4	
			INPP5A	600106	The protein encoded by this gene is a membrane-associated type I inositol 1,4,5-trisphosphate (InsP3) 5-phosphatase.	
			NKX6-2	605955	NK6 homeobox 2	
			GPR123	612302	G protein-coupled receptor 123	
			UTF1	604130	undifferentiated embryonic cell transcription factor 1	
			VENTX	607158	This gene encodes a member of the Vent family of homeodomain proteins. The encoded protein may function as a transcriptional repressor and be	

					involved in mesodermal patterning and hemopoietic stem cell maintenance.	
			ADAM8	602267	This gene encodes a member of the ADAM (a disintegrin and metalloprotease domain) family.	
			CALY	604647	The protein encoded by this gene is a type II single transmembrane protein.	
			PRAP1	609776	proline-rich acidic protein 1	
			ECHS1	602292	The protein encoded by this gene functions in the second step of the mitochondrial fatty acid beta-oxidation pathway.	
	13q33.3qter	deletion				
			LIG4	601837	The protein encoded by this gene is a DNA ligase that joins single-strand breaks in a double-stranded polydeoxynucleotide in an ATP-dependent reaction.	LIG4 syndrome<br>Severe combined immunodeficiency with sensitivity to ionizing radiation<br>{Multiple myeloma, resistance to}

			TNFSF13B	603969	The protein encoded by this gene is a cytokine that belongs to the tumor necrosis factor (TNF) ligand family.	
			IRS2	600797	This gene encodes the insulin receptor substrate 2, a cytoplasmic signaling molecule that mediates effects of insulin, insulin-like growth factor 1, and other cytokines by acting as a molecular adaptor between diverse receptor tyrosine kinases and downstream effectors.	{Diabetes mellitus, noninsulin-dependent}
			COL4A1	120130	This gene encodes the major type IV alpha collagen chain of basement membranes.	Angiopathy, hereditary, with nephropathy, aneurysms, and muscle<br>Brain small vessel disease with Axenfeld-Rieger anomaly<br>Brain small vessel disease with hemorrhage<br>Porencephaly 1
			COL4A2	120090	This gene encodes one of the six	13q34 Porencephaly 2

					subunits of type IV collagen, the major structural component of basement membranes.	{Stroke, hemorrhagic}
			CARS2	612800	cysteinyl-tRNA synthetase 2, mitochondrial (putative)	
			ING1	601566	This gene encodes a tumor suppressor protein that can induce cell growth arrest and apoptosis.	Squamous cell carcinoma, head and neck, somatic
			ARHGEF7	605477	The encoded protein belongs to a family of cytoplasmic proteins that activate the Ras-like family of Rho proteins by exchanging bound GDP for GTP.	
			SOX1	602148	This intronless gene encodes a member of the SOX (SRY-related HMG-box) family of transcription factors involved in the regulation of embryonic development and in the determination of the cell fate.	
			ATP11A	605868	The protein encoded by this gene is an integral membrane ATPase.	

			MCF2L	609499	MCF.2 cell line derived transforming sequence-like	
			F7	613878	This gene encodes coagulation factor VII which is a vitamin K-dependent factor essential for hemostasis.	Factor VII deficiency {Myocardial infarction, decreased susceptibility to}
			F10	613872	This gene encodes the vitamin K-dependent coagulation factor X of the blood coagulation cascade.	Factor X deficiency
			PROZ	176895	This gene encodes a liver vitamin K-dependent glycoprotein that is synthesized in the liver and secreted into the plasma.	[Protein Z deficiency]
			PCID2	613713	PCI domain containing 2	
			CUL4A	603137	cullin 4A	
			LAMP1	153330	The protein encoded by this gene is a member of a family of membrane glycoproteins.	
			ADPRHL1	610620	ADP-ribosylhydrolase like 1	
**17**	**13q32.2**	**deletion**				
			IPO5	602008	The protein encoded by this gene is a member of the importin beta family.	

	16p13.3	duplication				
			PKD1	601313	This gene encodes a member of the polycystin protein family.	Polycystic kidney disease, adult type I
			RAB26	605455	Members of the RAB protein family, including RAB26, are important regulators of vesicular fusion and trafficking.	
			TRAF7	606692	Tumor necrosis factor (TNF; see MIM 191160) receptor-associated factors, such as TRAF7, are signal transducers for members of the TNF receptor superfamily (see MIM 191190).	
			CASKIN1	612184	CASK interacting protein 1	
			MLST8	612190	MTOR associated protein, LST8 homolog (S. cerevisiae)	
			PGP	172280	phosphoglycolate phosphatase	
			E4F1	603022	The zinc finger protein encoded by this gene is one of several cellular transcription factors whose DNA-binding activities are regulated	

					through the action of adenovirus E1A.	
			DNASE1L2	602622	deoxyribonuclease I-like 2	
			DCI	600305	This gene encodes a member of the hydratase/isomerase superfamily. The protein encoded is a key mitochondrial enzyme involved in beta-oxidation of unsaturated fatty acids.	
			RNPS1	606447	This gene encodes a protein that is part of a post-splicing multiprotein complex involved in both mRNA nuclear export and mRNA surveillance.	
			ABCA3	601615	The membrane-associated protein encoded by this gene is a member of the superfamily of ATP-binding cassette (ABC) transporters.	Surfactant metabolism dysfunction, pulmonary, 3
**18**	**19p13.3**	**duplication**				
			THEG	609503	theg spermatid protein	
			C2CD4C	610336	C2 calcium-dependent domain containing 4C	

			SHC2	605217	SHC (Src homology 2 domain containing) transforming protein 2	
			MADCAM1	102670	The protein encoded by this gene is an endothelial cell adhesion molecule that interacts preferentially with the leukocyte beta7 integrin LPAM-1 (alpha4beta7), L-selectin, and VLA-4 (alpha4beta1) on myeloid cells to direct leukocytes into mucosal and inflamed tissues.	
			CDC34	116948	The protein encoded by this gene is a member of the ubiquitin-conjugating enzyme family. Ubiquitin-conjugating enzyme catalyzes the covalent attachment of ubiquitin to other proteins.	
			GZMM	600311	Human natural killer (NK) cells and activated lymphocytes express and store a distinct subset of neutral serine proteases together with proteoglycans and other immune	

	[redacted]	[redacted]			effector molecules in large cytoplasmic granules.	
	[redacted]	[redacted]	BSG	109480	The protein encoded by this gene is a plasma membrane protein that is important in spermatogenesis, embryo implantation, neural network formation, and tumor progression.	[Blood group, OK]
	[redacted]	[redacted]	HCN2	602781	hyperpolarization activated cyclic nucleotide-gated potassium channel 2	
	[redacted]	[redacted]	POLRMT	601778	This gene encodes a mitochondrial DNA-directed RNA polymerase.	
	[redacted]	[redacted]	FGF22	605831	The protein encoded by this gene is a member of the fibroblast growth factor (FGF) family.	
	[redacted]	[redacted]	FSTL3	605343	follistatin-like 3 (secreted glycoprotein)	
	[redacted]	[redacted]	PALM	608134	This gene encodes a member of the paralemmin protein family.	
	[redacted]	[redacted]	PTBP1	600693	This gene belongs to the subfamily of ubiquitously expressed	

	[redacted]	[redacted]			heterogeneous nuclear ribonucleoproteins (hnRNPs).	
	[redacted]	[redacted]	LPPR3	610391	The proteins in the lipid phosphate phosphatase (LPP) family, including PRG2, are integral membrane proteins that modulate bioactive lipid phosphates including phosphatidate, lysophosphatidate, and sphingosine-1-phosphate in the context of cell migration, neurite retraction, and mitogenesis	
	[redacted]	[redacted]	AZU1	162815	The protein encoded by this gene is an azurophil granule antibiotic protein, with monocyte chemotactic and antibacterial activity. It is also an important multifunctional inflammatory mediator.	
	[redacted]	[redacted]	PRTN3	177020	proteinase 3	
	[redacted]	[redacted]	ELANE	134350	Elastases form a subfamily of serine proteases that hydrolyze many proteins in addition to elastin.	Complement factor D deficiency

			CFD	604161	The protein encoded by this gene is a member of the trypsin family of peptidases. The encoded protein is a component of the alternative complement pathway best known for its role in humoral suppression of infectious agents.	Hypogonadotropic hypogonadism Precocious puberty, central
			KISS1R	604161	The protein encoded by this gene is a galanin-like G protein-coupled receptor that binds metastin, a peptide encoded by the metastasis suppressor gene KISS1.	
			ARID3A	603265	This gene encodes a member of the ARID (AT-rich interaction domain) family of DNA binding proteins.	
			GAMT	601240	The protein encoded by this gene is a methyltransferase that converts guanidoacetate to creatine, using S-adenosylmethionine as the methyl donor.	
			DAZAP1	607430	This gene encodes a RNA-binding	GAMT deficiency

					protein with two RNP motifs that was originally identified by its interaction with the infertility factors DAZ and DAZL	
			RPS15	180535	This gene encodes a ribosomal protein that is a component of the 40S subunit.	
			APC2	612034	adenomatosis polyposis coli 2	
			PCSK4	600487	This gene encodes a member of the subtilisin-like proprotein convertase family.	
			REEP6	609346	receptor accessory protein 6	
			MEX3D	611009	mex-3 homolog D (C. elegans)	
			MBD3	603573	methyl-CpG binding domain protein 3	
			UQCR11	609711	This gene encodes the smallest known component of the ubiquinol-cytochrome c reductase complex, which forms part of the mitochondrial respiratory chain. The encoded protein may function as a binding factor for the iron-sulfur protein in this	

					complex.	
			TCF3	147141	This gene encodes a member of the E protein (class I) family of helix-loop-helix transcription factors.	Leukemia, acute lymphoblastic
			ONECUT3	611294	one cut homeobox 3	
			ATP8B3	605866	The protein encoded by this gene belongs to the family of P-type cation transport ATPases, and to the subfamily of aminophospholipid-transporting ATPases.	
			REXO1	609614	REX1, RNA exonuclease 1 homolog (S. cerevisiae)	
			KLF16	606139	Kruppel-like factor 16	
			SCAMP4	613764	secretory carrier membrane protein 4	
			CSNK1G2	602214	casein kinase 1, gamma 2	
			BTBD2	608531	BTB (POZ) domain containing 2	
			MKNK2	605069	This gene encodes a member of the calcium/calmodulin-dependent protein kinases (CAMK) Ser/Thr protein kinase family, which belongs to the protein kinase superfamily.	

	[redacted]	[redacted]	AP3D1	607246	The protein encoded by this gene is a subunit of the AP3 adaptor-like complex, which is not clathrin-associated, but is associated with the golgi region, as well as more peripheral structures.	
	[redacted]	[redacted]	DOT1L	607375	The protein encoded by this gene is a histone methyltransferase that methylates lysine-79 of histone H3.	
	[redacted]	[redacted]	SF3A2	600796	This gene encodes subunit 2 of the splicing factor 3a protein complex.	
	[redacted]	[redacted]	AMH	600957	Anti-Mullerian hormone is a member of the transforming growth factor-beta gene family which mediates male sexual differentiation.	Persistent Mullerian duct syndrome, type I
	[redacted]	[redacted]	JSRP1	608743	he sarcoplasmic reticulum (SR) is an intracellular membrane compartment that controls intracellular calcium concentration and therefore plays a role in excitation-contraction coupling.	
	[redacted]	[redacted]	OAZ1	601579	The antizyme encoded by this gene	

					inhibits ornithine decarboxylase and accelerates its degradation.	
			LINGO3	609792	leucine rich repeat and Ig domain containing 3	
			LSM7	607287	LSM7 homolog, U6 small nuclear RNA associated (S. cerevisiae)	
			SPPL2B	608239	This gene encodes a member of the GXGD family of aspartic proteases.	
			TMPRSS9	610477	transmembrane protease, serine 9	
			TIMM13	607383	This gene encodes a translocase with similarity to yeast mitochondrial proteins that are involved in the import of metabolite transporters from the cytoplasm and into the mitochondrial inner membrane.	
			LMNB2	150341	The nuclear lamina consists of a two-dimensional matrix of proteins located next to the inner nuclear membrane.	{Lipodystrophy, partial, acquired, susceptibility to}
			GADD45B	604948	This gene is a member of a group of genes whose transcript levels are increased following stressful growth	

					arrest conditions and treatment with DNA-damaging agents. The genes in this group respond to environmental stresses by mediating activation of the p38/JNK pathway.	
			GNG7	604430	guanine nucleotide binding protein (G protein), gamma 7	
			DIRAS1	607862	DIRAS family, GTP-binding RAS-like 1	
			SLC39A3	612168	solute carrier family 39 (zinc transporter), member 3	
			SGTA	603419	This gene encodes a protein which is capable of interacting with the major nonstructural protein of parvovirus H-1 and 70-kDa heat shock cognate protein; however, its function is not known.	
			THOP1	601117	thimet oligopeptidase 1	
			ZNF77	194551	zinc finger protein 77	
			TLE6	612399	transducin-like enhancer of split 6 (E(sp1) homolog, Drosophila)	

			TLE2	601041	transducin-like enhancer of split 2 (E(sp1) homolog, Drosophila)	
			AES	600188	The encoded protein, which belongs to the groucho/TLE family of proteins, can function as a homooligomer or as a heteroologimer with other family members to dominantly repress the expression of other family member genes.	
			GNA11	139313	guanine nucleotide binding protein (G protein), alpha 11 (Gq class)	
			GNA15	139314	guanine nucleotide binding protein (G protein), alpha 15 (Gq class)	
			S1PR4	603751	This gene is a member of the endothelial differentiation, G-protein-coupled (EDG)) receptor gene family. EDG receptors bind lysophospholipids or lysosphingolipids as ligands, and are involved in cell signalling in many different cell types.	

	[redacted]	[redacted]	NCLN	609156	nicalin	
	[redacted]	[redacted]	CELF5	612680	This gene encodes a member of the the CELF/BRUNOL protein family, which contain two N-terminal RNA recognition motif (RRM) domains, one C-terminal RRM domain, and a divergent segment of 160-230 aa between the second and third RRM domains.	
	[redacted]	[redacted]	NFIC	600729	The protein encoded by this gene belongs to the CTF/NF-I family.	
	[redacted]	[redacted]	DOHH	611262	This gene encodes a metalloenzyme that catalyzes the last step in the conversion of lysine to the unique amino acid hypusine in eukaryotic initiation factor 5A.	
	[redacted]	[redacted]	FZR1	603619	fizzy/cell division cycle 20 related 1 (Drosophila)	
	[redacted]	[redacted]	HMG20B	605535	high mobility group 20B	
	[redacted]	[redacted]	GIPC3	608792	The protein encoded by this gene belongs to the GIPC family.	Deafness, autosomal recessive 15

			TBXA2R	188070	This gene encodes a member of the G protein-coupled receptor family.	{Bleeding disorder, platelet-type, 13, susceptibility to}
			PIP5K1C	606102	This locus encodes a type I phosphatidylinositol 4-phosphate 5-kinase.	Lethal congenital contractural syndrome 3
			TJP3	612689	tight junction protein 3 (zona occludens 3)	
			APBA3	604262	The protein encoded by this gene is a member of the X11 protein family	
			MRPL54	611858	This gene encodes a 39S subunit protein.	
			RAX2	610362	This gene encodes a homeodomain-containing protein that plays a role in eye development.	Cone-rod dystrophy 11 Macular degeneration, age-related, 6
			MATK	600038	The protein encoded by this gene has amino acid sequence similarity to Csk tyrosine kinase and has the structural features of the CSK subfamily: SRC homology SH2 and SH3 domains, a catalytic domain, a unique N terminus, lack of	

					myristylation signals, lack of a negative regulatory phosphorylation site, and lack of an autophosphorylation site. This protein is thought to play a significant role in the signal transduction of hematopoietic cells.	
			ATCAY	608179	This gene encodes a neuron-restricted protein that contains a CRAL-TRIO motif common to proteins that bind small lipophilic molecules.	Ataxia, cerebellar, Cayman type
			ITGB1BP3	608705	integrin beta 1 binding protein 3	
			DAPK3	603289	Death-associated protein kinase 3 (DAPK3) induces morphological changes in apoptosis when overexpressed in mammalian cells.	
			EEF2	130610	This gene encodes a member of the GTP-binding translation elongation factor family.	
			PIAS4	605989	protein inhibitor of activated STAT, 4	

			ZBTB7A	605878	zinc finger and BTB domain containing 7A	
			MAP2K2	601263	The protein encoded by this gene is a dual specificity protein kinase that belongs to the MAP kinase kinase family.	Cardiofaciocutaneous syndrome
			CREB3L3	611998	cAMP responsive element binding protein 3-like 3	
			SIRT6	606211	This gene encodes a member of the sirtuin family of proteins, homologs to the yeast Sir2 protein.	
			EBI3	605816	This gene was identified by its induced expression in B lymphocytes in response Epstein-Barr virus infection.	
			SHD	610481	Src homology 2 domain containing transforming protein D	
			FSD1	609828	This gene encodes a centrosome associated protein that is characterized by an N-terminal coiled-coil region downstream of B-	

					box (BBC) domain, a central fibronectin type III domain, and a C-terminal repeats in splA and RyR (SPRY) domain.	
			STAP2	607881	This gene encodes the substrate of breast tumor kinase, an Src-type non-receptor tyrosine kinase.	
			SH3GL1	601768	This gene encodes a member of the endophilin family of Src homology 3 domain-containing proteins.	Leukemia, acute myeloid
			CHAF1A	601246	chromatin assembly factor 1, subunit A (p150)	
			UBXN6	611946	UBX domain protein 6	
			PLIN4	613247	perilipin 4	
			PLIN5	613248	perilipin 5	
			LRG1	611289	The leucine-rich repeat (LRR) family of proteins, including LRG1, have been shown to be involved in protein-protein interaction, signal transduction, and cell adhesion and development.	

			SEMA6B	608873	This gene encodes a member of the semaphorin family, a group of proteins characterized by the presence of a conserved semaphorin (sema) domain.	
			C19orf10	606746	The protein encoded by this gene was previously thought to support proliferation of lymphoid cells and was considered an interleukin.	
			DPP9	608258	This gene encodes a protein that is a member of the S9B family in clan SC of the serine proteases.	
			FEM1A	613538	fem-1 homolog a (C. elegans)	
			TICAM1	607601	This gene encodes an adaptor protein containing a Toll/interleukin-1 receptor (TIR) homology domain, which is an intracellular signaling domain that mediates protein-protein interactions between the Toll-like receptors<br>(TLRs) and signal-transduction	

					components.	
			PLIN3	602702	Mannose 6-phophate receptors (MPRs) deliver lysosomal hydrolase from the Golgi to endosomes and then return to the Golgi complex. The protein encoded by this gene interacts with the cytoplasmic domains of both cation-independent and cation-dependent MPRs, and is required for endosome-to-Golgi transport.	
			UHRF1	607990	This gene encodes a member of a subfamily of RING-finger type E3 ubiquitin ligases.	
			KDM4B	609765	lysine (K)-specific demethylase 4B	
**19**	**5q35.3**	**duplication**				
			SLC34A1	182309	This gene encodes a member of the type II sodium-phosphate cotransporter family.	Fanconi renotubular syndrome 2 Nephrolithiasis/osteoporosis, hypophosphatemic, 1

			PFN3	612812	The product of this gene belongs to the profilin family of proteins. This protein binds to actin and affects the structure of the cytoskeleton.	
			F12	610619	This gene encodes coagulation factor XII which circulates in blood as a zymogen.	Angioedema, hereditary, type III<br>Factor XII deficiency
			GRK6	600869	This gene encodes a member of the guanine nucleotide-binding protein (G protein)-coupled receptor kinase subfamily of the Ser/Thr protein kinase family.	
			DBN1	126660	The protein encoded by this gene is a cytoplasmic actin-binding protein thought to play a role in the process of neuronal growth.	
			PDLIM7	605903	The protein encoded by this gene is representative of a family of proteins composed of conserved PDZ and LIM domains.	
			DOK3	611435	docking protein 3	

			DDX41	608170	DEAD box proteins, characterized by the conserved motif Asp-Glu-Ala-Asp (DEAD), are putative RNA helicases. They are implicated in a number of cellular processes involving alteration of RNA secondary structure, such as translation initiation, nuclear and mitochondrial splicing, and ribosome and spliceosome assembly.	
	12q24.11	duplication				
			PPTC7	609668	PTC7 protein phosphatase homolog (S. cerevisiae)	
			TCTN1	609863	This gene encodes a member of the tectonic family of secreted and transmembrane proteins.	Joubert syndrome 13
			HVCN1	611227	This gene encodes a voltage-gated protein channel protein expressed more highly in certain cells of the immune system.	
			PPP1CC	176914	The protein encoded by this gene belongs to the protein phosphatase	

					family, PP1 subfamily. PP1 is an ubiquitous serine/threonine phosphatase that regulates many cellular processes, including cell division.	
	16q12.1	deletion				
			ITFG1	611803	integrin alpha FG-GAP repeat containing 1	
**20**	**10q26.2q26.3**	**deletion**				
			DOCK1	601403	This gene product binds to the SH3 domain of CRK protein.	
			NPS	609513	neuropeptide S	
			PTPRE	600926	The protein encoded by this gene is a member of the protein tyrosine phosphatase (PTP) family. PTPs are known to be signaling molecules that regulate a variety of cellular processes including cell growth, differentiation, mitotic cycle, and oncogenic transformation.	

			MKI67	176741	This gene encodes a nuclear protein that is associated with and may be necessary for cellular proliferation.	
			MGMT	156569	O-6-methylguanine-DNA methyltransferase	
			EBF3	607407	This gene encodes a member of the early B-cell factor (EBF) family of DNA binding transcription factors.	
			GLRX3	612754	This gene encodes a member of the glutaredoxin family.	
			PPP2R2D	613992	protein phosphatase 2, regulatory subunit B, delta	
			BNIP3	603293	This gene is a member of the BCL2/adenovirus E1B 19 kd-interacting protein (BNIP) family. It interacts with the E1B 19 kDa protein, which protects cells from virally-induced cell death.	
			JAKMIP3	611198	Janus kinase and microtubule interacting protein 3	
			DPYSL4	608407	dihydropyrimidinase-like 4	

	17q12	deletion				
			TBC1D3C	610806	The proteins encoded by this gene family contain a TBC (Tre-2, Bub2p, and Cdc16p) domain, which is found in proteins involved in RAB GTPase signaling and vesicle trafficking.	
			CCL3L2	609467	chemokine (C-C motif) ligand 3 pseudogene 1	
			CCL3L1	601 395	This protein binds to several chemokine receptors including chemokine binding protein 2 and chemokine (C-C motif) receptor 5 (CCR5).	{HIV/AIDS, susceptibility to}
**21**	**5q13.2**	**duplication**				
			SMN1	600354	The protein encoded by this gene localizes to both the cytoplasm and the nucleus. Within the nucleus, the protein localizes to subnuclear bodies called gems which are found near coiled bodies containing high concentrations	Spinal muscular atrophy-1<br>Spinal muscular atrophy-2<br>Spinal muscular atrophy-3<br>Spinal muscular atrophy-4

					of small ribonucleoproteins (snRNPs). This protein forms heteromeric complexes with proteins such as SIP1 and GEMIN4, and also interacts with several proteins known to be involved in the biogenesis of snRNPs, such as hnRNP U protein and the small nucleolar RNA binding protein.	
			NAIP	600355	This copy of the gene is full length; additional copies with truncations and internal deletions are also present in this region of chromosome 5q13. It is thought that this gene is a modifier of spinal muscular atrophy caused by mutations in a neighboring gene, SMN1. The protein encoded by this gene contains regions of homology to two baculovirus inhibitor of apoptosis proteins, and it is able to suppress apoptosis induced by various signals.	

			GTF2H2	601748	This gene encodes the 44 kDa subunit of RNA polymerase II transcription initiation factor IIH which is involved in basal transcription and nucleotide excision repair.	
	Yq11.223	duplication				
			DAZ3	400027	This gene is a member of the DAZ gene family and is a candidate for the human Y-chromosomal azoospermia factor (AZF).	
**22**	**1p36.33**	**duplication**				
			GABRD	137163	Gamma-aminobutyric acid (GABA) is the major inhibitory neurotransmitter in the mammalian brain where it acts at GABA-A receptors, which are ligand-gated chloride channels.	{Epilepsy, idiopathic generalized, 10} {Epilepsy, juvenile myoclonic, susceptibility to} {Generalized epilepsy with febrile seizures plus, type 5, susceptibility to}
			PRKCZ	176982	Protein kinase C (PKC) zeta is a member of the PKC family of serine/threonine kinases which are	

					involved in a variety of cellular processes such as proliferation, differentiation and secretion.	
			SKI	164780	This gene encodes the nuclear protooncogene protein homolog of avian sarcoma viral (v-ski) oncogene. It functions as a repressor of TGF-beta signaling, and may play a role in neural tube development and muscle differentiation.	
	7p22.3	duplication				
			GPER	601805	This gene is a member of the G-protein coupled receptor 1 family and encodes a multi-pass membrane protein that localizes to the endoplasmic reticulum.	
			ZFAND2A	610699	zinc finger, AN1-type domain 2A	
			INTS1	611345	INTS1 is a subunit of the Integrator complex, which associates with the C-terminal domain of RNA polymerase II large subunit	

					(POLR2A; MIM 180660) and mediates 3-prime end processing of small nuclear RNAs U1	
			MAFK	600197	The developmentally regulated expression of the globin genes depends on upstream regulatory elements termed locus control regions (LCRs).	
			MAD1L1	602686	MAD1L1 is a component of the mitotic spindle-assembly checkpoint that prevents the onset of anaphase until all chromosome are properly aligned at the metaphase plate.	Lymphoma, somatic<br>Prostate cancer, somatic
	16p13.3	duplication				
			RAB11FIP3	608738	Proteins of the large Rab GTPase family (see RAB1A; MIM 179508) have regulatory roles in the formation, targeting, and fusion of intracellular transport vesicles.	
			SOLH	603267	This gene is involved in the first step in glycosylphosphatidylinositol (GPI)-	

					anchor biosynthesis.	
			PIGQ	605754	This gene encodes a secreted multidomain protein consisting of a signal peptide, a WAP domain, a follistatin domain, an immunoglobulin domain, two tandem Kunitz domains, and an NTR domain.	
			WFIKKN1	608021	This gene encodes a member of the Rho family of GTPases.	
			RHOT2	613889	rhomboid, veinlet-like 1 (Drosophila)	
			RHBDL1	603264	STIP1 homology and U-box containing protein 1, E3 ubiquitin protein ligase	
			STUB1	607207	STUB1, or CHIP, is a ubiquitin ligase/cochaperone that participates in protein quality control by targeting a broad range of chaperone protein substrates for degradation	
			FBXL16	609082	F-box and leucine-rich repeat protein 16	

			METRN	610998	Meteorin regulates glial cell differentiation and promotes the formation of axonal networks during neurogenesis	
			NARFL	611118	nuclear prelamin A recognition factor-like	
			MSLN	601051	This gene encodes a precursor protein that is cleaved into two products, megakaryocyte potentiating factor and mesothelin.	
			CHTF18	613201	CTF18, chromosome transmission fidelity factor 18 homolog (S. cerevisiae)	
			GNG13	607298	GNG13 is a gamma subunit that is expressed in taste, retinal, and neuronal tissues and plays a key role in taste transduction	
			LMF1	611761	The protein encoded by this gene resides in the endoplasmic reticulum, and is involved in the maturation and transport of lipoprotein lipase through	Lipase deficiency, combined

					the secretory pathway.	
			SOX8	605923	This gene encodes a member of the SOX (SRY-related HMG-box) family of transcription factors involved in the regulation of embryonic development and in the determination of the cell fate.	
			SSTR5	182455	Somatostatin and its related peptide cortistatin exert multiple biological actions on normal and tumoral tissue targets by interacting with somatostatin receptors (SSTRs). The protein encoded by this gene is one of the SSTRs, which is a multi-pass membrane protein and belongs to the G-protein coupled receptor 1 family.	Somatostatin analog, resistance to
			C1QTNF8	614147	C1q and tumor necrosis factor related protein 8	
	17p13.3	duplication				
			NXN	612895	nucleoredoxin	

			TIMM22	607251	translocase of inner mitochondrial membrane 22 homolog (yeast)	
			ABR	600365	The protein encoded by this gene contains a GTPase-activating protein domain, a domain found in members of the Rho family of GTP-binding proteins.	
	20q13.33	duplication				
			OSBPL2	606731	This gene encodes a member of the oxysterol-binding protein (OSBP) family, a group of intracellular lipid receptors.	
			ADRM1	610650	The protein encoded by this gene is an integral plasma membrane protein which promotes cell adhesion.	
			LAMA5	601033	The protein encoded by this gene belongs to the alpha subfamily of laminin chains and is a major component of basement membranes.	
			RPS21	180477	This gene encodes a ribosomal protein that is a component of the	

	[redacted]	[redacted]			40S subunit.	
	[redacted]	[redacted]	GATA5	611496	The protein encoded by this gene is a transcription factor that contains two GATA-type zinc fingers.	
	[redacted]	[redacted]	MIR1-1	609326	microRNAs (miRNAs) are short (20-24 nt) non-coding RNAs that are involved in post-transcriptional regulation of gene expression in multicellular organisms by affecting both the stability and translation of mRNAs.	
	[redacted]	[redacted]	MIR133A2	610255	microRNAs (miRNAs) are short (20-24 nt) non-coding RNAs that are involved in post-transcriptional regulation of gene expression in multicellular organisms by affecting both the stability and translation of mRNAs.	
	[redacted]	[redacted]	SLCO4A1	612436	solute carrier organic anion transporter family, member 4A1	

			NTSR1	162651	Neurotensin receptor 1 belongs to the large superfamily of G-protein coupled receptors.<br>NTSR1 mediates the multiple functions of neurotensin, such as hypotension, hyperglycemia, hypothermia, antinociception, and regulation of intestinal motility and secretion.	
			C20orf20	611157	chromosome 20 open reading frame 20	
			OGFR	606459	The protein encoded by this gene is a receptor for opioid growth factor (OGF), also known as [Met(5)]-enkephalin.<br>OGF is a negative regulator of cell proliferation and tissue organization in a variety of processes. The encoded unbound receptor for OGF has been localized to the outer nuclear envelope, where it binds OGF	

	[redacted]	[redacted]			and is translocated into the nucleus.	
	[redacted]	[redacted]	COL9A3	120270	This gene encodes one of the three alpha chains of type IX collagen, the major collagen component of hyaline cartilage.	Epiphyseal dysplasia, multiple, 3<br>Epiphyseal dysplasia, multiple, with myopathy<br>{Intervertebral disc disease, susceptibility to}
	[redacted]	[redacted]	TCFL5	604745	transcription factor-like 5 (basic helix-loop-helix)	
	[redacted]	[redacted]	DIDO1	604140	This gene is similar to the mouse gene and therefore is thought to be involved in apoptosis.	
	[redacted]	[redacted]	C20orf11	611625	chromosome 20 open reading frame 11	
	[redacted]	[redacted]	SLC17A9	612107	solute carrier family 17, member 9	
	[redacted]	[redacted]	BHLHE23	609331	basic helix-loop-helix family, member e23	
	[redacted]	[redacted]	HAR1B	610557	highly accelerated region 1B (non-protein coding)	
	[redacted]	[redacted]	HAR1A	610556	highly accelerated region 1A (non-	

	[redacted]	[redacted]			protein coding)	
	[redacted]	[redacted]	BIRC7	605737	The protein encoded by this gene is a member of the family of inhibitor of apoptosis proteins (IAP) and contains a single copy of a baculovirus IAP repeat (BIR) as well as a RING-type zinc finger domain.	
	[redacted]	[redacted]	NKAIN4	612873	Na+/K+ transporting ATPase interacting 4	
	[redacted]	[redacted]	ARFGAP1	608377	The protein encoded by this gene is a GTPase-activating protein (GAP) which associates with the Golgi apparatus and which interacts with ADP-ribosylation factor 1 (ARF1).	
	[redacted]	[redacted]	CHRNA4	118504	This gene encodes a nicotinic acetylcholine receptor, which belongs to a superfamily of ligand-gated ion channels that play a role in fast signal transmission at synapses.	Epilepsy, nocturnal frontal lobe, 1 {Nicotine addiction, susceptibility to}
	[redacted]	[redacted]	KCNQ2	602235	The M channel is a slowly activating and deactivating potassium channel	Epileptic encephalopathy, early infantile, 7

	[redacted]	[redacted]			that plays a critical role in the regulation of neuronal excitability.	Myokymia<br>Seizures, benign neonatal, 1
	[redacted]	[redacted]	EEF1A2	602959	This gene encodes an isoform of the alpha subunit of the elongation factor-1 complex, which is responsible for the enzymatic delivery of aminoacyl tRNAs to the ribosome	
	[redacted]	[redacted]	PTK6	602004	The protein encoded by this gene is a cytoplasmic nonreceptor protein kinase which may function as an intracellular signal transducer in epithelial tissues.	
	[redacted]	[redacted]	PRIC285	611265	The protein encoded by this gene is a nuclear transcriptional co-activator for peroxisome proliferator activated receptor alpha.	
	[redacted]	[redacted]	GMEB2	607451	glucocorticoid modulatory element binding protein 2	
	[redacted]	[redacted]	STMN3	608362	The protein encoded by this gene belongs to the stathmin/oncoprotein 18 family of microtubule-destabilizing	

					phosphoproteins.	
			RTEL1	608833	regulator of telomere elongation helicase 1	
			TNFRSF6B	603361	This gene belongs to the tumor necrosis factor receptor superfamily. The encoded protein is postulated to play a regulatory role in suppressing FasL- and LIGHT-mediated cell death. It acts as a decoy receptor that competes with death receptors for ligand binding.	
			ARFRP1	604699	The protein encoded by this gene is a membrane-associated GTP-ase and localizes to the plasma membrane.	
			LIME1	609809	Lck interacting transmembrane adaptor 1	
			SLC2A4RG	609493	The protein encoded by this gene is a nuclear transcription factor involved in the activation of the solute carrier family 2 member 4 gene.	
			TPD52L2	603747	This gene encodes a member of the	

					tumor protein D52-like family.	
			DNAJC5	611203	This gene is a member of the J protein family. J proteins function in many cellular processes by regulating the ATPase activity of 70 kDa heat shock proteins. The encoded protein plays a role in membrane trafficking and protein folding, and has been shown to have anti-neurodegenerative properties.	Ceroid lipofuscinosis, neuronal, 4, Parry type
			UCKL1	610866	uridine-cytidine kinase 1-like 1	
			PRPF6	613979	The protein encoded by this gene appears to be involved in pre-mRNA splicing, possibly acting as a bridging factor between U5 and U4/U6 snRNPs in formation of the spliceosome.	Retinitis pigmentosa 60
	22q13.33	duplication				
			BRD1	604589	This gene encodes a protein of unknown function.	

			ZBED4	612552	zinc finger, BED-type containing 4	
			ALG12	607144	This gene encodes a member of the glycosyltransferase 22 family.	Congenital disorder of glycosylation, type Ig
			CRELD2	607171	cysteine-rich with EGF-like domains 2	
			PIM3	610580	PIM3 belongs to a family of protooncogenes that encode serine/threonine protein kinases	
			IL17REL	613414	interleukin 17 receptor E-like	
			MLC1	605908	The function of this gene product is unknown	Megalencephalic leukoencephalopathy with subcortical cysts
**23**	**14q32.2**	**duplication**				
			DLK1	176290	This gene encodes a transmembrane protein containing six epidermal growth factor repeats.	
			MEG3	605636	This gene is a maternally expressed imprinted gene.	
	16p13.3	duplication				
			MSLN	601051	This gene encodes a precursor protein that is cleaved into two	

					products, megakaryocyte potentiating factor and mesothelin.	
			CHTF18	613201	CTF18, chromosome transmission fidelity factor 18 homolog (S. cerevisiae)	
			GNG13	607298	Heterotrimeric G proteins, which consist of alpha (see MIM 139320), beta (see MIM 139380), and gamma subunits, function as signal transducers for the 7-transmembrane-helix G protein-coupled receptors.	
			LMF1	611761	The protein encoded by this gene resides in the endoplasmic reticulum, and is involved in the maturation and transport of lipoprotein lipase through the secretory pathway.	Lipase deficiency, combined
	22q13.1	duplication				
			GGA1	606004	This gene encodes a member of the Golgi-localized, gamma adaptin ear-containing, ARF-binding (GGA)	

					protein family.	
	Xq28	duplication				
			L1CAM	308840	The protein encoded by this gene is an axonal glycoprotein belonging to the immunoglobulin supergene family.	Corpus callosum, partial agenesis of<br>CRASH syndrome<br>Hydrocephalus due to aqueductal stenosis<br>Hydrocephalus with congenital idiopathic intestinal pseudoobstruction<br>Hydrocephalus with Hirschsprung disease<br>MASA syndrome
			AVPR2	300538	This gene encodes the vasopressin receptor, type 2, also known as the V2 receptor, which belongs to the seven-transmembrane-domain G protein-coupled receptor (GPCR) superfamily, and couples to Gs thus stimulating adenylate cyclase.	Diabetes insipidus, nephrogenic<br>Nephrogenic syndrome of inappropriate antidiuresis
			ARHGAP4	300023	This gene encodes a member of the	

					rhoGAP family of proteins which play a role in the regulation of small GTP-binding proteins belonging to the RAS superfamily.	
			NAA10	300013	N-alpha-acetylation is among the most common post-translational protein modifications in eukaryotic cells.	N-terminal acetyltransferase deficiency
			RENBP	312420	The gene product inhibits renin activity by forming a dimer with renin, a complex known as high molecular weight renin.	
			HCFC1	300019	This gene is a member of the host cell factor family and encodes a protein with five Kelch repeats, a fibronectin-like motif, and six HCF repeats, each of which contains a highly specific cleavage signal.	
			TMEM187	300059	This gene consists of two exons and encodes a multi-pass membrane protein.	

			IRAK1	300283	This gene encodes the interleukin-1 receptor-associated kinase 1, one of two putative serine/threonine kinases that become associated with the interleukin-1 receptor (IL1R) upon stimulation.	
			MECP2	300 005	DNA methylation is the major modification of eukaryotic genomes and plays an essential role in mammalian development.<br>Human proteins MECP2, MBD1, MBD2, MBD3, and MBD4 comprise a family of nuclear proteins related by the presence in each of a methyl-CpG binding domain (MBD).	Angelman syndrome<br>Encephalopathy, neonatal severe<br>Mental retardation, X-linked syndromic, Lubs type<br>Mental retardation, X-linked, syndromic 13<br>Rett syndrome<br>Rett syndrome, preserved speech variant<br>{Autism susceptibility, X-linked 3}
			OPN1LW	300822	This gene encodes for a light absorbing visual pigment of the opsin gene family.	Blue cone monochromacy<br>Colorblindness, protan

			OPN1MW	300821	This gene encodes for a light absorbing visual pigment of the opsin gene family. The encoded protein is called green cone photopigment or medium-wavelength sensitive opsin.	Blue cone monochromacy<br>Colorblindness, protan
			TEX28	300092	testis expressed 28	
			TKTL1	300044	The protein encoded by this gene is a transketolase that acts as a homodimer and catalyzes the conversion of sedoheptulose 7-phosphate and D-glyceraldehyde 3-phosphate to D-ribose 5-phosphate and D-xylulose 5-phosphate.	
			FLNA	300017	The protein encoded by this gene is an actin-binding protein that crosslinks actin filaments and links actin filaments to membrane glycoproteins.	Cardiac valvular dysplasia, X-linked<br>FG syndrome 2<br>Frontometaphyseal dysplasia<br>Heterotopia, periventricular<br>Heterotopia, periventricular, ED variant

						Intestinal pseudoobstruction, neuronal<br>Melnick-Needles syndrome<br>Otopalatodigital syndrome, type I<br>Otopalatodigital syndrome, type II<br>Terminal osseous dysplasia
			EMD	300384	Emerin is a serine-rich nuclear membrane protein and a member of the nuclear lamina-associated protein family. It mediates membrane anchorage to the cytoskeleton.	Emery-Dreifuss muscular dystrophy 1, X-linked
			RPL10	312173	This gene encodes a ribosomal protein that is a component of the 60S subunit.	{Autism, susceptibility to, X-linked 5}
			DNASE1L1	300081	This gene encodes a member of the deoxyribonuclease family and the protein shows high sequence similarity to lysosomal DNase I.	

			TAZ	300394	This gene encodes a protein that is expressed at high levels in cardiac and skeletal muscle.	Barth syndrome<br>Cardiomyopathy, dilated, 3A<br>Left ventricular noncompaction, X-linked
**24**	**16p13.3**	**duplication**				
			SLX4	613278	This gene encodes a structure-specific endonuclease subunit.	Fanconi anemia, complementation group P
			DNASE1	125505	This gene encodes a member of the DNase family.	{Systemic lupus erythematosus, susceptibility to}
			TRAP1	606219	HSP90 proteins are highly conserved molecular chaperones that have key roles in signal transduction, protein folding, protein degradation, and morphologic evolution.	
			CREBBP	600140	This gene is ubiquitously expressed and is involved in the transcriptional coactivation of many different transcription factors.	Rubinstein-Taybi syndrome

**26**	**9q34.2q34.3**	**deletion**				
			GTF3C4	604892	general transcription factor IIIC, polypeptide 4, 90kDa	
			TSC1	605284	This gene encodes a growth inhibitory protein thought to play a role in the stabilization of tuberin.	Focal cortical dysplasia, Taylor balloon cell type Lymphangioleiomyomatosis Tuberous sclerosis-1
			GFI1B	604383	growth factor independent 1B transcription repressor	
			GTF3C5	604890	general transcription factor IIIC, polypeptide 5, 63kDa	
			CEL	114840	The protein encoded by this gene is a glycoprotein secreted from the pancreas into the digestive tract and from the lactating mammary gland into human milk.	Maturity-onset diabetes of the young, type VIII
			RALGDS	601619	ral guanine nucleotide dissociation stimulator	
			GBGT1	606074	This gene encodes a member of the histo-blood group ABO gene family that encodes glycosyltransferases	

					with related but distinct substrate specificity.	
			OBP2B	604606	odorant binding protein 2B	
			ABO	110300	This gene encodes proteins related to the first discovered blood group system, ABO.	[Blood group, ABO system]
			SURF6	185642	This gene is located in the surfeit gene cluster, a group of very tightly linked genes that do not share sequence similarity.	
			MED22	185641	This gene is located in the surfeit gene cluster, a group of very tightly linked housekeeping genes that do not share sequence similarity.	
			RPL7A	185640	ribosomal protein L7a	
			SURF1	185620	This gene encodes a protein localized to the inner mitochondrial membrane and thought to be involved in the biogenesis of the cytochrome c oxidase complex.	Leigh syndrome, due to COX deficiency
			SURF2	185630	This gene is located in the surfeit	

					gene cluster, a group of very tightly linked genes that do not share sequence similarity.	
			SURF4	185660	This gene is located in the surfeit gene cluster, which is comprised of very tightly linked housekeeping genes that do not share sequence similarity. The encoded protein is a conserved integral membrane protein containing multiple putative transmembrane regions.	
			REXO4	602930	REX4, RNA exonuclease 4 homolog (S. cerevisiae)	
			ADAMTS13	604134	This gene encodes a member of the ADAMTS (a disintegrin and metalloproteinase with thrombospondin motif) protein family.	Thrombotic thrombocytopenic purpura, familial
			C9orf7	613104	calcium channel flower domain containing 1	
			SLC2A6	606813	solute carrier family 2 (facilitated glucose transporter), member 6	

			ADAMTSL2	612277	This gene encodes a member of the ADAMTS (a disintegrin and metalloproteinase with thrombospondin motifs) and ADAMTS-like protein family.	Geleophysic dysplasia 1
			DBH	609312	The protein encoded by this gene is an oxidoreductase belonging to the copper type II, ascorbate-dependent monooxygenase family.	Dopamine beta-hydroxylase deficiency [Dopamine-beta-hydroxylase activity levels, plasma]
			SARDH	604455	This gene encodes an enzyme localized to the mitochondrial matrix which catalyzes the oxidative demethylation of sarcosine.	Sarcosinemia]
			VAV2	600428	VAV2 is the second member of the VAV guanine nucleotide exchange factor family of oncogenes.	
			BRD3	601541	The function of the encoded protein is not known.	
			WDR5	609012	This gene encodes a member of the WD repeat protein family. WD	

					repeats are minimally conserved regions of approximately 40 amino acids typically bracketed by gly-his and trp-asp (GH-WD), which may facilitate formation of heterotrimeric or multiprotein complexes.	
			RNU6ATAC	601429	RNA, U6atac small nuclear (U12-dependent splicing)	
			RXRA	180245	Retinoid X receptors (RXRs) and retinoic acid receptors (RARs), are nuclear receptors that mediate the biological effects of retinoids by their involvement in retinoic acid-mediated gene activation.	
			COL5A1	120215	This gene encodes an alpha chain for one of the low abundance fibrillar collagens.	Ehlers-Danlos syndrome, type I<br>Ehlers-Danlos syndrome, type II
			FCN2	601624	The product of this gene belongs to the ficolin family of proteins.	
			FCN1	601252	The ficolin family of proteins are	

					characterized by the presence of a leader peptide, a short N-terminal segment, followed by a collagen-like region, and a C-terminal fibrinogen-like domain.	
			OLFM1	605366	the exact function of the encoded protein is not known	
	14q13.1	deletion				
			NPAS3	609430	This gene encodes a member of the basic helix-loop-helix and PAS domain-containing family of transcription factors. The encoded protein is localized to the nucleus and may regulate genes involved in neurogenesis.	
	15q25.2	deletion				
			ARNT2	606036	This gene encodes a member of the basic-helix-loop-helix-Per-Arnt-Sim (bHLH-PAS) superfamily of transcription factors.	
			KIAA1199	608366	KIAA1199	?Deafness, nonsyndromic

	17p11.2	deletion				
			TOP3A	601243	This gene encodes a DNA topoisomerase, an enzyme that controls and alters the topologic states of DNA during transcription.	
			SHMT1	182144	This gene encodes the cellular form of serine hydroxymethyltransferase, a pyridoxal phosphate-containing enzyme<br>that catalyzes the reversible conversion of serine and tetrahydrofolate to glycine and 5,10-methylene tetrahydrofolate.	
	21q22.3	deletion				
			PDXK	179020	The protein encoded by this gene phosphorylates vitamin B6, a step required for the conversion of vitamin B6 to pyridoxal-5-phosphate, an important cofactor in intermediary metabolism.	
			CSTB	601145	The cystatin superfamily	Epilepsy, progressive

					encompasses proteins that contain multiple cystatin-like sequences.	myoclonic 1A (Unverricht and Lundborg)
			RRP1	610 653	The protein encoded by this gene is the putative homolog of the yeast ribosomal RNA processing protein RRP1.	
**27**	**7q36.2q36.3**	**deletion**				
			DPP6	126141	This gene encodes a single-pass type II membrane protein that is a member of the S9B family in clan SC of the serine proteases.	Ventricular fibrillation, paroxysmal familial, 2
			PAXIP1	608254	This protein plays a critical role in maintaining genome stability, condensation of chromatin and progression through mitosis.	{Alzheimer disease, susceptibility to}
			HTR5A	601 305	The neurotransmitter serotonin (5-hydroxytryptamine, 5-HT) has been implicated in a wide range of psychiatric conditions and also has vasoconstrictive and vasodilatory effects.	

			INSIG1	602055	This gene is an insulin-induced gene. It encodes an endoplasmic reticulum (ER) membrane protein that plays a critical role in regulating cholesterol concentrations in cells.	
			EN2	131310	engrailed homeobox 2	{Autism susceptibility 10}
			CNPY1	612493	canopy 1 homolog (zebrafish)	
			SHH	600725	This gene encodes a protein that is instrumental in patterning the early embryo.	Holoprosencephaly-3<br>Microphthalmia with coloboma 5<br>Schizencephaly<br>Single median maxillary central incisor
			PTPRN2	601698	The protein encoded by this gene is a member of the protein tyrosine phosphatase (PTP) family.	
**28**	**7p11.2**	**duplication**				
			LANCL2	612919	LanC lantibiotic synthetase component C-like 2 (bacterial)	
			VOPP1	611915	vesicular, overexpressed in cancer, prosurvival protein 1	

	9q32	duplication				
			ZFP37	602951	zinc finger protein 37 homolog (mouse)	
	11p15.5	duplication				
			ASCL2	601886	This gene is a member of the basic helix-loop-helix (BHLH) family of transcription factors.	
			C11orf21	611033	chromosome 11 open reading frame 21	
			TSPAN32	603853	This gene may play a role in malignancies and diseases that involve this region, and it is also involved in hematopoietic cell function.	
			CD81	186845	CD81 molecule	Immunodeficiency, common variable, 6
			TSSC4	603852	This gene is one of several tumor-suppressing subtransferable fragments located in the imprinted gene domain of 11p15.5, an important tumor-suppressor gene	

					region. A	
			TRPM5	604600	This gene encodes a member of the transient receptor potential (TRP) protein family, which is a diverse group of proteins with structural features typical of ion channels.	
	17q21.31	duplication				
			DHX8	600396	DEAD box proteins, characterized by the conserved motif Asp-Glu-Ala-Asp (DEAD), are putative RNA helicases.	
			ETV4	600711	ets variant 4	
			MEOX1	600147	This gene encodes a member of a subfamily of non-clustered, diverged, antennapedia-like homeobox-containing genes.	
**30**	**3q22.3**	**deletion**				
			NCK1	600508	The protein encoded by this gene is one of the signaling and transforming proteins containing Src homology 2 and 3 (SH2 and SH3) domains.	

	4p16.3	deletion				
			CPLX1	605032	Proteins encoded by the complexin/synaphin gene family are cytosolic proteins that function in synaptic vesicle exocytosis.	
			GAK	602052	In all eukaryotes, the cell cycle is governed by cyclin-dependent protein kinases (CDKs), whose activities are regulated by cyclins and CDK inhibitors in a diverse array of mechanisms that involve the control of phosphorylation and dephosphorylation of Ser, Thr or Tyr residues.	
	Yq11.223	deletion				
			TTTY17A	400040	testis-specific transcript, Y-linked 17A (non-protein coding)	
			TTTY4	400037	testis-specific transcript, Y-linked 4 (non-protein coding)	
			BPY2	400013	he encoded protein interacts with ubiquitin protein ligase E3A and may	

					be involved in male germ cell development and male infertility.	
			DAZ1	400003	deleted in azoospermia 1	?Sertoli-cell-only syndrome
			DAZ2	400026	deleted in azoospermia 2	
			GOLGA2LY1	400035	golgin A2 pseudogene 2, Y-linked	
			DAZ3	400027	deleted in azoospermia 3	
**31**	**1p36.32**	**deletion**				
			PLCH2	612836	phospholipase C, eta 2	
			PANK4	606162	This gene encodes a protein belonging to the pantothenate kinase family.	
			HES5	607348	This gene encodes a member of a family of basic helix-loop-helix transcriptional repressors.	
			TNFRSF14	602746	tumor necrosis factor receptor superfamily, member 14	
	4p16.3	deletion				
			CPLX1	605032	Proteins encoded by the complexin/synaphin gene family are cytosolic proteins that function in	

					synaptic vesicle exocytosis.	
			GAK	602052	Cyclins are molecules that possess a consensus domain called the 'cyclin box.'<br>In mammalian cells, 9 cyclin species have been identified, and they are referred to as cyclins A through I. Cyclin G is a direct transcriptional target of the p53 tumor suppressor gene product and thus functions downstream of p53. GAK is an association partner of cyclin G and CDK5.	
	20q13.33	deletion				
			CDH4	603006	This gene is a classical cadherin from the cadherin superfamily. The encoded protein is a calcium-dependent cell-cell adhesion glycoprotein comprised of five extracellular cadherin repeats, a transmembrane region and a highly	

					conserved cytoplasmic tail.	
**32**	**14q12**	**deletion**				
			CEBPE	600749	The protein encoded by this gene is a bZIP transcription factor which can bind as a homodimer to certain DNA regulatory regions.	Specific granule deficiency
			SLC7A8	604235	solute carrier family 7 (amino acid transporter light chain, L system), member 8	
	Xq13.1	deletion				
			ZMYM3	300061	This gene is located on the X chromosome and is subject to X inactivation. It is highly conserved in vertebrates and most abundantly expressed in the brain.	
			NONO	300084	This gene encodes an RNA-binding protein which plays various roles in the nucleus, including transcriptional regulation and RNA splicing.	
			ITGB1BP2	300332	integrin beta 1 binding protein (melusin) 2	

			TAF1	313650	This gene encodes the largest subunit of TFIID.	Dystonia-Parkinsonism, X-linked
	Xq22.1	deletion				
			CSTF2	600368	This gene encodes a nuclear protein with an RRM (RNA recognition motif) domain.	
			NOX1	300225	Voltage-gated proton (hydrogen) channels play an important role in cellular defense against acidic stress.	
	Xq28	deletion				
			AVPR2	300538	This gene encodes the vasopressin receptor, type 2, also known as the V2 receptor, which belongs to the seven-transmembrane-domain G protein-coupled receptor (GPCR) superfamily, and couples to Gs thus stimulating adenylate cyclase.	Diabetes insipidus, nephrogenic Nephrogenic syndrome of inappropriate antidiuresis
			ARHGAP4	300023	This gene encodes a member of the rhoGAP family of proteins which play a role in the regulation of small GTP-binding proteins belonging to the RAS	

					superfamily.	
			NAA10	300013	N-alpha-acetylation is among the most common post-translational protein modifications in eukaryotic cells.	N-terminal acetyltransferase deficiency
			RENBP	312420	The gene product inhibits renin activity by forming a dimer with renin, a complex known as high molecular weight renin.	
			HCFC1	300019	This gene is a member of the host cell factor family and encodes a protein with five Kelch repeats, a fibronectin-like motif, and six HCF repeats, each of which contains a highly specific cleavage signal.	
			TMEM187	300059	This gene consists of two exons and encodes a multi-pass membrane protein.	
			IRAK1	300283	This gene encodes the interleukin-1 receptor-associated kinase 1, one of two putative serine/threonine kinases	

					that become associated with the interleukin-1 receptor (IL1R) upon stimulation.	
			MECP2	300 005	DNA methylation is the major modification of eukaryotic genomes and plays an essential role in mammalian development. Human proteins MECP2, MBD1, MBD2, MBD3, and MBD4 comprise a family of nuclear proteins related by the presence in each of a methyl-CpG binding domain (MBD).	Angelman syndrome<br>Encephalopathy, neonatal severe<br>Mental retardation, X-linked syndromic, Lubs type<br>Mental retardation, X-linked, syndromic 13<br>Rett syndrome<br>Rett syndrome, preserved speech variant<br>{Autism susceptibility, X-linked 3}
**34**	**9q34.3**	**duplication**				
			COL5A1	120215	This gene encodes an alpha chain for one of the low abundance fibrillar collagens.	Ehlers-Danlos syndrome, type I<br>Ehlers-Danlos syndrome, type II
			FCN2	601624	The product of this gene belongs to	

					the ficolin family of proteins. This family is characterized by the presence of a leader peptide, a short N-terminal segment, followed by a collagen-like region, and a C-terminal fibrinogen-like domain.	
			FCN1	601252	The ficolin family of proteins are characterized by the presence of a leader peptide, a short N-terminal segment, followed by a collagen-like region, and a C-terminal fibrinogen-like domain.	
**35**	**Xq25**	**deletion**				
			BCORL1	300688	The protein encoded by this gene is a transcriptional corepressor that is found tethered to promoter regions by DNA-binding proteins.	
			ELF4	300775	The protein encoded by this gene is a transcriptional activator that binds and activates the promoters of the CSF2, IL3, IL8, and PRF1 genes.	

			AIFM1	300169	This gene encodes a flavoprotein essential for nuclear disassembly in apoptotic cells, and it is found in the mitochondrial intermembrane space in healthy cells.	Combined oxidative phosphorylation deficiency 6
	Xq28	deletion				
			AVPR2	300538	This gene encodes the vasopressin receptor, type 2, also known as the V2 receptor, which belongs to the seven-transmembrane-domain G protein-coupled receptor (GPCR) superfamily, and couples to Gs thus stimulating adenylate cyclase.	Diabetes insipidus, nephrogenic<br>Nephrogenic syndrome of inappropriate antidiuresis
			ARHGAP4	300023	This gene encodes a member of the rhoGAP family of proteins which play a role in the regulation of small GTP-binding proteins belonging to the RAS superfamily.	
			NAA10	300013	N-alpha-acetylation is among the most common post-translational protein modifications in eukaryotic	N-terminal acetyltransferase deficiency

					cells.	
			RENBP	312420	The gene product inhibits renin activity by forming a dimer with renin, a complex known as high molecular weight renin.	
			HCFC1	300019	This gene is a member of the host cell factor family and encodes a protein with five Kelch repeats, a fibronectin-like motif, and six HCF repeats, each of which contains a highly specific cleavage signal.	
			TMEM187	300059	This gene consists of two exons and encodes a multi-pass membrane protein.	
			IRAK1	300283	This gene encodes the interleukin-1 receptor-associated kinase 1, one of two putative serine/threonine kinases that become associated with the interleukin-1 receptor (IL1R) upon stimulation.	

			MECP2	300 005	DNA methylation is the major modification of eukaryotic genomes and plays an essential role in mammalian development. Human proteins MECP2, MBD1, MBD2, MBD3, and MBD4 comprise a family of nuclear proteins related by the presence in each of a methyl-CpG binding domain (MBD).	Angelman syndrome<br>Encephalopathy, neonatal severe<br>Mental retardation, X-linked syndromic, Lubs type<br>Mental retardation, X-linked, syndromic 13<br>Rett syndrome<br>Rett syndrome, preserved speech variant<br>{Autism susceptibility, X-linked 3}
**38**	**Xq12**	**deletion**				
			AR	313700	The androgen receptor gene is more than 90 kb long and codes for a protein that has 3 major functional domains: the N-terminal domain, DNA-binding domain, and androgen-binding domain. The protein functions as a steroid-hormone activated transcription factor.	Androgen insensitivity<br>Androgen insensitivity, partial, with or without breast cancer<br>Hypospadias 1, X-linked<br>Spinal and bulbar muscular atrophy of Kennedy<br>{Prostate cancer,

						susceptibility to}
**39**	**13q14.3**	**deletion**				
			RB1	614041	The protein encoded by this gene is a negative regulator of the cell cycle and was the first tumor suppressor gene found.	Bladder cancer, somatic<br>Osteosarcoma, somatic<br>Retinoblastoma<br>Retinoblastoma, trilateral<br>Small cell cancer of the lung, somatic
			LPAR6	609239	The protein encoded by this gene belongs to the family of G-protein coupled receptors, that are preferentially activated by adenosine and uridine nucleotides.	Hypotrichosis 8<br>Woolly hair, autosomal recessive 1, with or without hypotrichosis
			RCBTB2	603524	This gene encodes a member of the RCC1-related GEF family.	
	Xp22.2	deletion				
			MID1	300552	The protein encoded by this gene is a member of the tripartite motif (TRIM) family, also known as the 'RING-B	Opitz GBBB syndrome, type I

					box-coiled coil' (RBCC) subgroup of RING finger proteins.	
**40**	**5q13.2**	**duplication**				
			SMN2	601627	This protein forms heteromeric complexes with proteins such as SIP1 and GEMIN4, and also interacts with several proteins known to be involved in the biogenesis of snRNPs, such as hnRNP U protein and the small nucleolar RNA binding protein.	{Spinal muscular atrophy, type III, modifier of}
			SERF1A	603011	The function of this protein is not known	
			NAIP	600355	The protein encoded by this gene contains regions of homology to two baculovirus inhibitor of apoptosis proteins, and it is able to suppress apoptosis induced by various signals.	
			GTF2H2	601748	pro-melanin-concentrating hormone-like 2, pseudogene	
			PMCHL2	176794	. The encoded protein localizes to concentrated aggregates in the	

					nucleus, and is required for transcription from all three types of polymerase III promoters.	
			BDP1	607012	The product of this gene is a subunit of the TFIIIB transcription initiation complex, which recruits RNA polymerase III to target promoters in order to initiate transcription.	
			MCCC2	609014	This gene encodes the small subunit of 3-methylcrotonyl-CoA carboxylase.	3-Methylcrotonyl-CoA carboxylase 2 deficiency
**41**	**12q24.31**	**deletion**				
			EIF2B1	606686	This gene encodes one of five subunits of eukaryotic translation initiation factor 2B (EIF2B), a GTP exchange factor for eukaryotic initiation factor 2 and an essential regulator for protein synthesis.	Leukoencephalopathy with vanishing white matter
			GTF2H3	601750	general transcription factor IIH, polypeptide 3, 34kDa	
			TCTN2	613846	This gene encodes a type I membrane protein that belongs to the	Meckel syndrome, type 8

					tectonic family.	
			ATP6V0A2	611716	The protein encoded by this gene is a subunit of the vacuolar ATPase (v-ATPase), an heteromultimeric enzyme that is present in intracellular vesicles and in the plasma membrane of specialized cells, and which is essential for the acidification of diverse cellular components.	Cutis laxa, autosomal recessive, type IIA Wrinkly skin syndrome
	20q13.13	deletion				
			KCNB1	600397	Voltage-gated potassium (Kv) channels represent the most complex class of voltage-gated ion channels from both functional and structural standpoints.	
			PTGIS	601699	This gene encodes a member of the cytochrome P450 superfamily of enzymes. The cytochrome P450 proteins are monooxygenases which catalyze many reactions involved in drug metabolism and synthesis of	Hypertension, essential

					cholesterol, steroids and other lipids.	
	Xq21.1	deletion				
			DMD	300377	Dystrophin is part of the dystrophin-glycoprotein complex (DGC), which bridges the inner cytoskeleton (F-actin) and the extra-cellular matrix.	Becker muscular dystrophy Cardiomyopathy, dilated, 3B Duchenne muscular dystrophy
	Xq13.3	deletion				
			ZDHHC15	300576	The protein encoded by this gene belongs to the DHHC palmitoyltransferase family.	Mental retardation, X-linked 91
**42**	**1p36.32**	**deletion**				
			PLCH2	612836	PLCH2 is a member of the PLC-eta family of the phosphoinositide-specific phospholipase C (PLC) superfamily of enzymes that cleave PtdIns(4,5) P2 to generate second messengers inositol 1,4,5-trisphosphate and diacylglycerol	
			PANK4	606162	This gene encodes a protein belonging to the pantothenate kinase family. Pantothenate kinase is a key	

					regulatory enzyme in the biosynthesis of coenzyme A (CoA) in bacteria and mammalian cells.	
			HES5	607348	This gene encodes a member of a family of basic helix-loop-helix transcriptional repressors. The protein product of this gene, which is activated downstream of the Notch pathway, regulates cell differentiation in multiple tissues.	
			TNFRSF14	602746	The protein encoded by this gene is a member of the TNF-receptor superfamily. This receptor was identified as a cellular mediator of herpes simplex virus (HSV) entry.	
			ACTRT2	608535	The protein encoded by this intronless gene belongs to the actin family.	
			PRDM16	605557	The protein encoded by this gene is a zinc finger transcription factor and contains an N-terminal PR domain.	

	5p15.33	deletion				
			AHRR	606517	The protein encoded by this gene participates in the aryl hydrocarbon receptor (AhR) signaling cascade, which mediates dioxin toxicity, and is involved in regulation of cell growth and differentiation.	
			EXOC3	608186	The protein encoded by this gene is a component of the exocyst complex, a multiple protein complex essential for targeting exocytic vesicles to specific docking sites on the plasma membrane.	
			SLC9A3	182307	solute carrier family 9, subfamily A (NHE3, cation proton antiporter 3), member 3	
			TPPP	608773	tubulin polymerization promoting protein	
			TRIP13	604507	This gene encodes a protein that interacts with thyroid hormone receptors, also known as hormone-	

					dependent transcription factors.	
			NKD2	607852	naked cuticle homolog 2 (Drosophila)	
			SLC12A7	604879	solute carrier family 12 (potassium/chloride transporters), member 7	
			SLC6A19	608893	This gene encodes a system B(0) transmembrane protein that actively transports most neutral amino acids across the apical membrane of epithelial cells.	Hartnup disorder<br>Hyperglycinuria<br>Iminoglycinuria, digenic
			SLC6A18	610300	The SLC6 family of proteins, which includes SLC6A18, act as specific transporters for neurotransmitters, amino acids, and osmolytes like betaine,<br>taurine, and creatine. SLC6 proteins are sodium cotransporters that derive the energy for solute transport from the electrochemical gradient for sodium ions	

			TERT	187270	Telomerase is a ribonucleoprotein polymerase that maintains telomere ends by addition of the telomere repeat TTAGGG.	Dyskeratosis congenita, autosomal dominant 2 Dyskeratosis congenita, autosomal recessive 4 {Aplastic anemia, susceptibility to} {Coronary artery disease, susceptiblity to} {Pulmonary fibrosis,idiopathic, susceptibility to}
			CLPTM1L	612585	CLPTM1-like	
			SLC6A3	126455	This gene encodes a dopamine transporter which is a member of the sodium- and chloride-dependent neurotransmitter transporter family.	Parkinsonism-dystonia, infantile {Attention-deficit hyperactivity disorder, susceptibility to} {Major affective disorder} {Nicotine dependence, protection against}
			LPCAT1	610472	lysophosphatidylcholine	

					acyltransferase 1	
**43**	**9q34.2**	**deletion**				
			GTF3C4	604892	general transcription factor IIIC, polypeptide 4, 90kDa	
			TSC1	605284	This gene encodes a growth inhibitory protein thought to play a role in the stabilization of tuberin.	Focal cortical dysplasia, Taylor balloon cell type<br>Lymphangioleiomyomatosis<br>Tuberous sclerosis-1
			GFI1B	604383	growth factor independent 1B transcription repressor	
			GTF3C5	604890	general transcription factor IIIC, polypeptide 5, 63kDa	
			CEL	114840	The protein encoded by this gene is a glycoprotein secreted from the pancreas into the digestive tract and from the lactating mammary gland into human milk.	Maturity-onset diabetes of the young, type VIII
			RALGDS	601619	Guanine nucleotide dissociation stimulators (GDSs, or exchange factors), such as RALGDS, are effectors of Ras-related GTPases	

					(see MIM 190020) that participate in signaling for a variety of cellular processes.	
			GBGT1	606074	This gene encodes a member of the histo-blood group ABO gene family that encodes glycosyltransferases with related but distinct substrate specificity.	
			OBP2B	604606	odorant binding protein 2B	
			ABO	110300	This gene encodes proteins related to the first discovered blood group system, ABO. Which allele is present in an individual determines the blood group. T	[Blood group, ABO system]
			SURF6	185642	The gene demonstrates features of a housekeeping gene, being ubiquitously expressed, and the encoded protein has been localized to the nucleolus.	
			MED22	185641	The encoded proteins are localized to the cytoplasm.	

			RPL7A	185640	This gene rearranges with the trk proto-oncogene to form the chimeric oncogene trk-2h, which encodes an oncoprotein consisting of the N terminus of ribosomal protein L7a fused to the receptor tyrosine kinase domain of trk	
			SURF1	185620	This gene encodes a protein localized to the inner mitochondrial membrane and thought to be involved in the biogenesis of the cytochrome c oxidase complex.	Leigh syndrome, due to COX deficiency
			SURF2	185630	This gene is located in the surfeit gene cluster, a group of very tightly linked genes that do not share sequence similarity.	
			SURF4	185660	This gene is located in the surfeit gene cluster, which is comprised of very tightly linked housekeeping genes that do not share sequence similarity. The encoded protein is a	

					conserved integral membrane protein containing multiple putative transmembrane regions.	
			REXO4	602930	REX4, RNA exonuclease 4 homolog (S. cerevisiae)	
			ADAMTS13	604134	This gene encodes a member of the ADAMTS (a disintegrin and metalloproteinase with thrombospondin motif) protein family.	Thrombotic thrombocytopenic purpura, familial
			C9orf7	613104	calcium channel flower domain containing 1	
			SLC2A6	606813	solute carrier family 2 (facilitated glucose transporter), member 6	
	21q22.3	deletion				
			HSF2BP	604554	heat shock transcription factor 2 binding protein	
			RRP1B	610654	ribosomal RNA processing 1 homolog B (S. cerevisiae)	
			PDXK	179020	The protein encoded by this gene phosphorylates vitamin B6, a step required for the conversion of vitamin	

					B6 to pyridoxal-5-phosphate, an important cofactor in intermediary metabolism.	
			CSTB	601145	This gene encodes a stefin that functions as an intracellular thiol protease inhibitor.	Epilepsy, progressive myoclonic 1A (Unverricht and Lundborg)
			RRP1	610653	The protein encoded by this gene is the putative homolog of the yeast ribosomal RNA processing protein RRP1. The encoded protein is involved in the late stages of nucleologenesis at the end of mitosis, and may be required for the generation of 28S rRNA.	
**44**	**11q23.3**	**deletion**				
			POU2F3	607394	This gene encodes a member of the POU domain family of transcription factors. POU domain transcription factors bind to a specific octamer DNA motif and regulate cell type-	

					specific differentiation pathways.	
			ARHGEF12	604763	The encoded protein may form a complex with G proteins and stimulate Rho-dependent signals.	Leukemia, acute myeloid
	16q21	duplication				
			HERPUD1	608070	This gene may play a role in both UPR and ERAD	
			CETP	118470	Cholesteryl ester transfer protein (CETP) transfers cholesteryl esters between lipoproteins. CETP may effect susceptibility to atherosclerosis.	Hyperalphalipoproteinemia [High density lipoprotein cholesterol level QTL 10]
			NLRC5	613537	This gene encodes a member of the caspase recruitment domain-containing NLR family.	
			CPNE2	604206	Calcium-dependent membrane-binding proteins may regulate molecular events at the interface of the cell membrane and cytoplasm.	
			CPNE2	600340	plasmolipin	
			CCL22	602957	The product of this gene binds to chemokine receptor CCR4.	

			CX3CL1	601880	chemokine (C-X3-C motif) ligand 1	
			CCL17	601520	The product of this gene binds to chemokine receptors CCR4 and CCR8.	
			CIAPIN1	608943	cytokine induced apoptosis inhibitor 1	
			COQ9	612837	The encoded protein is likely necessary for biosynthesis of coenzyme Q10, as mutations at this locus have been associated with autosomal-recessive neonatal-onset primary coenzyme Q10 deficiency	Coenzyme Q10 deficiency
			POLR2C	180663	This gene encodes the third largest subunit of RNA polymerase II, the polymerase responsible for synthesizing messenger RNA in eukaryotes.	
			DOK4	608333	docking protein 4	
			GPR56	604110	This gene encodes a member of the G protein-coupled receptor family.	Polymicrogyria, bilateral frontoparietal

**45**	**17q21.31**	**duplication**				
			DHX8	600396	DEAD box proteins, characterized by the conserved motif Asp-Glu-Ala-Asp (DEAD), are putative RNA helicases. They are implicated in a number of cellular processes involving alteration of RNA secondary structure such as translation initiation, nuclear and mitochondrial splicing, and ribosome and spliceosome assembly.	
			ETV4	600711	ets variant 4	
			MEOX1	600147	This gene encodes a member of a subfamily of non-clustered, diverged, antennapedia-like homeobox-containing genes.	
	Xq13.1	deletion				
			EDA	300451	The protein encoded by this gene is a type II membrane protein that can be cleaved by furin to produce a secreted form.	Ectodermal dysplasia, anhidrotic, X-linked Tooth agenesis, selective, X-linked 1

**46**	**2q36.1**	**duplication**				
			EPHA4	602188	This gene belongs to the ephrin receptor subfamily of the protein-tyrosine kinase family.	
	10q26.3	duplication				
			ADAM8	602267	This gene encodes a member of the ADAM (a disintegrin and metalloprotease domain) family.	
			CALY	604647	The protein encoded by this gene is a type II single transmembrane protein.	
			PRAP1	609776	proline-rich acidic protein 1	
			ECHS1	602292	The protein encoded by this gene functions in the second step of the mitochondrial fatty acid beta-oxidation pathway.	
			SPRN	610447	shadow of prion protein homolog (zebrafish)	
	Xp22.31	deletion				
			NLGN4X	300427	This gene encodes a member of a family of neuronal cell surface proteins.	Mental retardation, X-linked {Asperger syndrome susceptibility, X-linked 2}

						{Autism susceptibility, X-linked 2}
**47**	**7q11.23**	**duplication**				
			UPK3B	611887	UPK3B is a minor component of the apical plaques of mammalian urothelium that binds and dimerizes with uroplakin-1b (UPK1B; MIM 602380), one of the major conserved urothelium membrane proteins.	
			POMZP3	600587	This gene appears to have resulted from a fusion of DNA sequences derived from 2 distinct loci, specifically through the duplication of two internal exons from the POM121 gene and four 3' exons from the ZP3 gene.	
	17q21.31	duplications				
			DHX8	600396	DEAD box proteins, characterized by the conserved motif Asp-Glu-Ala-Asp (DEAD), are putative RNA helicases.	
			ETV4	600711	ets variant 4	

			MEOX1	600147	This gene encodes a member of a subfamily of non-clustered, diverged, antennapedia-like homeobox-containing genes.	
**48**	**Xq28**	**deletion**				
			RAB39B	300774	This gene encodes a member of the Rab family of proteins.	Mental retardation, X-linked 72
			CLIC2	300138	Chloride channels are a diverse group of proteins that regulate fundamental cellular processes including stabilization of cell membrane potential, transepithelial transport, maintenance of intracellular pH, and regulation of cell volume.	
**49**	**6q27**	**duplication**				
			RNASET2	612944	ribonuclease T2	Leukoencephalopathy, cystic, without megalencephaly
			FGFR1OP	605392	This gene encodes a largely hydrophilic protein postulated to be a	Myeloproliferative disorder

					leucine-rich protein family member	
	13q12.13	duplication				
			SPATA13	613324	spermatogenesis associated 13	
	21q22.3	duplication				
			ABCG1	603076	The protein encoded by this gene is a member of the superfamily of ATP-binding cassette (ABC) transporters.	
			TFF3	600633	trefoil factor 3 (intestinal)	
			TFF2	182590	trefoil factor 2	
	Xp11.22	deletion				
			SMC1A	300040	Proper cohesion of sister chromatids is a prerequisite for the correct segregation of chromosomes during cell division.	Cornelia de Lange syndrome 2
			HSD17B10	300256	This gene encodes 3-hydroxyacyl-CoA dehydrogenase type II, a member of the short-chain dehydrogenase/reductase superfamily. The gene product is a	17-beta-hydroxysteroid dehydrogenase X deficiency Mental retardation, X-linked 17/31, microduplication Mental retardation, X-linked

					mitochondrial protein that catalyzes the oxidation of a wide variety of fatty acids, alcohols, and steroids.	syndromic 10
			HUWE1	300697	This gene encodes a member of the HECT E3 ubiquitin ligase family.	Mental retardation, X-linked syndromic, Turner type
	Xq28					
			OPN1MW	300821	This gene encodes for a light absorbing visual pigment of the opsin gene family.	Blue cone monochromacy<br>Colorblindness, deutan
			TEX28	300092	testis expressed 28	
			TKTL1	300044	The protein encoded by this gene is a transketolase that acts as a homodimer and catalyzes the conversion of sedoheptulose 7-phosphate<br>and D-glyceraldehyde 3-phosphate to D-ribose 5-phosphate and D-xylulose 5-phosphate.	
			FLNA	300017	The protein encoded by this gene is an actin-binding protein that crosslinks actin filaments and links	Cardiac valvular dysplasia, X-linked<br>FG syndrome 2

					actin filaments to membrane glycoproteins.	Frontometaphyseal dysplasia<br>Heterotopia, periventricular<br>Heterotopia, periventricular, ED variant<br>Intestinal pseudoobstruction, neuronal<br>Melnick-Needles syndrome<br>Otopalatodigital syndrome, type I<br>Otopalatodigital syndrome, type II<br>Terminal osseous dysplasia
			EMD	300384	Emerin is a serine-rich nuclear membrane protein and a member of the nuclear lamina-associated protein family.	Emery-Dreifuss muscular dystrophy 1, X-linked
			RPL10	312173	This gene encodes a ribosomal protein that is a component of the 60S subunit.	{Autism, susceptibility to, X-linked 5}
			TAZ	300394	This gene encodes a protein that is	Barth syndrome

					expressed at high levels in cardiac and skeletal muscle.	Cardiomyopathy, dilated, 3A<br>Left ventricular noncompaction, X-linked
**50**	**11p15.5**	**deletion**				
			KCNQ1OT1	604115	KCNQ1 opposite strand/antisense transcript 1 (non-protein coding)	Beckwith-Wiedemann syndrome
			KCNQ1	607542	This gene encodes a voltage-gated potassium channel required for repolarization phase of the cardiac action potential.	Atrial fibrillation, familial, 3<br>Jervell and Lange-Nielsen syndrome<br>Long QT syndrome-1<br>Short QT syndrome-2<br>{Long QT syndrome 1, acquired, susceptibility to}
	17p11.2	deletion				
			GRAP	604330	This gene encodes a member of the GRB2/Sem5/Drk family.	
	Xp11.22	deletion				
			SMC1A	300040	structural maintenance of chromosomes 1A	Cornelia de Lange syndrome 2
	Xq21.1	deletion				

			ATRX	300032	The protein encoded by this gene contains an ATPase/helicase domain, and thus it belongs to the SWI/SNF family of chromatin remodeling proteins.	Alpha-thalassemia myelodysplasia syndrome, somatic Alpha-thalassemia/mental retardation syndrome Mental retardation-hypotonic facies syndrome, X-linked
	Xq24	deletion				
			SEPT6	300683	This gene is a member of the septin family of GTPases. Members of this family are required for cytokinesis.	
			RPL39	601904	This gene is co-transcribed with the U69 small nucleolar RNA gene, which is located in its second intron. As is typical for genes encoding ribosomal proteins, there are multiple processed pseudogenes of this gene dispersed through the genome.	
			UPF3B	300298	This gene encodes a protein that is part of a post-splicing multiprotein complex involved in both mRNA	Mental retardation, X-linked, syndromic 14

					nuclear export and mRNA surveillance.	
			NDUFA1	300078	The human NDUFA1 gene codes for an essential component of complex I of the respiratory chain, which transfers electrons from NADH to ubiquinone.	Mitochondrial complex I deficiency
			AKAP14	300462	The A-kinase anchor proteins (AKAPs) are a group of structurally diverse proteins, which have the common function of binding to the regulatory subunit of protein kinase A (PKA) and confining the holoenzyme to discrete locations within the cell. This gene encodes a member of the AKAP family.	
			NKAP	300766	This gene encodes a protein that is involved in the activation of the ubiquitous transcription factor NF-kappaB.	
	Xq28	deletion				

			OPN1MW	300821	This gene encodes for a light absorbing visual pigment of the opsin gene family.	Blue cone monochromacy<br>Colorblindness, deutan
			TEX28	300092	testis expressed 28	
			TKTL1	300044	The protein encoded by this gene is a transketolase that acts as a homodimer and catalyzes the conversion of sedoheptulose 7-phosphate<br>and D-glyceraldehyde 3-phosphate to D-ribose 5-phosphate and D-xylulose 5-phosphate.	
			FLNA	300017	The protein encoded by this gene is an actin-binding protein that crosslinks actin filaments and links actin filaments to membrane glycoproteins.	Cardiac valvular dysplasia, X-linked<br>FG syndrome 2<br>Frontometaphyseal dysplasia<br>Heterotopia, periventricular<br>Heterotopia, periventricular, ED variant<br>Intestinal

						pseudoobstruction, neuronal<br>Melnick-Needles syndrome<br>Otopalatodigital syndrome, type I<br>Otopalatodigital syndrome, type II<br>Terminal osseous dysplasia
			EMD	300384	Emerin is a serine-rich nuclear membrane protein and a member of the nuclear lamina-associated protein family.	Emery-Dreifuss muscular dystrophy 1, X-linked
			RPL10	312173	This gene encodes a ribosomal protein that is a component of the 60S subunit.	{Autism, susceptibility to, X-linked 5}
			TAZ	300394	This gene encodes a protein that is expressed at high levels in cardiac and skeletal muscle.	Barth syndrome<br>Cardiomyopathy, dilated, 3A<br>Left ventricular noncompaction, X-linked
			ATP6AP1	300197	This gene encodes a component of a multisubunit enzyme (1 mDa MW) that mediates acidification of	

					eukaryotic intracellular organelles.	
			GDI1	300104	GDP dissociation inhibitors are proteins that regulate the GDP-GTP exchange reaction of members of the rab family, small GTP-binding proteins of the ras superfamily, that are involved in vesicular trafficking of molecules between cellular organelles.	Mental retardation, X-linked 41
			FAM50A	300453	This gene belongs to the FAM50 family. The encoded protein is highly conserved in length and sequence across different species. It is a basic protein containing a nuclear localization signal, and may function as a DNA-binding protein or a transcriptional factor.	
			PLXNA3	300022	The protein encoded by this gene is a member of the plexin class of proteins.	
			LAGE3	300060	L antigen family, member 3	

			UBL4A	312070	ubiquitin-like 4A	
			SLC10A3	312090	solute carrier family 10 (sodium/bile acid cotransporter family), member 3	
			FAM3A	300492	family with sequence similarity 3, member A	
			G6PD	305900	This gene encodes glucose-6-phosphate dehydrogenase.	Favism<br>G6PD deficiency<br>Hemolytic anemia due to G6PD deficiency
			IKBKG	300248	This gene encodes the regulatory subunit of the inhibitor of kappaB kinase (IKK) complex, which activates NF-kappaB resulting in activation of genes involved in inflammation, immunity, cell survival, and other pathways.	Ectodermal dysplasia, hypohidrotic, with immune deficiency<br>Ectodermal, dysplasia, anhidrotic, lymphedema and immunodeficiency<br>Immunodeficiency, isolated<br>Incontinentia pigmenti, type II<br>Invasive pneumococcal disease, recurrent isolated, 2

						{Atypical mycobacteriosis, familial}
			CTAG1A	300657	cancer/testis antigen 1A	
			CTAG1B	300156	The protein encoded by this gene is an antigen that is overexpressed in many cancers but that is also expressed in normal testis.	
	Xq13.3	deletion				
			ZDHHC15	300576	The protein encoded by this gene belongs to the DHHC palmitoyltransferase family.	Mental retardation, X-linked 91
	Xq25	deletion				
			SMARCA1	300012	This gene encodes a member of the SWI/SNF family of proteins.	
**51**	**14q32.33**	**deletion**				
			INF2	610982	This gene represents a member of the formin family of proteins. It is considered a diaphanous formin due to the presence of a diaphanous	Charcot-Marie-Tooth disease, dominant intermediate E Glomerulosclerosis, focal

					inhibitory domain located at the N-terminus of the encoded protein.	segmental, 5
			ADSSL1	612498	adenylosuccinate synthase like 1	
			SIVA1	605567	This gene encodes a protein with an important role in the apoptotic (programmed cell death) pathway induced by the CD27 antigen, a member of the tumor necrosis factor receptor (TFNR) superfamily.	
			AKT1	164730	v-akt murine thymoma viral oncogene homolog 1	Breast cancer, somatic<br>Colorectal cancer, somatic<br>Ovarian cancer, somatic<br>Proteus syndrome, somatic<br>{Schizophrenia, susceptibility to}
			ZBTB42	613915	zinc finger and BTB domain containing 42	
	21q22.3	deletion				
			ABCG1	603076	The protein encoded by this gene is a member of the superfamily of ATP-binding cassette (ABC) transporters.	

			TFF3	603076	Members of the trefoil family are characterized by having at least one copy of the trefoil motif, a 40-amino acid domain that contains three conserved disulfides.	
			TFF2	182590	The encoded protein inhibits gastric acid secretion.	
	Xq28	deletion				
			OPN1LW	300822	This gene encodes for a light absorbing visual pigment of the opsin gene family.	Blue cone monochromacy Colorblindness, protan
			OPN1MW	300821	This gene encodes for a light absorbing visual pigment of the opsin gene family. The encoded protein is called green cone photopigment or medium-wavelength sensitive opsin.	Blue cone monochromacy Colorblindness, protan
			TEX28	300092	testis expressed 28	
			TKTL1	300044	The protein encoded by this gene is a transketolase that acts as a homodimer and catalyzes the conversion of sedoheptulose 7-	

					phosphate and D-glyceraldehyde 3-phosphate to D-ribose 5-phosphate and D-xylulose 5-phosphate.	
			FLNA	300017	The protein encoded by this gene is an actin-binding protein that crosslinks actin filaments and links actin filaments to membrane glycoproteins.	Cardiac valvular dysplasia, X-linked<br>FG syndrome 2<br>Frontometaphyseal dysplasia<br>Heterotopia, periventricular<br>Heterotopia, periventricular, ED variant<br>Intestinal pseudoobstruction, neuronal<br>Melnick-Needles syndrome<br>Otopalatodigital syndrome, type I<br>Otopalatodigital syndrome, type II<br>Terminal osseous dysplasia
	Xq28	deletion				
			HAUS7	300540	This gene encodes a protein	

					identified by interaction with ubiquitin C-terminal hydrolase 37, which functions to edit polyubiquitin chains on ubiquitinated substrates.	
			BGN	301870	The protein encoded by this gene is a small cellular or pericellular matrix proteoglycan that is closely related in structure to two other small proteoglycans, decorin and fibromodulin.	
			ATP2B3	300014	The protein encoded by this gene belongs to the family of P-type primary ion transport ATPases characterized by the formation of an aspartyl phosphate intermediate during the reaction cycle.	
			FAM58A	300708	The protein encoded by this gene contains a cyclin-box-fold domain which suggests it may have a role in controlling nuclear cell division cycles.	STAR syndrome

			DUSP9	300134	The protein encoded by this gene is a member of the dual specificity protein phosphatase subfamily.	
			PNCK	300680	pregnancy up-regulated non-ubiquitously expressed CaM kinase	
**52**	**1p36.33**	**deletion**				
			SKI	164780	This gene encodes the nuclear protooncogene protein homolog of avian sarcoma viral (v-ski) oncogene.	
	5q13.2	deletion				
			SMN2	601627	survival of motor neuron 2, centromeric	{Spinal muscular atrophy, type III, modifier of}
			SERF1A	603011	small EDRK-rich factor 1A (telomeric)	
			SMN1	600354	survival of motor neuron 1, telomeric	Spinal muscular atrophy-1<br>Spinal muscular atrophy-2<br>Spinal muscular atrophy-3<br>Spinal muscular atrophy-4
			NAIP	600355	NLR family, apoptosis inhibitory protein	
			GTF2H2	601748	general transcription factor IIH, polypeptide 2, 44kDa	

	Yq11.223	deletion				
			RBMY1A1	400006	This gene encodes a protein containing an RNA-binding motif in the N-terminus and four SRGY (serine, arginine, glycine, tyrosine) boxes in the C-terminus.	
			PRY2	400041	This gene is located in the nonrecombining portion of the Y chromosome, and expressed specifically in testis. It encodes a protein which has a low degree of similarity to protein tyrosine phosphatase, non-receptor type 13.	
			TTTY5	400038	testis-specific transcript, Y-linked 5 (non-protein coding)	
			TTTY6	400039	testis-specific transcript, Y-linked 6 (non-protein coding)	
			PRY	400019	This gene is located in the nonrecombining portion of the Y chromosome, and expressed specifically in testis. It encodes a	

					protein which has a low degree of similarity to protein tyrosine phosphatase, non-receptor type 13.	
			TTTY17A	400040	testis-specific transcript, Y-linked 17A (non-protein coding)	
			TTTY4	400037	testis-specific transcript, Y-linked 4 (non-protein coding)	
			BPY2	400013	basic charge, Y-linked, 2	
			DAZ1	400003	This gene is a member of the DAZ gene family and is a candidate for the human Y-chromosomal azoospermia factor (AZF).	?Sertoli-cell-only syndrome
			DAZ2	400026	This gene is a member of the DAZ gene family and is a candidate for the human Y-chromosomal azoospermia factor (AZF).	
**53**	**1q44**	**deletion**				
			SMYD3	608783	This gene encodes a histone methyltransferase which functions in RNA polymerase II complexes by an interaction with a specific RNA	

					helicase.	
			TFB2M	607055	transcription factor B2, mitochondrial	
			CNST	613439	consortin, connexin sorting protein	
			AHCTF1	610853	AT hook containing transcription factor 1	
			ZNF124	194631	This gene encodes a member of the Kruppel-type (C2H2) zinc finger protein family.	
			ZNF496	613911	zinc finger protein 496	
			NLRP3	606416	This gene encodes a pyrin-like protein containing a pyrin domain, a nucleotide-binding site (NBS) domain, and a leucine-rich repeat (LRR) motif.	CINCA syndrome<br>Cold-induced autoinflammatory syndrome, familial<br>Muckle-Wells syndrome
			OR13G1	611677	olfactory receptor, family 13, subfamily G, member 1	
	6q26	deletion				
			QKI	609590	The protein encoded by this gene is an RNA-binding protein that regulates pre-mRNA splicing, export of mRNAs	

				from the nucleus, protein translation, and mRNA stability.	
	16p13.3p13.12	duplication			
			POLR3K	606007	This gene encodes a small essential subunit of RNA polymerase III, the polymerase responsible for synthesizing transfer and small ribosomal RNAs in eukaryotes.	
			MPG	156565	N-methylpurine-DNA glycosylase	
			NPRL3	600928	The function of the encoded protein is not known.	
			HBZ	142310	Zeta-globin is an alpha-like hemoglobin. The zeta-globin polypeptide is synthesized in the yolk sac of the early embryo, while alpha-globin is produced throughout fetal and adult life.	
			HBM	609639	hemoglobin, mu	

			HBA2	141850	hemoglobin, alpha 2	Erythrocytosis<br>Heinz body anemia<br>Hemoglobin H disease, nondeletional<br>Hypochromic microcytic anemia<br>Thalassemia, alpha-
			HBA1	141800	hemoglobin, alpha 1	Erythremias, alpha-<br>Heinz body anemias, alpha-<br>Hemoglobin H disease, nondeletional<br>Methemoglobinemias, alpha-<br>Thalassemias, alpha-
			HBQ1	142240	Theta-globin mRNA is found in human fetal erythroid tissue but not in adult erythroid or other nonerythroid tissue.	
			LUC7L	607782	LUC7-like (S. cerevisiae)	
			RGS11	603895	This protein inhibits signal transduction by increasing the	

					GTPase activity of G protein alpha subunits, thereby driving them into their inactive GDP-bound form.	
			ARHGDIG	602844	Rho GDP dissociation inhibitor (GDI) gamma	
			PDIA2	608012	protein disulfide isomerase family A, member 2	
			AXIN1	603816	This gene encodes a cytoplasmic protein which contains a regulation of G-protein signaling (RGS) domain and a dishevelled and axin (DIX) domain.	Caudal duplication anomaly Hepatocellular carcinoma, somatic
			MRPL28	604853	This gene encodes a 39S subunit protein, a part of which was originally isolated by its ability to recognize tyrosinase in an HLA-A24-restricted fashion.	
			NME4	601818	non-metastatic cells 4, protein expressed in	
			RAB11FIP3	608738	RAB11 family interacting protein 3 (class II)	

			SOLH	603267	This gene encodes a protein containing zinc-finger-like repeats and a calpain-like protease domain.	
			PIGQ	605754	This gene is involved in the first step in glycosylphosphatidylinositol (GPI)-anchor biosynthesis.	
			WFIKKN1	608021	This gene encodes a secreted multidomain protein consisting of a signal peptide, a WAP domain, a follistatin domain, an immunoglobulin domain, two tandem Kunitz domains, and an NTR domain.	
			RHOT2	613889	This gene encodes a member of the Rho family of GTPases.	
			RHBDL1	603264	rhomboid, veinlet-like 1 (Drosophila)	
			STUB1	607207	STUB1, or CHIP, is a ubiquitin ligase/cochaperone that participates in protein quality control by targeting a broad range of chaperone protein substrates for degradation	
			FBXL16	609082	F-box and leucine-rich repeat protein	

					16	
			METRN	610998	Meteorin regulates glial cell differentiation and promotes the formation of axonal networks during neurogenesis	
			NARFL	611118	nuclear prelamin A recognition factor-like	
			MSLN	601051	This gene encodes a precursor protein that is cleaved into two products, megakaryocyte potentiating factor and mesothelin.	
			CHTF18	613201	CHTF18, CHTF8 (MIM 613202), and DCC1 (DSCC1; MIM 613203) are components of an alternative replication factor C (RFC) (see MIM 600404) complex that loads PCNA (MIM 176740) onto DNA during S phase of the cell cycle	
			GNG13	607298	Heterotrimeric G proteins, which consist of alpha (see MIM 139320),	

					beta (see MIM 139380), and gamma subunits, function as signal transducers for the 7-transmembrane-helix G protein-coupled receptors.	
			LMF1	611761	The protein encoded by this gene resides in the endoplasmic reticulum, and is involved in the maturation and transport of lipoprotein lipase through the secretory pathway.	Lipase deficiency, combined
			SOX8	605923	This gene encodes a member of the SOX (SRY-related HMG-box) family of transcription factors involved in the regulation of embryonic development and in the determination of the cell fate.	
			SSTR5	182455	The protein encoded by this gene is one of the SSTRs, which is a multi-pass membrane protein and belongs to the G-protein coupled receptor 1 family.	Somatostatin analog, resistance to

			C1QTNF8	614147	C1q and tumor necrosis factor related protein 8	
			CACNA1H	607904	This gene encodes a T-type member of the alpha-1 subunit family, a protein in the voltage-dependent calcium channel complex.	16p13.3 {Epilepsy, childhood absence, susceptibility to, 6} {Epilepsy, idiopathic generalized, susceptibility to, 6}
			TPSG1	609341	Tryptases comprise a family of trypsin-like serine proteases, the peptidase family S1. Tryptases are enzymatically active only as heparin-stabilized tetramers, and they are resistant to all known endogenous proteinase inhibitors.	
			TPSB2	191081	tryptase beta 2 (gene/pseudogene)	
			TPSAB1	191080	tryptase alpha/beta 1	
			TPSD1	609272	tryptase delta 1	
			UBE2I	601661	This gene encodes a member of the	

					E2 ubiquitin-conjugating enzyme family.	
			BAIAP3	604009	This p53-target gene encodes a brain-specific angiogenesis inhibitor.	
			GNPTG	607838	This gene encodes the gamma sunbunit of the N-acetylglucosamine-1-phosphotransferase complex.	Mucolipidosis III gamma
			CLCN7	602727	The product of this gene belongs to the CLC chloride channel family of proteins.	Osteopetrosis, autosomal dominant 2 Osteopetrosis, autosomal recessive 4
			C16orf38	613442	pentraxin 4, long	
			TELO2	611140	This gene encodes a protein that functions as an S-phase checkpoint protein in the cell cycle.	
			TMEM204	611002	transmembrane protein 204	
			MAPK8IP3	605431	The protein encoded by this gene shares similarity with the product of Drosophila syd gene, required for the functional interaction of kinesin I with axonal cargo.	

			NME3	601817	non-metastatic cells 3, protein expressed in	
			MRPS34	611994	This gene encodes a 28S subunit protein.	
			EME2	610886	essential meiotic endonuclease 1 homolog 2 (S. pombe)	
			SPSB3	611659	splA/ryanodine receptor domain and SOCS box containing 3	
			NUBP2	610779	nucleotide binding protein 2	
			IGFALS	601489	The protein encoded by this gene is a serum protein that binds insulin-like growth factors, increasing their half-life and their vascular localization.	Acid-labile subunit, deficiency of
			HAGH	138760	The enzyme encoded by this gene is classified as a thiolesterase and is responsible for the hydrolysis of S-lactoyl-glutathione to reduced glutathione and D-lactate.	[Glyoxalase II deficiency]
			SEPX1	606216	This gene encodes a selenoprotein, which contains a selenocysteine (Sec) residue at its active site.	

			NDUFB10	603843	NADH dehydrogenase (ubiquinone) 1 beta subcomplex, 10, 22kDa	
			RPS2	603624	ribosomal protein S2	
			TBL3	605915	The protein encoded by this gene has sequence similarity with members of the WD40 repeat-containing protein family.	
			NOXO1	611256	NADPH oxidase organizer 1	
			GFER	600924	growth factor, augmenter of liver regeneration	Myopathy, mitochondrial progressive, with congenital cataract, hearing loss, and developmental delay
			SYNGR3	603927	This gene encodes an integral membrane protein.	
			NPW	607997	The product of this gene is processed into 23- and 30-amino acid neuropeptides that bind and activate two G-protein coupled receptors in the central nervous system.	
			SLC9A3R2	606553	This gene encodes a member of the NHERF family of PDZ scaffolding	

				proteins. These proteins mediate many cellular processes by binding to and regulating the membrane expression and protein-protein interactions of membrane receptors and transport proteins.	
			NTHL1	602656	The protein encoded by this gene is a DNA N-glycosylase of the endonuclease III family. Like a similar protein in E. coli, the encoded protein has DNA glycosylase activity on DNA substrates containing oxidized pyrimidine residues and has apurinic/apyrimidinic lyase activity.	
			TSC2	191092	The protein associates with hamartin in a cytosolic complex, possibly acting as a chaperone for hamartin.	Lymphangioleiomyomatosis, somatic<br>Tuberous sclerosis-2
			MIR1225	611621	microRNA 1225	
			PKD1	601313	This gene encodes a member of the polycystin protein family.	Polycystic kidney disease, adult type I

			RAB26	605455	Members of the RAB protein family, including RAB26, are important regulators of vesicular fusion and trafficking.	
			TRAF7	606692	Tumor necrosis factor (TNF; see MIM 191160) receptor-associated factors, such as TRAF7, are signal transducers for members of the TNF receptor superfamily (see MIM 191190).	
			CASKIN1	612184	CASK interacting protein 1	
			MLST8	612190	MTOR associated protein, LST8 homolog (S. cerevisiae)	
			PGP	172280	phosphoglycolate phosphatase	
			E4F1	603022	The zinc finger protein encoded by this gene is one of several cellular transcription factors whose DNA-binding activities are regulated through the action of adenovirus E1A.	
			DNASE1L2	602622	deoxyribonuclease I-like 2	

			DCI	600305	This gene encodes a member of the hydratase/isomerase superfamily. The protein encoded is a key mitochondrial enzyme involved in beta-oxidation of unsaturated fatty acids.	
			RNPS1	606447	This gene encodes a protein that is part of a post-splicing multiprotein complex involved in both mRNA nuclear export and mRNA surveillance.	
			CCNF	600227	This gene encodes a member of the cyclin family.	
			NTN3	602349	netrin 3	
			TBC1D24	613577	This gene encodes a protein with a conserved domain, referred to as the TBC domain, characteristic of proteins which interact with GTPases.	Myoclonic epilepsy, infantile, familial
			ATP6V0C	108745	This gene encodes a component of vacuolar ATPase (V-ATPase), a multisubunit enzyme that mediates	

	[redacted]	[redacted]			acidification of eukaryotic intracellular organelles.	
	[redacted]	[redacted]	CEMP1	611113	cementum protein 1	
	[redacted]	[redacted]	PDPK1	605213	3-phosphoinositide dependent protein kinase-1	
	[redacted]	[redacted]	KCTD5	611285	potassium channel tetramerisation domain containing 5	
	[redacted]	[redacted]	PRSS27	608018	protease, serine 27	
	[redacted]	[redacted]	SRRM2	606032	serine/arginine repetitive matrix 2	
	[redacted]	[redacted]	TCEB2	600787	This gene encodes the protein elongin B, which is a subunit of the transcription factor B (SIII) complex.	
	[redacted]	[redacted]	PRSS33	613797	protease, serine, 33	
	[redacted]	[redacted]	PRSS21	608159	This gene encodes a cell-surface anchored serine protease, which is a member of the trypsin family of serine proteases.	
	[redacted]	[redacted]	PRSS22	609343	This gene encodes a member of the trypsin family of serine proteases. The enzyme is expressed in the	

	[redacted]	[redacted]			airways in a developmentally regulated manner.	
	[redacted]	[redacted]	KREMEN2	609899	This gene encodes a high-affinity dickkopf homolog 1 (DKK1) transmembrane receptor. A similar protein in mouse functions interacts with with DKK1 to block wingless (WNT)/beta-catenin signaling.	
	[redacted]	[redacted]	PKMYT1	602474	The protein encoded by this gene is a member of the serine/threonine protein kinase family.	
	[redacted]	[redacted]	TNFRSF12 A	605914	tumor necrosis factor receptor superfamily, member 12A	
	[redacted]	[redacted]	MMP25	608482	Proteins of the matrix metalloproteinase (MMP) family are involved in the breakdown of extracellular matrix in normal physiological processes, such as embryonic development, reproduction, and tissue remodeling, as well as in disease processes, such	

					as arthritis and metastasis.	
			IL32	606001	This gene encodes a member of the cytokine family.	
			ZNF205	603436	zinc finger protein 205	
			ZNF213	608387	zinc finger protein 213	
			OR1F1	603232	Olfactory receptors interact with odorant molecules in the nose, to initiate a neuronal response that triggers the perception of a smell.	
			ZNF200	603231	zinc finger protein 200	
			MEFV	608107	This gene encodes a protein, also known as pyrin or marenostrin, that is an important modulator of innate immunity.	Familial Mediterranean fever, AD<br>Familial Mediterranean fever, AR
			ZNF263	604191	zinc finger protein 263	
			TIGD7	612969	The protein encoded by this gene belongs to the tigger subfamily of the pogo superfamily of DNA-mediated transposons in humans.	
			ZNF75A	601473	zinc finger protein 75a	

	[redacted]	[redacted]	ZNF174	603900	zinc finger protein 174	
	[redacted]	[redacted]	NAA60	614246	N(alpha)-acetyltransferase 60, NatF catalytic subunit	
	[redacted]	[redacted]	SLX4	613278	This gene encodes a structure-specific endonuclease subunit.	Fanconi anemia, complementation group P
	[redacted]	[redacted]	DNASE1	125505	This gene encodes a member of the DNase family.	{Systemic lupus erythematosus, susceptibility to}
	[redacted]	[redacted]	TRAP1	606219	TRAP1 is a mitochondrial HSP90 protein.	
	[redacted]	[redacted]	CREBBP	600140	The protein encoded by this gene has intrinsic histone acetyltransferase activity and also acts as a scaffold to stabilize additional protein interactions with the transcription complex.	Rubinstein-Taybi syndrome
	[redacted]	[redacted]	ADCY9	603302	Adenylate cyclase is a membrane bound enzyme that catalyses the formation of cyclic AMP from ATP.	
	[redacted]	[redacted]	SRL	604992	sarcalumenin	
	[redacted]	[redacted]	TFAP4	600743	transcription factor AP-4 (activating	

					enhancer binding protein 4)	
			GLIS2	608539	This gene is a member of the GLI-similar zinc finger protein family and encodes a nuclear transcription factor with five C2H2-type zinc finger domains.	Nephronophthisis 7
			VASN	608843	vasorin	
			CORO7	611668	coronin 7	
			DNAJA3	608382	This gene encodes a member of the DNAJ/Hsp40 protein family.	
			HMOX2	141251	Heme oxygenase, an essential enzyme in heme catabolism, cleaves heme to form biliverdin, which is subsequently converted to bilirubin by biliverdin reductase, and carbon monoxide, a putative neurotransmitter.	
			C16orf5	610503	hromosome 16 open reading frame 5	
			MGRN1	607559	mahogunin ring finger 1, E3 ubiquitin protein ligase	
			SEPT12	611562	This gene encodes a guanine-	

					nucleotide binding protein and member of the septin family of cytoskeletal GTPases.	
			GLYR1	610660	glyoxylate reductase 1 homolog (Arabidopsis)	
			UBN1	609771	ubinuclein 1	
			PPL	602871	The protein encoded by this gene is a component of desmosomes and of the epidermal cornified envelope in keratinocytes.	
			NAGPA	607985	This gene encodes the enzyme that catalyzes the second step in the formation of the mannose 6-phosphate recognition marker on lysosomal hydrolases.	
			ALG1	605907	The enzyme encoded by this gene catalyzes the first mannosylation step in the biosynthesis of lipid-linked oligosaccharides.	Congenital disorder of glycosylation, type Ik
			RBFOX1	605104	The protein binds to the C-terminus of ataxin-2 and may contribute to the	

	[redacted]	[redacted]			restricted pathology of spinocerebellar ataxia type 2 (SCA2).	
	[redacted]	[redacted]	TMEM114	611579	transmembrane protein 114	
	[redacted]	[redacted]	ABAT	137150	4-aminobutyrate aminotransferase (ABAT) is responsible for catabolism of gamma-aminobutyric acid (GABA), an important, mostly inhibitory neurotransmitter in the central nervous system, into succinic semialdehyde.	GABA-transaminase deficiency
	[redacted]	[redacted]	PMM2	601785	The protein encoded by this gene catalyzes the isomerization of mannose 6-phosphate to mannose 1-phosphate, which is a precursor to GDP-mannose necessary for the synthesis of dolichol-P-oligosaccharides.	Congenital disorder of glycosylation, type Ia
	[redacted]	[redacted]	USP7	602519	ubiquitin specific peptidase 7 (herpes virus-associated)	
	[redacted]	[redacted]	GRIN2A	138253	N-methyl-D-aspartate (NMDA) receptors are a class of ionotropic	Epilepsy with neurodevelopmental defects

	[redacted]	[redacted]			glutamate-gated ion channels.	
	[redacted]	[redacted]	ATF7IP2	613645	activating transcription factor 7 interacting protein 2	
	[redacted]	[redacted]	EMP2	602334	epithelial membrane protein 2	
	[redacted]	[redacted]	NUBP1	600280	nucleotide binding protein 1	
	[redacted]	[redacted]	CIITA	600005	This gene encodes a protein with an acidic transcriptional activation domain, 4 LRRs (leucine-rich repeats) and a GTP binding domain.	Bare lymphocyte syndrome, type II, complementation group A {Rheumatoid arthritis, susceptibility to}
	[redacted]	[redacted]	CLEC16A	611303	This gene encodes a member of the C-type lectin domain containing family.	
	[redacted]	[redacted]	SOCS1	603597	This gene encodes a member of the STAT-induced STAT inhibitor (SSI), also known as suppressor of cytokine signaling (SOCS), family.	
	[redacted]	[redacted]	TNP2	190232	transition protein 2 (during histone to protamine replacement)	
	[redacted]	[redacted]	PRM2	182890	Protamines are the major DNA-binding proteins in the nucleus of	

					sperm, and package the DNA in a volume less than 5% of a somatic cell nucleus	
			PRM1	182880	protamine 1	
			RMI2	612426	RMI2, RecQ mediated genome instability 2, homolog (S. cerevisiae)	
			LITAF	603795	Lipopolysaccharide is a potent stimulator of monocytes and macrophages, causing secretion of tumor necrosis factor-alpha (TNF-alpha) and other inflammatory mediators.	Charcot-Marie-Tooth disease, type 1C
			SNN	603032	stannin	
			BCAR4	613746	breast cancer anti-estrogen resistance 4 (non-protein coding)	
			GSPT1	139259	G1 to S phase transition 1	
			TNFRSF17	109545	The protein encoded by this gene is a member of the TNF-receptor superfamily.	
			SHISA9	613346	shisa homolog 9 (Xenopus laevis)	
			ERCC4	133520	The protein encoded by this gene	Xeroderma pigmentosum,

					forms a complex with ERCC1 and is involved in the 5' incision made during nucleotide excision repair.	group F<br>XFE progeroid syndrome
			MKL2	609463	MKL/myocardin-like 2	
	Xp22.31	deletion				
			STS	300747	The protein encoded by this gene catalyzes the conversion of sulfated steroid precursors to estrogens during pregnancy.	
	Xq12	deletion				
			OPHN1	300127	This gene encodes a Rho-GTPase-activating protein that promotes GTP hydrolysis of Rho subfamily members.	
	Xq22.3	deletion				
			AMMECR1	300195	The exact function of this gene is not known, however, submicroscopic deletion of the X chromosome including this gene, COL4A5, and FACL4 genes,<br>result in a contiguous gene deletion	

					syndrome, the AMME complex (Alport syndrome, mental retardation, midface hypoplasia, and elliptocytosis).	
**54**	**6p12.3**	**duplication**				
			EYA4	603550	This gene encodes a member of the eyes absent (EYA) family of proteins. The encoded protein may act as a transcriptional activator through its protein phosphatase activity, and it may be important for eye development, and for continued function of the mature organ of Corti. Mutations in this gene are associated with postlingual, progressive, autosomal dominant hearing loss at the deafness, autosomal dominant nonsyndromic sensorineural 10 locus. Defects in this gene are also associated with dilated cardiomyopathy 1J. Three transcript	Cardiomyopathy, dilated, 1J Deafness, autosomal dominant 10

					variants encoding distinct isoforms have been identified for this gene. [provided by RefSeq, Jul 2008]	
	Xp22.12	deletion				
			SH3KBP1	300374	This gene encodes an adapter protein that contains three N-terminal Src homology domains, a proline rich region and a C-terminal coiled-coil domain. The encoded protein facilitates protein-protein interactions and has been implicated in numerous cellular processes including apoptosis, cytoskeletal rearrangement, cell adhesion and in the regulation of clathrin-dependent endocytosis. Alternate splicing results in multiple transcript variants.[provided by RefSeq, May 2010]	
